# Anthonoic Acids A–C, Sulfated and *N*-(2-Hydroxyethyl)-Substituted Lipidic Amino Acids from the Marine Sponge *Antho ridgwayi* with In Vitro Cytoprotective Activities

**DOI:** 10.3390/molecules31010036

**Published:** 2025-12-22

**Authors:** Alla G. Guzii, Ekaterina K. Kudryashova, Larisa K. Shubina, Tatyana N. Makarieva, Alexander S. Menshov, Roman S. Popov, Ekaterina A. Yurchenko, Evgeny A. Pislyagin, Ekaterina A. Chingizova, Boris B. Grebnev, Vladimir A. Shilov, Valentin A. Stonik

**Affiliations:** 1 G.B. Elyakov Pacific Institute of Bioorganic Chemistry, Far Eastern Branch of the Russian Academy of Scienes, Pr. 100-let Vladivostoku 159, Vladivostok 690022, Russia; catrinog.81@mail.ru (E.K.K.); shubina@piboc.dvo.ru (L.K.S.); almenshov1990@gmail.com (A.S.M.); prs_90@mail.ru (R.S.P.); pislyagin@hotmail.com (E.A.P.); martyyas@mail.ru (E.A.C.); grebnev_bor@mail.ru (B.B.G.); stonik@piboc.dvo.ru (V.A.S.); 2 A.V. Zhirmunsky National Scientific Center of Marine Biology, Far Eastern Branch of the Russian Academy of Sciences, 17 Palchevskogo Str., Vladivostok 690041, Russia; shilvl@yandex.ru

**Keywords:** lipoamino acids, marine sponge, ischemia, reperfusion, P2X7 receptor, reactive oxygen species, superoxide dismutase

## Abstract

Anthonoic acids A–C (**1**–**3**), the first representatives of sulfated and *N*-(2-hydroxyethyl)-substituted lipidic *α*-amino acids, were isolated along with their plausible precursor, anthamino acid A (**4**), from the marine sponge *Antho ridgwayi*. The structures of these compounds were determined using the analysis of 1D and 2D NMR and HR ESI mass spectra. A structural feature of **1**–**4**, compared to all previously known lipidic amino acids, is the presence of a sulfate group near the end opposite the amino acid terminus. At a concentration of 1 µM, anthonoic acids A–C (**1**–**3**) effectively protected H9c2 and SH-SY5Y cells in biotests, which modeled hypoxia induced by the addition of CoCl_2_ to the medium and damage caused by ischemia/reperfusion. These natural products act via the Nrf2-mediated pathway by reducing intracellular ROS levels, accompanied by the upregulation of SOD activity, which is controlled by the Nrf2 transcriptional factor. Anthonoic acids A–C (**1**–**3**) do not activate the transcriptional activity of NF-*κ*B but inhibit ATP-induced cell damage and calcium influx, indicating the involvement of P2X7 receptors in the cytoprotective effect of anthonoic acids A–C.

## 1. Introduction

Heart and blood vessel diseases continue to be the main cause of mortality globally. The global prevalence of ischemic stroke increased from 34.7 × 10^6^ cases in 1990 to 69.9 × 10^6^ cases in 2021 [1]. Positive trends in mortality reduction in some countries have been destroyed by the COVID-19 pandemic, and we need to address this problem again. Heart and neuronal cells have increased susceptibility to oxygen and nutrient deprivation [2], making the search for cardio- and neuroprotective molecules with anti-hypoxic and anti-ischemic activities promising. Marine natural compounds and their derivatives (MNPDs) are of great interest in the search for new pharmaceuticals, including anti-hypoxic and anti-ischemic compounds. By 2025, 15 pharmaceuticals based on MNPD have been approved, and more than two dozen have been studied in phases I, II, and III of clinical trials [3]. Due to the fact that some extremophilic marine micro- and macroorganisms inhabit low-oxygen concentration and nutrient-deprived conditions, they can adapt to these conditions via biosynthesis of specialized metabolites, which may be promising as new pharmaceuticals [4,5]. In our earlier studies, isomalabaricane glycosides, rhabdastrellosides A and B, with cytoprotective effects against CoCl_2_-mimic hypoxia, were obtained from the marine sponge *Rhabdastrella globostellata* [6], and ω-glycosylated fatty acid amides, toporosides A–D, demonstrating anti-inflammatory cardioprotective activities, were isolated from the marine sponge *Stelodoryx toporoki* [7].

Some in vitro models are available to study the possible anti-ischemic and anti-hypoxic compounds. CoCl_2_ treatment has been used for hypoxia-mimicking in spite of certain restrictions [8]. CoCl_2_ treatment induces oxidative stress and cell damage [9] and changes intracellular Ca^2+^ and ATP levels [10], similar to oxygen starvation. A high level of reactive oxygen species (ROS) generation may induce pyroptosis via the canonical inflammatory NF-kB/HIF-1α pathway [11] and triggers the P2X7-dependent non-canonical inflammatory pathway [12] as a result of ATP release from damaged cells.

Cellular ischemia develops as a result of a limited supply of nutrition and oxygen to cells, and the abrupt resumption of nutrition against the background of cell conversion to an anaerobic type of metabolism is no less damaging. In vitro ischemia/reperfusion damage is simulated in cultured cells using physiological salt solutions free of metabolic substrates [13].

In the course of searching for anti-hypoxic and anti-ischemic marine sponge-derived natural products [7], we isolated three new sulfated *N*-substituted lipidic α-amino acids, anthonoic acids A–C (**1**–**3**), and a lipidic α-amino acid, anthamino acid A (**4**), from the previously chemically unstudied Northern Pacific marine sponge *Antho ridgwayi*. No data have been reported on secondary metabolites from *Antho* sponges. Only linearmycin A and B, naramycin B, and actiphenol were identified in *Streptomyces* spp. isolated from the sponge *Antho dichotoma* collected from the Trondheim fjord (Norway) [14]. Herein, we report the isolation and structural determination of natural products containing long-chain hydrocarbon substituents attached to amino acids. Moreover, the study of cytoprotective effects of anthonoic acids A–C (**1**–**3**) against in vitro ischemia/reperfusion and chronic hypoxia in normal rat H9c2 cardiomyocytes and human neuroblastoma SH-SY5Y cells was evaluated.

## 2. Results

### 2.1. Isolation and Structure Elucidation of the Anthonoic Acids A–C and Anthamino Acid A

**Figure 1:**
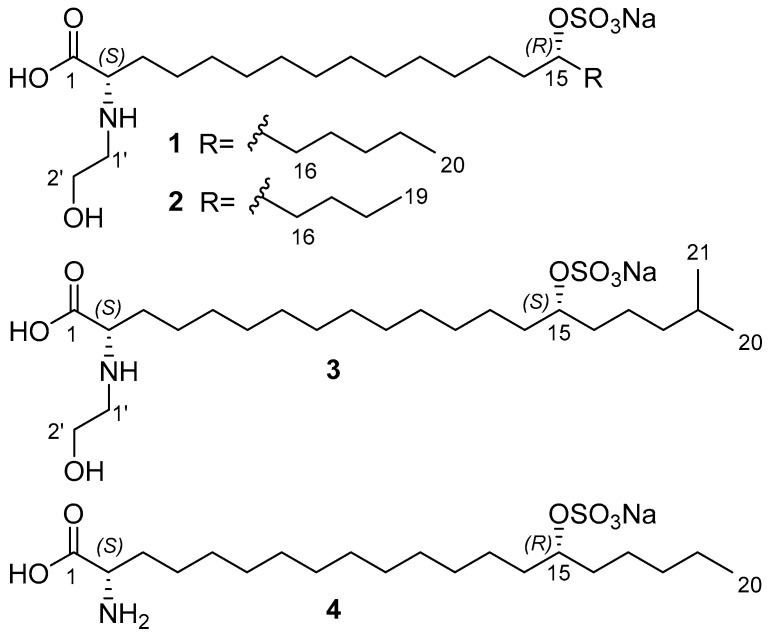
Structures of anthonoic acids A–C (**1**–**3**) and anthamino acid A (**4**).

The ethanolic extract of *A*. *ridgwayi* was concentrated and separated by ODS flash chromatography, followed by reverse-phase HPLC to yield anthonoic acids A–C (**1**–**3**) and anthamino acid A (**4**) (Figure 1).

The molecular formula of **1** was determined to be C_22_H_44_NO_7_SNa from the peak of the cationized molecule at *m*/*z* 512.2626 [M_Na_+Na]^+^ and the peak of the decationized molecule at *m*/*z* 466.2835 [M–H]^−^ in the HRESIMS spectra. The peak at *m*/z 232.6382 in the negative ion mode HRESIMS corresponds to a doubly charged [M–2H]^2−^ ion.

The NMR spectra of **1** (CD_3_OD, Table 1) showed the presence of a nitrogenated methine (*δ*_C_ 63.1; *δ*_H_ 3.68 t, *J* = 5.8 Hz), an oxygenated methine (*δ*_C_ 80.9; *δ*_H_ 4.33 quint, *J* = 6.0 Hz) flanked by polymethylene chains, a carboxyl (*δ*_C_ 173.0), one terminal methyl (*δ*_C_ 14.4, *δ*_H_ 0.91 t, *J* = 7.0 Hz), and a hydroxyethyl group attached to nitrogen (*δ*_C_ 49.9 and 58.0; *δ*_H_ 3.14 m and 3.80 t, *J* = 5.3 Hz).

**Figure 2:**
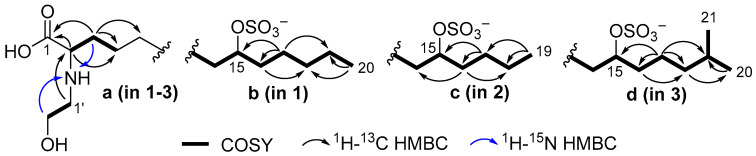
Partial structures of **1**–**3** with selected COSY and HMBC correlations.

The ^1^H–^13^C HMBC correlations from H-2 (*δ*_H_ 3.68) to carboxyl carbon C-1 (*δ*_C_ 173.0) and methylene carbons at *δ*_C_ 31.2 (C-3) and 26.04 (C-4) and the ^1^H–^15^N HMBC cross peak H_2_-3 (*δ*_H_ 1.89)/N (*δ*_N_ 46.6) (Figure 2, substructure **a**), together with the presence of the fragment ion with *m*/*z* 422.2951 [M_Na_–Na–CO_2_]^−^ (Table S1, Supporting Information) in the (–)HRESIMS/MS spectrum of the parent ion at *m*/*z* 466 [M_Na_–Na]^−^ confirmed the presence of an *α*-amino acid moiety.

The *N*-hydroxyethyl residue was confirmed by COSY correlations between H_2_-1′ and H_2_-2′ and ^1^H–^15^N HMBC correlations between H-2′ (*δ*_H_ 3.80) and N (*δ*_N_ 46.6). The ^1^H–^13^C HMBC correlation from H_2_-1′ (*δ*_H_ 3.14) to C-2 (*δ*_C_ 63.1) indicates the attachment of *N*-hydroxyethyl to C-2. The unusually deshielded oxymethine signals (*δ*_C_ 80.9; *δ*_H_ 4.33) suggest the presence of a sulfate group, which is consistent with the molecular formula. The fragment ion peaks at *m*/*z* 386.3284 [M_Na_–Na–SO_3_]^−^ (Table S1) and *m*/*z* 96.9592 [HSO_4_]^−^ in the (–)HRESIMS/MS spectrum of the parent ion at *m*/*z* 466 [M_Na_–Na]^−^ (Figure S10) confirmed the presence of a sulfate group.

**Figure 3:**
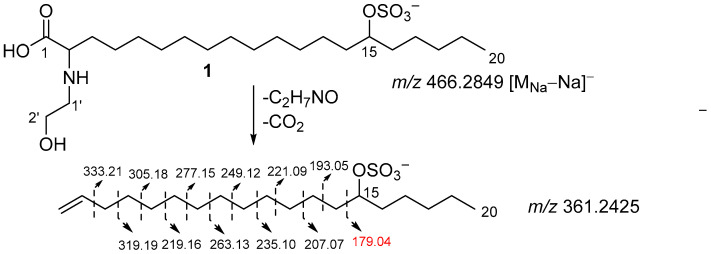
Fragmentation of **1** in (–)HRESIMS/MS.

The position of the sulfate group was assigned based on COSY experiments and HMBC correlations (Figure 2, substructure **b**). The COSY correlations belonging to aliphatic chain revealed the corresponding sequences of protons at C14 to C20. The HMBC cross-peaks H_2_-17/C-15 and C-19, H_2_-16/C-18, and H_3_-20/C-18 clearly indicate the presence of a sulfate group at C-15. The fragmentation in the (–)HRESIMS/MS of **1** (Figure 3, Table S1), particularly the fragment peak at *m*/*z* 179, confirmed the location of the sulfate group at C-15.

The above-mentioned data show that anthonoic acid A (**1**) is 2-(2-hydroxyethylamino)-15-(sulfooxy)eicosanoic acid.

The molecular formula of anthonoic acid B (**2**) was determined to be C_21_H_42_NO_7_SNa by HRESIMS measurement of the [M–H]^−^ peak at *m*/*z* 452.2679. The MS data showed that the molecular mass of **2** was 14 Da less than that of **1**, suggesting that the hydrocarbon chain of **2** was shorter by one CH_2_ group. The NMR spectroscopic data for **2** were similar to those for **1,** except for the *ω*-3 carbon signal at δ_C_ 33.1, which was shielded to 28.3, and the absence of the signal of one of the methylene groups at δ_C_ 25.7 (Table 1). The COSY and HMBC correlations placed the sulfate group at C-15. (Figure 2, substructure **c**). The fragmentation pattern of **2** in the (–)HRESIMS/MS and the fragment peak at *m*/*z* 165 confirmed the C-15 position of the sulfate group (Figure S21, Table S2). Therefore, anthonoic acid B (**2**) was proven to be 2-(2-hydroxyethylamino)-15-(sulfooxy)nonadecanoic acid.

The molecular formula of anthonoic acid C (**3**) was determined to be C_23_H_46_NO_7_SNa from the HRESIMS measurement of the [M–H]^−^ peak at *m*/*z* 480.2997. According to the MS data, the molecular mass of **3** was 14 Da greater than that of **1**. The spectroscopic properties of **3** were similar to those of **1** (Table 1). The difference between the NMR spectra of **1** and **3** was in the methyl group signals. The ^1^H NMR spectrum of **3** showed a doublet signal at *δ*_H_ 0.89, d (*J* = 6.7 Hz, 6H), indicating an *iso-*fatty acid chain terminus instead of the triplet at *δ*_H_ 0.91, t (*J* = 7 Hz, 3H), characteristic of *n*-fatty acid chain termini. The COSY and HMBC correlations (Figure 2, substructure **d**) and the fragmentation pattern of **3** and the fragment peak at 193 *m*/*z* in the (–)HRESIMS/MS (Figure S31, Table S3) indicated the C-15 position of the sulfate group. Thus, the structure of anthonoic acid C (**3**) was determined as 2-(2-hydroxyethylamino)-19-methyl-15-(sulfooxy)eicosanoic acid.

Anthamino acid A (**4**) was isolated as a mixture of **4** and **1** (75% of the main component). The molecular formula was determined by HRESIMS as C_20_H_40_NO_6_SNa, *m*/*z* 468.2375 [M_Na_+Na]^+^ and *m*/*z* 422.2582 [M_Na_–Na]^−^. MS data showed that the molecular formula of **4** was C_2_H_4_O, one unit less than that of **1**, suggesting that the hydroxyethyl unit on the amino acid terminus was absent. Analysis of the NMR spectra showed the presence of a nitrogenated methine (*δ*_C_ 56.1; *δ*_H_ 3.56 m) in **4**. A comparison of the NMR and MS data of **4** and **1** showed that antaminic acid A (**4**) differs from **1** by the presence of an amino group without a hydroxyethyl substituent. The fragmentation in the (–)HRESIMS/MS of the parent ion at *m*/*z* 422.2582 [M_Na_–Na]^−^, particularly the fragment peak at *m*/*z* 179, the same as for **1,** confirmed the location of the sulfate group at C-15.

**Figure 4:**
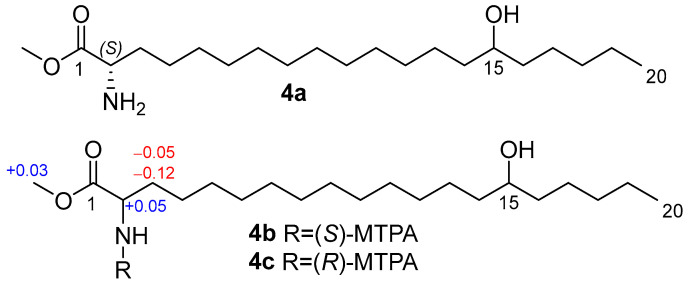
Methyl ester **4a** and MTPA amides **4b** and **4c**, and the Δ*δ^SR^* (*δ_S_* − *δ_R_*) values of **4b**,**c**.

To determine the absolute configuration at C-2, we prepared methyl ester **4a** by methanolysis followed by reverse-phase HPLC, then synthesized MTPA amides **4b** and **4c** and applied the modified Mosher’s method [15]. The observed chemical shift differences Δ*δ^SR^* (*δ_S_* − *δ_R_*) for H-2, H-3a, H-3b, and OCH_3_ between **4b** and **4c** indicate the 2*S* configuration (Figure 4).

Likely, anthamino acid A (**4**) is a biosynthetic precursor of anthonoic acid A (**1**) isolated from the same marine sponge sample. Therefore, the absolute configurations of **1**–**3** were proposed as 2*S* based on biogenetic considerations.

**Figure 5:**
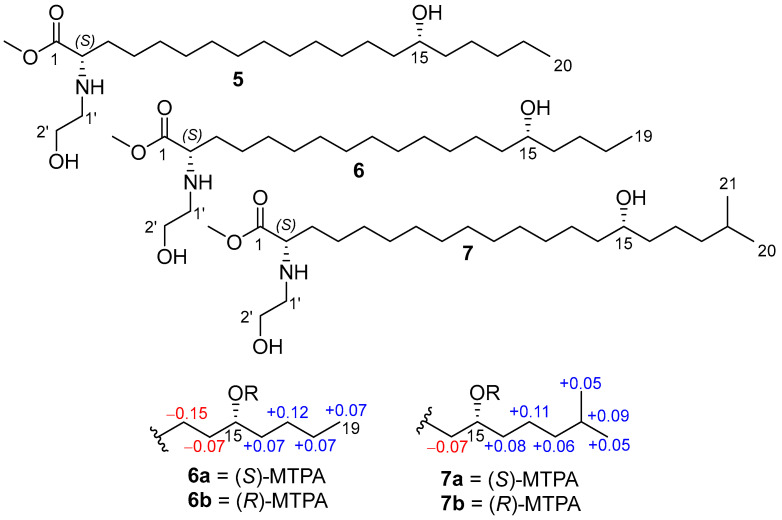
Methyl esters **5**–**7** and MTPA esters (**6a**, **6b**, **7a**, **7b**), and the Δ*δ^SR^* (*δ_S_* − *δ_R_*) values of **6a**,**b**, and **7a**,**b**.

To establish the absolute configuration of the C-15 stereogenic centers in **1**–**3**, (*S*)-MTPA and (*R*)-MTPA esters of desulfated methyl esters **5**–**7** were prepared (Figure 5).

The absolute configuration at C-15 in anthonoic acid A (**1**) was unable to be determined by using Mosher’s method due to the lack of chemical shift differences Δ*δ^SR^* for the adjacent methylene groups.

The COSY correlations and 1D selective TOCSY spectra of MTPA derivatives **6a**,**b** and **7a**,**b** clearly indicate the Δ*δ^SR^* values. The observed chemical shift differences Δ*δ^SR^* for H_2_-14 and H_2_-16 between **6a** and **6b**, **7a** and **7b** (Figure 4) indicate the 15*R* configuration for **2** and 15*S* for **3**. The absolute configuration of **1** was proposed as 15*R* (similar to **2**) based on biogenetic considerations.

### 2.2. Cytoprotective Activity of the Anthonoic Acids A–C (1–3)

#### 2.2.1. Influence on the Viability of H9c2 and SH-SY5Y Cells

**Figure 6:**
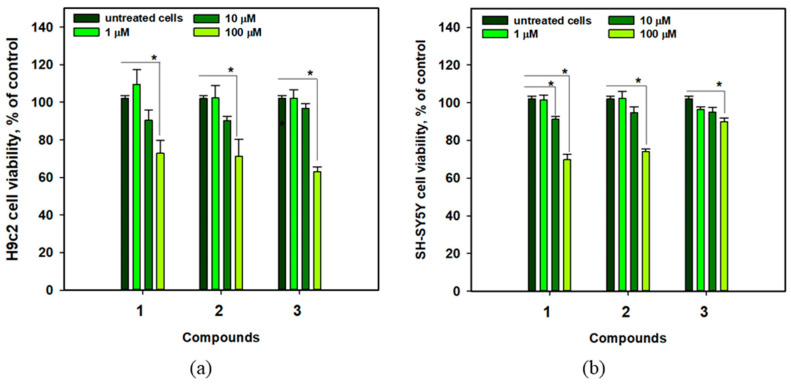
Viability of H9c2 (**a**) and SH-SY5Y (**b**) cells treated with anthonoic acids A–C (**1**–**3**) at different concentrations. Data are presented as mean ± standard error of the mean (SEM). The asterisk (*) indicates statistically significant differences between groups (*p* < 0.05).

The viability of cardiomyocytes H9c2 and neuroblastoma SH-SY5Y cells treated with compounds **1**–**3** is shown in Figure 6. Anthonoic acids A–C (**1**–**3**) at a concentration of 1 µM did not affect the viability of H9c2 and SH-SY5Y cells, while the compounds at a concentration of 10 µM weakly decreased the viability of the cells by 3.3–9.8%. At 100 µM, all compounds decreased the viability of H9c2 and SH-SY5Y cells by approximately 30%. Therefore, **1**, **2**, and, especially, **3** are less toxic to cardiomyocytes at concentrations below 10 μM. In the subsequent experiments, all compounds were tested at a nontoxic concentration of 1 µM.

#### 2.2.2. The Effects of 1–3 Against in Vitro Acute Ischemia/Reperfusion and Chronic Hypoxia

**Figure 7:**
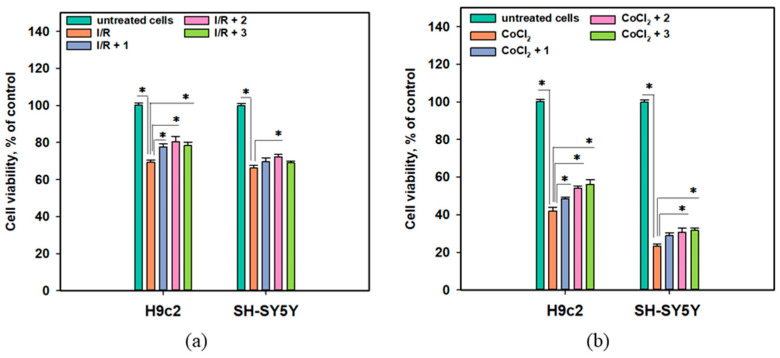
(**a**) Influence of anthonoic acids A–C (**1**–**3**) (1 µM) on the viability of I/R-treated H9c2 and SH-SY5Y cells. (**b**) Effect of compounds **1**–**3** (1 µM) on the viability of CoCl_2_-treated H9c2 and SH-SY5Y cells. Data are presented as mean ± SEM. The asterisk (*) indicates a statistically significant difference (*p* < 0.05).

The cytoprotective properties of anthonoic acids A–C (**1**–**3**) were investigated in in vitro acute ischemia/reperfusion (I/R) and CoCl_2_-mimic chronic hypoxia biotests using H9c2 cardiomyocytes and SH-SY5Y neuroblastoma cell lines (Figure 7).

The viability of I/R-treated H9c2 and SH-SY5Y cells decreased by 30.7% and 33.6%, respectively, after 24 h (Figure 7a). Treatment of the cells with CoCl_2_ at a 500 µM concentration caused a dramatic decrease in the viability of neuronal SH-SY5Y cells after 24 h of exposure by 76.8%, while the viability of cardiomyocytes H9c2 decreased by 58.0% after 48 h of treatment (Figure 7b).

The protective effect of **1** was observed in the CoCl_2_-mimic hypoxia assay, in which **1** increased the viability of CoCl_2_-treated H9c2 cells by 15.2% but had a lesser effect on CoCl_2_-treated SH-SY5Y cells. Anthonoic acid B (**2**) increased the viability of both I/R-treated H9c2 and SH-SY5Y cells by 16.0% and 8.9%, respectively. In the CoCl_2_-mimic hypoxia model, compound **2** increased the viability of both CoCl_2_-treated H9c2 and SH-SY5Y cells by 29.0% and 32.3%, respectively. Anthonoic acid C (**3**) increased the viability of I/R-treated H9c2 cells by 12.9%, whereas its effect on I/R-treated SH-SY5Y cells was not significant. In addition, compound **3** significantly increased the viability of CoCl_2_-treated H9c2 and SH-SY5Y cells by 33.5% and 36.2%, respectively.

#### 2.2.3. Influence of Anthonoic Acids A–C on Intracellular ROS Levels and SOD Activity

**Figure 8:**
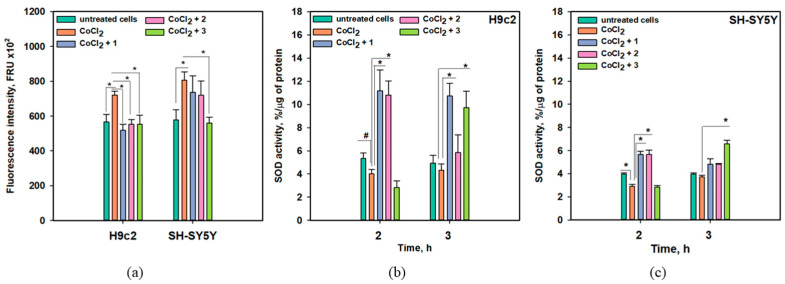
Influence of anthonoic acids A–C (**1**–**3**) (1 µM) on intracellular ROS levels in CoCl_2_-treated H9c2 and SH-SY5Y cells after 3 h (**a**). Influence of compounds **1**–**3** (1 µM) on SOD activity in CoCl_2_-treated H9c2 (**b**) and SH-SY5Y cells (**c**). Data are presented as mean ± SEM. The asterisk (*) indicates a statistically significant difference (*p* < 0.05). # indicates statistically significant differences (*p* < 0.10).

The effects of anthonoic acids A–C (**1**–**3**) on intracellular ROS levels after 3 h of treatment of H9c2 and SH-SY5Y cells with CoCl_2_ are presented in Figure 8a, and their effects on superoxide dismutase (SOD) activity after 2 and 3 h of treatment with CoCl_2_ were also examined (Figure 8b,c).

A significant increase in intracellular ROS levels of 27.2% and 39.8% was detected in CoCl_2_-treated H9c2 and SH-SY5Y cells, respectively (Figure 8a). Anthonoic acid C (**3**) significantly decreased ROS levels in both CoCl_2_-treated H9c2 and SH-SY5Y cells by 23.3% and 30.5%, respectively. Anthonoic acids A (**1**) and B (**2**) decreased the ROS levels in CoCl_2_-treated H9c2 cells by 23.1% and 28.2%, respectively, while their anti-ROS effect in CoCl_2_-treated SH-SY5Y cells was observed but was not statistically significant (Figure 8a).

Administration of CoCl_2_ for 2 h decreased SOD activity in both H9c2 and SH-SY5Y cells by 24.4% and 26.7%, respectively (Figure 8b,c). In CoCl_2_-treated H9c2 cells, anthonoic acid A (**1**) induced an increase in SOD activity of more than 200% after treatment for 2 or 3 h. Anthonoic acid B (**2**) also increased SOD activity by more than two times after 2 h of CoCl_2_ treatment. Anthonoic acid C (**3**) did not affect SOD activity in H9c2 cells after 2 h of CoCl_2_ treatment but increased SOD activity by more than two times after 3 h of CoCl_2_ treatment. In CoCl_2_-treated SH-SY5Y cells, the effect of the compounds was less. Anthonoic acids A (**1**) and B (**2**) increased SOD activity by 93.8% and 94.9%, respectively, after 2 h of CoCl_2_ treatment, and this effect diminished in the following hour (Figure 8c). Anthonoic acid C (**3**) increased SOD activity only by 76.5% after 3 h of CoCl_2_ treatment.

In hypoxia, electron transfer via the mitochondrial electron transport chain decelerates, raising the probability of electrons being inadvertently transferred to molecular oxygen. This results in the formation of highly reactive superoxide anions (O_2_^−^), which contribute to oxidative stress [16,17]. In ischemia, cellular metabolism moves toward anaerobic conditions, resulting in the buildup of long-chain fatty acids within cells. During reperfusion or reoxygenation, there is a sudden surge in O_2_, accompanied by the additional production of reactive oxygen species (ROS) and the onset of acute oxidative stress [11]. Detox enzymes, such as SOD, constitute the initial defense of the intracellular antioxidant protection system, regulated via the Nrf2/Keap1 pathway. Thus, anthonoic acids A–C (**1**–**3**) can decrease intracellular ROS levels in CoCl_2_-treated cells via the upregulation of SOD activity.

#### 2.2.4. Influence of Anthonoic Acids A–C on the Transcriptional Activity of NF-*κ*B

**Figure 9:**
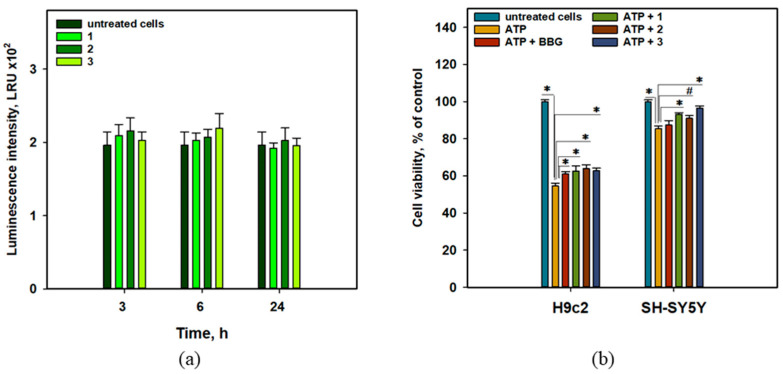
Influence of anthonoic acids A–C (**1**–**3**) (1 µM) on luminescence in JB6-Luc NF-*κ*B cells (**a**). Effect of anthonoic acids A–C (**1**–**3**) on the viability of ATP-treated H9c2 and SH-SY5Y cells after 24 h (**b**). Brilliant blue G-250 (BBG) was used as a positive control. The data are presented as a mean ± SEM. The asterisk (*) indicates statistically significant differences (*p* < 0.05). # indicates statistically significant differences (*p* < 0.1).

Hypoxia- or ischemia-induced ROS generation can promote cell damage via the NF-*κ*B/HIF-1*α*-dependent pathway [11]. Therefore, we examined the effects of anthonoic acids A–C (**1**–**3**) on NF-*κ*B transcriptional activity. For this purpose, JB6 Cl41 cells, which stably express NF-*κ*B and luciferase reporter genes, were used (Figure 9a).

Compounds **1**–**3** did not show any statistically significant effect on luciferase activity, which was measured as luminescence intensity using a luciferin-containing kit, in JB6-Luc NF-*κ*B cells for 3, 6, or 24 h. Thus, the cytoprotective effects of anthonoic acids A–C (**1**–**3**) are not due to their influence on the canonical NF-*κ*B-dependent inflammatory pathway (Figure 9a).

#### 2.2.5. Influence of Anthonoic Acids A–C on ATP-Mediated Viability and Ca^2+^ Influx

As mentioned in the Introduction, hypoxia may trigger a P2X7-dependent non-canonical inflammatory pathway [10]. In this case, extracellular ATP induced modulation of P2X7 receptors, resulting in Ca^2+^ influx, inflammasome formation, and cell damage.

The effects of anthonoic acids A–C (**1**–**3**) on the viability of ATP-treated H9c2 and SH-SY5Y cells are presented in Figure 9b**.** Brilliant blue G-250 (BBG) is a well-known inhibitor of P2X receptors [18] and was used as a positive control in this test.

Treatment with ATP (4 mM) significantly decreased the viability of H9c2 and SH-SY5Y cells by 45.3% and 14.6%, respectively. Anthonoic acids A–C **(1**–**3**) statistically significantly increased the viability of ATP-treated H9c2 cells by 14.4-17.0%. In ATP-treated SH-SY5Y cells, compounds **1** and **3** increased viability by 9.1% and 13.1%, respectively, with a significance level of *p* < 0.05, while the effect of compound **2** (6.6%) was significant at *p* < 0.1.

**Figure 10:**
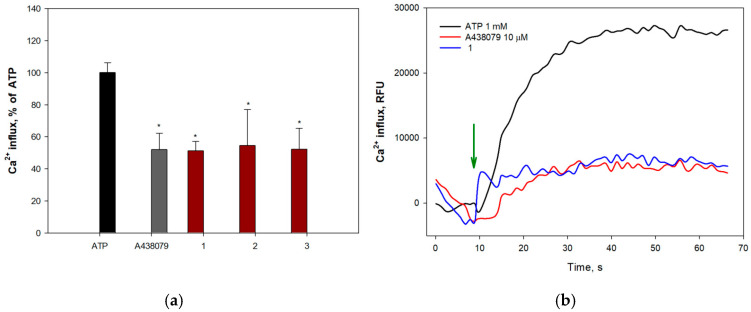
Effect of anthonoic acids A–C (**1**–**3**) at 10 µM on ATP-induced Ca^2+^ influx in H9c2 cells: (**a**) Effect of compounds **1**–**3**, A438079 (10 µM), on Ca^2+^ influx induced by ATP (1 mM) in H9c2 cells. (**b**) Representative curves of [Ca^2+^]i elevation induced by ATP (1 mM) alone or in the presence of compound **1** or standard P2X7 receptor blocker A438079 in H9c2 cells. Green arrow indicates the injection of ATP. Anthonoic acids A–C (**1**–**3**) were used at a concentration of 1.0 µM. Data are presented as mean ± SEM (n = 3); * *p* < 0.05 compared to ATP.

**Figure 11:**
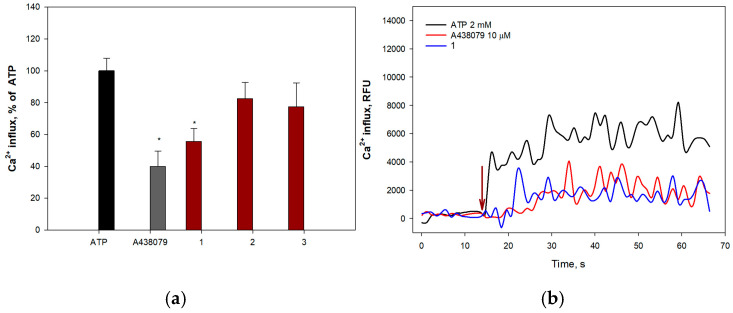
Effect of anthonoic acids A–C (**1**–**3**) on ATP-induced Ca^2+^ influx in SH-SY5Y cells: (**a**) Effect of compounds **1**–**3** and A438079 (10 µM) on Ca^2+^ influx induced by ATP (2 mM) in SH-SY5Y cells. (**b**) Representative curves of [Ca^2+^]i elevation induced by ATP (2 mM) alone or in the presence of compound **1** or the standard P2X7 receptor blocker A438079 in SH-SY5Y cells. Anthonoic acids A–C (**1**–**3**) were used at a concentration of 1.0 µM. Data are presented as mean ± SEM (n = 3); * *p* < 0.05 compared to ATP.

Extracellular ATP induces permeabilization of purinergic receptors P2X7Rs to calcium ions, leading to an increase in their intracellular concentration. To determine the ability of anthonoic acids A–C (**1**–**3**) to inhibit P2X7Rs, H9c2 and SH-SY5Y cells were loaded with the Ca^2+^-selective fluorescent probe Fluo-8 AM. The P2X7R blocker A438079 (10 μM) was used as a positive control. The effects of **1**–**3** on ATP-induced Ca^2+^ influx in H9c2 and SH-SY5Y cells are shown in Figure 10 and Figure 11, respectively.

All compounds at 1.0 and 10.0 µM concentrations significantly inhibited calcium influx in H9c2 cells, showing efficacy comparable to that of the standard blocker A438079. Anthonoic acid A (**1**) at 10 µM caused the greatest effect, reducing the influx of calcium ions by 70.1 ± 1.0%. Treatment of H9c2 cells with anthonoic acids B (**2**) and C (**3**) also inhibited ATP-induced calcium entry (Figure 10).

Moreover, compounds **1** and **2** significantly inhibited calcium influx into SH-SY5Y cells, showing efficacy comparable to that of the standard blocker A438079. Compound **2** demonstrated the greatest effect, significantly reducing the influx of calcium ions at a concentration of 10.0 μM by 63.3 ± 8.0%. Treatment of SH-SY5Y cells with compound **1** also led to inhibition of ATP-induced calcium entry (1.0 µM—44.2 ± 8.1%) (Figure 11a).

## 3. Discussion

α-Amino acid derivatives with long alkyl chain substituents are rare natural products. The isolated new natural products **1**–**3** were proven to be unusual lipidic amino acid derivatives, differing from all previously known compounds of this class in the presence of a sulfate group and *N*-(2-hydroxyethyl) residue. Previously known lipidic amino acid derivatives were found mainly in microorganisms and invertebrates. For example, two lipidic α-amino acids have been isolated from the zoanthid *Protopalythoa variabilis* [19,20]. The simplifungin, an antifungal antibiotic, was isolated from the culture of the fungal strain *Simplicillium minatense* FKI-4981 [21]. 2-Amino-9,13-dimethyl heptadecanoic acid was found in a *Streptomyces* strain isolated from marine sediment collected off Levinston Island, Antarctica [22]. In addition, highly functionalized sphingosine-like α-amino acids, such as sphingofungins [23,24], fumifungin [25], myriocin [26], and mycestericins [27], have been reported from the fungi *Aspergillus fumigatus* [23,25], *Paecilomyces variotii* [24], *Myriococcus albomyces* [26], and *Mycelia sterilia* [27]. Lipidic α-amino acids are also known as components of peptides, chlorofusin [28] from the fungus *Fusarium* sp., longicatenamycin [29] from the bacteria *Streptomyces diastaticus,* piperazimycins from *Streptomyces* sp. [30], and aspercryptins from the fungus *Aspergillus nidulans* [31]. This type of *α*-amino acid shows high cytotoxic [19], pro-apoptotic [20], antifungal [23,25,26], antibiotic [32], and immunosuppressive [27,33] activities. Chlorofusin inhibits the p53/MDM2 interaction and has potential in cancer therapy [28]. Some sphingosine-like *α*-amino acids inhibit serine palmitoyl transferases, enzymes that catalyze the initial step of sphingolipid biosynthesis [33,34]. Myriocin showed anti-inflammatory activity via NF-*κ*B-dependent pathway [35]. Moreover, myriocin post-conditioning significantly reduces ischemia/reperfusion lesions and inflammation in the first hours after reperfusion, and dietary administration of myriocin in the postinfarction phase ameliorates the myocardial remodeling and functioning [36].

The compounds **1**–**4** containing a sulfate group near one terminus and amino acid units at another terminus of the molecules are bipolar and have some similarity with bipolar so-called bola-like metabolites found in some microorganisms and marine invertebrates [37].

The obtained data suggest that anthonoic acids A–C (**1**–**3**) protect H9c2 and SH-SY5Y cells against ischemia/reperfusion and chronic hypoxia-induced damage via the Nrf2-mediated pathway. They reduce high intracellular ROS levels via the upregulation of SOD activity, which is controlled by the Nrf2 transcriptional factor. Interestingly, neuronal SH-SY5Y cells were more damaged during hypoxia than H9c2 cardiomyocytes and responded less to the protective effects of anthonoic acids A–C. This may be the result of the reduced antioxidant intracellular machinery in neuronal cells, which only weakly activates the detoxification and antioxidant enzymes/proteins via the Nrf2-ARE signaling axis [38]. It was reported that key detoxifying enzymes can be transferred from glioblastoma U373MG cells to neuronal SH-SY5Y cells in culture to protect the neuronal cells from reactive quinones [39].

Moreover, our experiments showed that **1**–**3** do not activate the transcriptional activity of NF-*κ*B but inhibit ATP-induced cell damage and calcium influx, indicating the involvement of P2X7 receptors in the cytoprotective effects of anthonoic acids A–C. Earlier, it was reported that inhibition of the P2X7 receptor by BBG, OxATP, or A438079 ameliorates transient global cerebral ischemia/reperfusion injury in rats by modulating inflammatory responses [40,41]. Previously, the inhibition of P2X7 was reported for synthetic 1-phenyl-6-azauracils, arylcarbohydrazides, cyanoguanidines, pyrazolacetamides, naphthoquinones, and adamantane amides, including one of them with an *N*-hydroxyethyl moiety, as the same in **1**–**3** [42]. Other analogous inhibitors have been found among natural products, including the tripyridine alkaloid niphatoxin C from the sponge *Callyspongia* sp. [43] and bisimidazo-pyrano-imidazole bromopyrrole ether alkaloids stylissadines A and B from *Stylissa flabellate* [44]. It should be noted that no information has been obtained so far about the effect of lipid α-amino acids on the P2X7 or Nrf2/Keap1 pathway, and our data are the first of their kind.

Many low-molecular-weight natural compounds are multi-targeted and can modulate several biochemical pathways. Our data show that compounds **1**–**3** may influence both the antioxidant pathways and the P2X7-dependent cascade.

## 4. Materials and Methods

### 4.1. General Procedures

Physical and spectroscopic data were obtained on a Perkin-Elmer 343 polarimeter (PerkinElmer Instruments, Waltham, MA, USA) for optical rotations. An IRTracer-100 FTIR spectrophotometer (Shimadzu, Kyoto, Japan), with Quest attenuated total reflection diamond accessory GS10801-B (Specac Ltd., Orpington, UK), was used for IR spectra. A Bruker Avance III-700 spectrometer (Bruker, Ettlingen, Germany) at 700, 175 and 70.93 MHz was used for the ^1^H, ^13^C and ^15^N NMR spectra, respectively. Chemical shifts (ppm) were internally referenced to the corresponding signals of CD_3_OD at δ_H_ 3.31/δ_C_ 49.0. ESI mass spectra (including HRESIMS) were obtained using a Bruker maXis Impact II Q-TOF mass spectrometer (Bruker Daltonics, Bremen, Germany) by direct infusion in MeOH. Low-pressure column liquid chromatography was performed using YMC*Gel ODS-A (YMC Co., Ltd., Kyoto, Japan). HPLC was performed using a Shimadzu Instrument equipped with the differential refractometer RID-10A (Shimadzu Corporation, Kyoto, Japan) on YMC-Pack ODS-A (250 × 10 mm, YMC Co., Ltd., Kyoto, Japan) column.

### 4.2. Animal Material

The sponge *Antho (Acarnia) ridgwayi* Stone, Lehnert & Hoff, 2019 (order Poecilosclerida, family Microcionidae, registration number PIBOC O47-142) was collected during the 47th scientific cruise of the R/V “Academic Oparin” in August 2015 near Bering Island in the Bering Sea (55°34′2″ N; 165°31′1″ E, depth 157 m) and identified by B. B. Grebnev and V. A. Shilov using the morphology of skeleton and spicules. A voucher specimen is stored in the Marine Invertebrate Collection of the G.B. Elyakov Pacific Institute of Bioorganic Chemistry FEB RAS (Vladivostok, Russia).

### 4.3. Extraction and Isolation

The fresh collection of the sponge *A. ridgwayi* was immediately extracted at room temperature with EtOH (200 mL × 3) for three 24 h periods. The EtOH extract of the sponge *A. ridgwayi* (dry weight 1.7 g) was concentrated and subjected to column chromatography on a reversed-phase YMC*Gel ODS-A with a stepped gradient from MeOH/H_2_O (40:60) to MeOH. The fraction that eluted with MeOH/H_2_O (40:60) was purified by HPLC (YMC-Pack ODS-A, 75:25:1% EtOH/H_2_O/1N NH_4_OAc) and gave pure anthonoic acid A (**1**, 7.3 mg, 0.43% based on dry weight of the sponge), anthonoic acid B (**2**, 1.8 mg, 0.11% based on dry weight of the sponge), anthonoic acid C (**3**, 5.1 mg, 0.30% based on dry weight of the sponge), and anthamino acid A (**4**, 4.5 mg mixture with compound **1**).

### 4.4. Compounds Characterization Data

*Anthonoic acid A* (**1**): colorless glass; [*α*]^20^_D_ + 5.7 (*c* 0.2, EtOH); IR (MeOH) *ν*_max_ 3397, 2924, 2854, 1674, 1437, 1206, 1183, 1134, 1054 cm^−1^; ^1^H and ^13^C NMR data (CD_3_OD), Table 1; HRESIMS *m*/*z* 466.2835 [M_Na_–Na]^−^ (calcd for C_22_H_44_NO_7_S, 466.2840, Δ2.0 ppm), *m*/*z* 232.6382 [M_Na_–Na–H]^2−^ (calcd for C_22_H_43_NO_7_S, 232.6386), *m*/*z* 512.2626 [M_Na_+Na]^+^ (calcd for C_22_H_44_NO_7_SNa_2_, 512.2628, Δ0.4 ppm).

*Anthonoic acid B* (**2**): colorless glass; [*α*]^20^_D_ + 6.0 (*c* 0.08, EtOH); IR (MeOH) *ν*_max_ 3386, 2920, 2851, 1582, 1465, 1396, 1203, 1053 cm^−1^; ^1^H and ^13^C NMR data (CD_3_OD), Table 1; HRESIMS *m*/*z* 452.2679 [M_Na_–Na]^−^ (calcd for C_21_H_42_NO_7_S, 452.2687, Δ1.9 ppm), *m*/*z* 498.2469 [M_Na_+Na]^+^ (calcd for C_21_H_42_NO_7_SNa_2_, 498.2472, Δ0.5 ppm).

*Anthonoic acid C* (**3**): colorless glass; [*α*]^20^_D_ + 1.9 (*c* 0.4, EtOH); IR (MeOH) *ν*_max_ 3364, 2919, 2848, 1576, 1465, 1400, 1211, 1054 cm^−1^; ^1^H and ^13^C NMR data (CD_3_OD), Table 1; HRESIMS *m*/*z* 480.2997 [M_Na_–Na]^−^ (calcd for C_23_H_46_NO_7_S, 480.3000, Δ0.5 ppm).

*Anthamino acid A* (**4**): selected ^1^H NMR (CD_3_OD, 700 MHz) δ 4.33 (quint, *J* = 5.8 Hz, H-15), 3.56 (m, H-2), 1.78 (m, H-3a, H-3b), 1.65 (m, H-14 or H-16), 1.62 (m, H-14 or H-16), 1.40 (m, H-13, H-17), 1.33 (m, H-19), 1.30 (m, H-18), 0.91 (t, *J* = 7.0 Hz, H-20); selected ^13^C NMR (CD_3_OD, 175 MHz) δ 80.9 (C-15), 56.1 (C-2), 35.3 (C-14, C-16), 31.8 (C-3), 26.3 (C-4), 26.0 (C-13), 25.8 (C-17), 23.6 (C-19), 14.3 (C-20); HRESIMS *m*/*z* 422.2582 [M–Na]^−^ (calcd for C_20_H_40_NO_6_S, 422.2582), *m*/*z* 468.2375 [M+Na]^+^ (calcd for C_20_H_40_NO_6_SNa_2_, 468.2366).

*Methanolysis of ***1**. 5% HCl in MeOH (0.5 mL) was added to **1** (5.0 mg), and the solution was heated for 4 h at 100 °C. Then the solvent was removed by a stream of N_2,_ and the residue was purified by HPLC (YMC-Pack ODS-A, 90:10:1% MeOH/H_2_O/NH_4_OAc) to give **5** (3.4 mg).

*Compound ***5**: HRESIMS *m*/*z* 402.3577 [M+H]^+^ (calcd for C_23_H_48_NO_4_, 402.3578), *m*/*z* 424.3394 [M+Na]^+^ (calcd for C_23_H_47_NO_4_Na, 424.3397).

*Methanolysis of ***2.** 5% HCl in MeOH (0.5 mL) was added to **2** (1.8 mg), and the solution was heated for 4h at 100 °C. Then the solvent was removed by a stream of N_2,_ and the residue was purified by HPLC (YMC-Pack ODS-A, 90:10:1% MeOH/H_2_O/NH_4_OAc) to give **6** (1.0 mg).

*Compound ***6**: HRESIMS *m*/*z* 388.3430 [M+H]^+^ (calcd for C_22_H_46_NO_4_, 388.3421), *m*/*z* 410.3249 [M+Na]^+^ (calcd for C_22_H_45_NO_4_Na, 410.3241).

*Methanolysis of ***3**. 5% HCl in MeOH (0.5 mL) was added to **3** (4.4 mg), and the solution was heated for 4 h at 100 °C. Then the solvent was removed by a stream of N_2,_ and the residue was purified by YMC*Gel ODS-A column (H_2_O→EtOH) to give **7** (1.0 mg).

*Compound ***7**: HRESIMS *m*/*z* 416.3745 [M+H]^+^ (calcd for C_24_H_50_NO_4_, 416.3734), *m*/*z* 438.3564 [M+Na]^+^ (calcd for C_24_H_49_NO_4_Na, 438.3554).

*Methanolysis of ***4**. 5% HCl in MeOH (0.5 mL) was added to a mixture of **4** and **1** (4.5 mg), and the solution was heated for 4 h at 100 °C. Then the solvent was removed by a stream of N_2,_ and the residue was purified by HPLC (YMC-Pack ODS-A, 90:10:1% MeOH/H_2_O/NH_4_OAc) to give pure **4a** (2.3 mg).

*Methyl ester of (2S)-2-Amino-15-(sulfooxy)eicosanoic acid* (**4a**): [*α*]^20^_D_ + 6.7 (*c* 0.2, EtOH); ^1^H NMR (CD_3_OD, 700 MHz) δ 3.72 (s, OCH_3_), 3.50 (m, H-15), 3.44 (m, H-2), 1.69 (m, H-3a), 1.60 (m, H-3b), 1.42 (m, H-14 or H-16), 1.37 (m, H-14 or H-16), 1.35 (m, H-4); 1.25-1.33 (br s, -CH_2_-), 0.91 (t, *J* = 7.0 Hz, H-20); ^13^C NMR (CD_3_OD, 175 MHz) δ 177.3 (C-1), 72.5 (C-15), 55.2 (C-2), 52.4 (OCH_3_), 38.4 (C-14, C-16), 35.7 (C-3), 33.1 (C-18), 26.5 (C-4), 23.7 (C-19), 14.4 (C-20); HRESIMS *m*/*z* 358.3330 [M+H]^+^ (calcd for C_21_H_44_NO_3_, 353.3316), *m*/*z* 380.3147 [M+Na]^+^ (calcd for C_21_H_43_NO_3_Na, 380.3135).

*Preparation of (S)-MTPA amide of ***4b**. Pyridine (2.5 µL) and (*R*)-MTPA chloride (1.0 µL) were added to a solution of **4a** (0.7 mg) in CHCl_3_ (1.0 mL). After stirring at room temperature for 2 h, the reaction mixture was concentrated in vacuo.

*(S)-MTPA amide* (**4b**): selected ^1^H NMR (CD_3_OD, 700 MHz) δ 4.50 (m, H-2), 3.73 (s, OCH_3_), 3.50 (m, H-15), 1.85 (m, H-3a), 1.68 (m, H-3b), 0.91 (t, *J* = 7.0 Hz, H-20); HRESIMS *m*/*z* 572.3563 [M–H]^−^ (calcd for C_31_H_49_F_3_NO_5_, 572.3569), *m*/*z* 608.3331 [M+Cl]^−^ (calcd for C_31_H_50_ClF_3_NO_5_, 608.3335), *m*/*z* 596.3559 [M+Na]^+^ (calcd for C_31_H_50_F_3_NO_5_Na, 596.3533).

*Preparation of (R)-MTPA amide of ***4c**. Pyridine (2.5 µL) and (*S*)-MTPA chloride (1.0 µL) were added to a solution of **4a** (0.7 mg) in CHCl_3_ (1.0 mL). After stirring at room temperature for 2 h, the reaction mixture was concentrated in vacuo.

*(R)-MTPA Amide* (**4c**): selected ^1^H NMR (CD_3_OD, 700 MHz) δ 4.45 (m, H-2), 3.70 (s, OCH_3_), 3.50 (m, H-15), 1.90 (m, H-3a), 1.80 (m, H-3b), 0.91 (t, *J* = 7.0 Hz, H-20); HRESIMS *m*/*z* 572.3567 [M–H]^−^ (calcd for C_31_H_49_F_3_NO_5_, 572.3569), *m*/*z* 608.3336 [M+Cl]^−^ (calcd for C_31_H_50_ClF_3_NO_5_, 608.3335), *m*/*z* 596.3561 [M+Na]^+^ (calcd for C_31_H_50_F_3_NO_5_Na, 596.3533).

*Preparation of MTPA esters of ***6a** *and ***6b**. A sample of **6** (0.5 mg) was treated with (*R*)-MTPACl (10 μL) and DMAP in dry pyridine (100 μL) in a 4 mL sealed vial. After stirring for 30 min at rt, the reaction mixtures were concentrated under reduced pressure. The residue was purified by HPLC (YMC-Pack ODS-A, 95% EtOH) to obtain **6a**. In the same way, (*R*)-MTPA ester **6b** was prepared from 0.5 mg of **6** by adding (*S*)-MTPA chloride and DMAP in dry pyridine.

*(S)-MTPA ester* (**6a**): selected ^1^H NMR (CD_3_OD, 700 MHz) δ 5.09 (1H, m, H-15), 1.64 (2H, m, H-16), 1.564 (2H, m, H-14), 1.32 (2H, m, H-18), 1.29 (2H, m, H-17), 1.18 (2H, m, H-13), 0.91 (3H, t, *J* = 7.0 Hz, H-19).

*(R)-MTPA ester* (**6b**): selected ^1^H NMR (CD_3_OD, 700 MHz) δ 5.09 (1H, m, H-15), 1.63 (2H, m, H-14), 1.575 (2H, m, H-16), 1.33 (2H, m, H-13), 1.247 (2H, m, H-18), 1.169 (2H, m, H-17), 0.84 (3H, t, *J* = 7.0 Hz, H-19).

*Preparation of MTPA esters of ***7a** *and ***7b**. A sample of **7** (0.5 mg) was treated with (*R*)-MTPACl (10 μL) and DMAP in dry pyridine (100 μL) in a 4 mL sealed vial. After stirring for 30 min at rt, the reaction mixtures were concentrated under reduced pressure. The residue was purified by HPLC (YMC-Pack ODS-A, EtOH) to obtain **7a**. In the same way, (*R*)-MTPA ester **7b** was prepared from 0.5 mg of **7** by adding (*S*)-MTPA chloride and DMAP in dry pyridine.

*(S)-MTPA ester* (**7a**): selected ^1^H NMR (CD_3_OD, 700 MHz) δ 5.10 (1H, m, H-15), 1.63 (2H, m, H-16), 1.57 (2H, H-14), 1.53 (1H, H-19), 1.33 (2H, m, H-17), 1.20 (2H, H-18), 0.876 (6H, d, *J* = 6.6 Hz, H-20, H-21).

*(R)-MTPA ester* (**7b**): selected ^1^H NMR (CD_3_OD, 700 MHz) δ 5.10 (1H, m, H-15), 1.64 (2H, H-14), 1.55 (2H, m, H-16), 1.44 (1H, m, H-19), 1.22 (2H, m, H-17), 1.14 (2H, m, H-18), 0.824 (6H, dd, *J* = 6.6, 1.3 Hz, H-20, H-21).

### 4.5. Bioassays

#### 4.5.1. Cell Cultures

Human neuroblastoma SH-SY5Y CRL-2266™ cells were obtained from the ATCC (Manassas, VA, USA). Rat cardiomyocytes (H9c2 cells) were transmitted from Prof. Dr. Gunhild von Amsberg from Martini-Klinik Prostate Cancer Center, University Hospital Hamburg-Eppendorf (Hamburg, Germany). JB6-Luc NF-*κ*B cell line was kindly provided by Dr. Zigang Dong, Hormel Institute (University of Minnesota, Minneapolis, MN, USA).

SH-SY5Y and H9c2 cells were cultured in Dulbecco’s Modified Eagle Medium (DMEM) from Biolot, St. Petersburg, Russia. Fetal bovine serum (10%) (Biolot, St. Petersburg, Russia) and penicillin/streptomycin mixture (1%) (Biolot, St. Petersburg, Russia) were added in DMEM.

The murine epidermal cell line JB6 P+ Cl41 and its stable transfectants JB6-Luc NF-*κ*B cells were cultured in minimal essential medium Eagle (MEM) with 5% fetal bovine serum, 2 mM L-glutamine, and penicillin/streptomycin (1%). Antibiotic geneticin G-418 was used to select transformed cells.

The cells were cultured at 37 °C in 5% (*v*/*v*) CO_2_.

#### 4.5.2. Cell Viability Assay

H9c2 and SH-SY5Y cells were seeded at a density of 3 × 10^3^ cells/well and 5 × 10^3^ cells/well, respectively. The experiments were initiated after 24 h. Compounds at concentrations of 1–100 µM were added to the cells for 48 h. All compounds were dissolved in DMSO such that the final concentration of DMSO in the cell culture medium was 1% or less. DMSO was used as a control.

The viability of the cells was measured using an MTT (3-(4,5-dimethylthiazol-2-yl)-2,5-diphenyltetrazolium bromide) assay, which was performed according to the manufacturer’s instructions (Sigma-Aldrich, Munich, Germany). The results are presented as percentages of the control data.

#### 4.5.3. In Vitro Acute Ischemia/Reperfusion (I/R) Modeling

H9c2 (3 × 10^3^ cells/well) and SH-SY (5 × 10^3^ cells/well) cells were seeded in 96-well plates and incubated overnight. The full culture medium was then removed, and poor medium (30% DMEM, 70% PBS) with 500 µM of CoCl_2_ was added for 5 h. The poor medium was replaced with a full culture medium. Thereafter, compounds **1**–**4** were added at a concentration of 1 µM. After 24 h, the viability of SH-SY5Y and H9c2 cells was measured using the MTT assay, as described above.

#### 4.5.4. In Vitro CoCl_2_-Mimic Hypoxia Modeling

The SH-SY5Y and H9c2 cells were treated with a dH_2_O solution of CoCl_2_ at a concentration of 500 µM for 1 h. Compounds were added at a concentration of 1 µM for 23 h (SH-SY5Y cells) or 47 h (H9c2 cells). The viability of SH-SY5Y and H9c2 cells was measured using the MTT assay, as described above.

#### 4.5.5. Reactive Oxygen Species Level Assay

The SH-SY5Y and H9c2 cells were treated with a dH_2_O solution of CoCl_2_ at a concentration of 500 µM for 1 h. Then the compounds at 1 µM were added for 3 h. Untreated cells were used as controls.

2,7-Dichlorodihydrofluorescein diacetate solution (H_2_DCFDA, Molecular Probes, Eugene, OR, USA) at 10 μM was added to each well, and the plate was incubated for an additional 10 min at 37 °C. Then the cells were washed with phosphate saline buffer twice. The intensity of the dichlorofluorescein fluorescence (*λ*_ex_ = 485 nm, *λ*_em_ = 518 nm) was measured using a PHERAstar FS plate reader (BMG Labtech, Ortenberg, Germany). The data were processed using MARS Data Analysis v. 3.01R2 (BMG Labtech, Ortenberg, Germany). The results are presented as relative fluorescence units.

#### 4.5.6. Superoxide Dismutase Activity Measurement

SH-SY5Y and H9c2 cells were seeded in 6-well plates, and experiments were started after 24 h. The dH_2_O solution of CoCl_2_ at a concentration of 500 µM was added for 1 h, and then compounds at 1 µM were added for 2 or 3 h. Untreated cells were used as controls.

The cells were washed twice with PBS, collected, and lysed with RIPA buffer (Sigma-Aldrich, St. Louis, MO, USA). The cell lysates were then centrifuged at 14,000 rpm (Eppendorf, Framingham, MA, USA), and the supernatant was used to detect SOD activity as described [45].

#### 4.5.7. Determination of the Effect of the Compounds on the Basal Transcriptional Activity of NF-*κ*B Nuclear Factor

The experiment was carried out as described in [46]. In brief, the JB6 Cl41 NF-*κ*B cells (8 × 10^3^ cells/well) were seeded into 96-well plate overnight, and then the compounds at a concentration of 1 µM were added for 3, 6 or 24 h.

Then, the cells were disrupted for 1 h at RT with lysis buffer (0.1M potassium phosphate buffer at pH 7.8, 1% Triton X-100, 1 mM DTT, 2 mM EDTA) and 30 µL of lysate from each well were transferred into a plate for luminescent analysis and luciferase activity was measured using luciferase assay buffer (100 µL/well) containing 0.47 mM D-luciferin, 20 mM Tricin, 1.07 mM (MgCO_3_)_4_ × Mg(OH)_2_ × 5H_2_O, 2.67 mM MgSO_4_ × 7H_2_O, 33.3 mM DTT, 0.53 mM ATP, 0.27 mM CoA, and 0.1 mM EDTA, pH 7.8. The measurements were performed using a Luminoscan Ascent Type 392 microplate reader (Labsystems, Helsinki, Finland). The results are expressed as luminescence intensity.

#### 4.5.8. ATP Toxicity Assay

The SH-SY5Y and H9c2 cells were treated with compounds at a concentration of 1 µM for 1 h, and then a dH_2_O solution of ATP at a concentration of 4 mM was added for 24 h. Brilliant blue G-250 was used as positive control. The viability of the SH-SY5Y and H9c2 cells was measured by an MTT assay as described above.

#### 4.5.9. Ca^2+^ Influx Measurements

H9c2 (6 × 10^3^ cells/well) or SH-SY5Y (1 × 10^4^ cells/well) cells were seeded in 96-well plates and incubated overnight. The cells were then washed once with HBSS saline (pH 7.4) and loaded with 4 μM Fluo-8 AM (Abcam, Cambridge, UK) and 0.05% (*w*/*v*) Pluoronic^®^ F-127 (Sigma-Aldrich, St. Louis, MO, USA) in HBSS saline buffer. After incubation for 40 min, the cells were washed with HBSS and treated with the compounds for 30 min at RT in the dark. P2X7R antagonist A438079 at 10 μM (Sigma Aldrich, St. Louis, MO, USA) was used as a positive control.

The timeline of the fluorescence intensity (*λ*_ex_ = 480 nm, *λ*_em_ = 520 nm) changing was measured using a PHERAstar FS plate reader (BMG LABTECH, Ortenberg, Germany) during 70 s. ATP at 2 mM was added using a robotic microinjector after baseline recording. The data were processed by MARS Data Analysis v. 3.01R2 (BMG Labtech, Ortenberg, Germany).

### 4.6. Statistical Data Evaluation

All data were obtained in three independent repeats, and the calculated values were expressed as the mean ± the standard error of the mean (SEM). To determine the statistical significance, Student’s *t*-test was performed using SigmaPlot 14.0 (Systat Software Inc., San Jose, CA, USA). The differences were considered statistically significant at *p* < 0.05, unless otherwise indicated.

## 5. Conclusions

Thus, anthonoic acids A–C (**1**–**3**) and anthamino acid A (**4**), obtained from the Northern Pacific marine sponge *A. ridgwayi,* are the first representatives of bipolar natural nonproteinogenic amino acids with sulfated polymethylene chains. The *N*-2-hydroxyethyl residue has also not been previously detected in lipoamino acids.

Anthonoic acids A–C (**1**–**3**) may protect cardiomyocytes H9c2 and neuroblastoma SH-SY5Y cells against ischemia/reperfusion and chronic hypoxia. Based on the results of our experiments, we suggest that their action led to the reduction in induced damage, at least in part, via the Nrf2-mediated and P2X7 receptor-dependent pathways.

## Figures and Tables

**Table 1:** ^1^H (700 MHz), ^13^C (175 MHz), and ^15^N (71 MHz) NMR Spectroscopic Data for **1**, **2**, and **3** in CD_3_OD.

Position	1		2		3	
	*δ*_C_*^a^*, Type	*δ*_H_ Mult (*J* in Hz)	*δ*_C_*^a^*, Type	*δ*_H_ Mult (*J* in Hz)	*δ*_C_*^a^*, Type	*δ*_H_ Mult (*J* in Hz)
1	173.0, C		173.4, C		173.8, C	
2	63.1, CH	3.68, t (5.8)	63.9, CH	3.53, t (5.9)	63.8, CH	3.56, m
3	31.2, CH_2_	1.89, m	31.6, CH_2_	1.86, m	31.5, CH_2_	1.87, m
4a	26.04, CH_2_	1.49, m	26.2, CH_2_	1.46, m	26.2, CH_2_	1.47, m
4b		1.42, m				
5	30.5, CH_2_	1.36, m	30.5, CH_2_	1.36, m	30.4, CH_2_	1.36, m
6	30.6, CH_2_	1.30, m	30.6, CH_2_	1.30, m	30.5, CH_2_	1.30, m
7	30.6, CH_2_	1.30, m	30.6, CH_2_	1.30, m	30.5, CH_2_	1.30, m
8	30.6, CH_2_	1.30, m	30.6, CH_2_	1.30, m	30.5, CH_2_	1.30, m
9	30.6, CH_2_	1.30, m	30.6, CH_2_	1.30, m	30.5, CH_2_	1.30, m
10	30.6, CH_2_	1.30, m	30.6, CH_2_	1.30, m	30.5, CH_2_	1.30, m
11	30.6, CH_2_	1.30, m	30.6, CH_2_	1.30, m	30.5, CH_2_	1.30, m
12	30.7, CH_2_	1.30, m	30.7, CH_2_	1.30, m	30.5, CH_2_	1.34, m
13	26.00, CH_2_	1.40, m	26.0, CH_2_	1.40, m	26.0, CH_2_	1.40, m
14	35.3, CH_2_	1.61, m	35.4, CH_2_	1.63, m	35.3, CH_2_	1.64, m
15	80.9, CH	4.33, quint (6.0)	80.9, CH	4.33, quint (5.9)	81.0, CH	4.33, quint (6.0)
16	35.3, CH_2_	1.65, m	35.1, CH_2_	1.63, m	35.6, CH_2_	1.61, m
17	25.7, CH_2_	1.40, m	28.3, CH_2_	1.38, m	23.9, CH_2_	1.40, m
18	33.1, CH_2_	1.30, m	23.8, CH_2_	1.34, m	40.2, CH_2_	1.19, m
						1.22, m
19	23.7, CH_2_	1.33, m	14.4, CH_3_	0.92, t (7.0)	29.1	1.55, m
20	14.4, CH_3_	0.91, t (7.0)	-	-	23.0	0.89, d (6.7)
21	-	-	-	-	23.0	0.89, d (6.7)
1′	49.9, CH_2_	3.14, m	50.0, CH_2_	3.10, m	50.0	3.12, m
2′	58.0, CH_2_	3.80, t (5.3)	58.1, CH_2_	3.79, t (5.4)	58.1	3.78, t (5.3)
N (*δ*_N_ 46.6) *^b^*						

*^a^* ^13^C NMR assignments supported by HSQC and HMBC data. *^b^ δ*_N_ derived from the ^1^H-^15^N HMBC data.

## Data Availability

All relevant data are included in the manuscript or in the Supplementary Materials.

## Supplementary Materials

The following supporting information can be downloaded at https://www.mdpi.com/article/10.3390/molecules31010036/s1, Figure S1: ^1^H NMR spectrum of anthonoic acid A (**1**) in CD_3_OD (700 MHz); Figure S2: Partial of the ^1^H NMR spectrum of anthonoic acid A (**1**) in CD_3_OD (700 MHz); Figure S3: ^13^C NMR spectrum of anthonoic acid A (**1**) in CD_3_OD (175 MHz); Figure S4: Partial of the ^13^C NMR spectrum of anthonoic acid A (**1**) in CD_3_OD (175 MHz); Figure S5: ^1^H-^1^H COSY spectrum of anthonoic acid A (**1**) in CD_3_OD; Figure S6: HSQC spectrum of anthonoic acid A (**1**) in CD_3_OD; Figure S7: HMBC spectrum of anthonoic acid A (**1**) in CD_3_OD; Figure S8: ^1^H-^15^N HMBC spectrum of anthonoic acid A (**1**) in CD_3_OD; Figure S9: HRESIMS spectrum of anthonoic acid A (**1**); Figure S10: (–)ESIMS/MS spectrum of [M_Na_–Na]^−^ precursor ion at m/z 466 of anthonoic acid A (**1**); Figure S11: Fragmentation of **1** in (–)HRESIMS/MS; Table S1: MS^2^ spectra of anthonoic acid A (**1**) obtained under electrospray ionization in the negative ion detection mode; Figure S12: ^1^H NMR spectrum of anthonoic acid B (**2**) in CD_3_OD (700 MHz); Figure S13: Partial of the ^1^H NMR spectrum of anthonoic acid B (**2**) in CD_3_OD (700 MHz); Figure S14: ^13^C NMR spectrum of anthonoic acid B (**2**) in CD_3_OD (175 MHz); Figure S15: Partial of the ^13^C NMR spectrum of anthonoic acid B (**2**) in CD_3_OD (175 MHz); Figure S16: ^1^H-^1^H COSY spectrum of anthonoic acid B (**2**) in CD_3_OD; Figure S17: HSQC spectrum of anthonoic acid B (**2**) in CD_3_OD; Figure S18: HMBC spectrum of anthonoic acid B (**2**) in CD_3_OD; Figure S19: HRESIMS spectrum of anthonoic acid B (**2**); Figure S20: (–)ESIMS/MS spectrum of [M_Na_–Na]^−^ precursor ion at m/z 452 of anthonoic acid B (**2**); Figure S21: Fragmentation of **2** in (–)HRESIMS/MS; Table S2: MS^2^ spectra of anthonoic acid B (**2**) obtained under electrospray ionization in the negative ion detection mode; Figure S22: ^1^H NMR spectrum of anthonoic acid C (**3**) in CD_3_OD (700 MHz); Figure S23: Partial of the ^1^H NMR spectrum of anthonoic acid C (**3**) in CD_3_OD (700 MHz); Figure S24: ^13^C NMR spectrum of anthonoic acid C (**3**) in CD_3_OD (175 MHz); Figure S25. Partial of the ^13^C NMR spectrum of anthonoic acid C (**3**) in CD_3_OD (175 MHz); Figure S26: ^1^H-^1^H COSY spectrum of anthonoic acid C (**3**) in CD_3_OD; Figure S27: HSQC spectrum of anthonoic acid C (**3**) in CD_3_OD; Figure S28: HMBC spectrum of anthonoic acid A (**1**) in CD_3_OD; Figure S29: HRESIMS spectrum of anthonoic acid C (**3**); Figure S30: (–)ESIMS/MS spectrum of [M_Na_–Na]^−^ precursor ion at m/z 480 of anthonoic acid C (**3**); Figure S31: Fragmentation of **3** in (–)HRESIMS/MS; Table S3: MS^2^ spectra of anthonoic acid C (**3**) obtained under electrospray ionization in the negative ion detection mode; Figure S32: ^1^H NMR spectrum of mixture of anthamino acid A (**4**) and **1** in CD_3_OD (700 MHz); Figure S33: ^13^C NMR spectrum of mixture of anthamino acid A (**4**) and **1** in CD_3_OD (175 MHz); Figure S34: HRESIMS spectrum of anthamino acid A (**4**); Figure S35: ^1^H NMR spectrum of (S)-MTPA amide (**4b**) in CD_3_OD (700 MHz); Figure S36: ^1^H NMR spectrum of (R)-MTPA amide (**4c**) in CD_3_OD (700 MHz); Figure S37: ^1^H NMR spectrum of (S)-MTPA ester **6a** in CD_3_OD (700 MHz); Figure S38: ^1^H NMR spectrum of (R)-MTPA ester **6b** in CD_3_OD (700 MHz); Figure S39: 1D selective TOCSY spectrum of (S)-MTPA ester **6a** with selective excitation of H_3_-19 (700 MHz, CD_3_OD); Figure S40: 1D selective TOCSY spectrum of (R)-MTPA ester **6b** with selective excitation of H_3_-19 (700 MHz, CD_3_OD); Figure S41: ^1^H NMR spectrum of (S)-MTPA ester **7a** in CD_3_OD (700 MHz); Figure S42: ^1^H NMR spectrum of (R)-MTPA ester **7b** in CD_3_OD (700 MHz); Figure S43: 1D selective TOCSY spectrum of (S)-MTPA ester **7a** with selective excitation of H_3_-19 (700 MHz, CD_3_OD); Figure S44: 1D selective TOCSY spectrum of (R)-MTPA ester **7b** with selective excitation of H_3_-19 (700 MHz, CD_3_OD); Figure S45: Images of the sample and spicules of the sponge Antho (Acarnia) ridgwayi Stone, Lehnert & Hoff, 2019 (order Poecilosclerida, family Microcionidae, the registration number PIBOC O47-142).
