# Supplementary material for: Anthonoic Acids A–C, Sulfated and N-(2-Hydroxyethyl)-Substituted Lipidic Amino Acids from the Marine Sponge Antho ridgwayi with In Vitro Cytoprotective Activities
Source: Molecules. 2025 Dec 22;31(1):36. doi: 10.3390/molecules31010036 (PMC12787137; doi:10.3390/molecules31010036)
Supplement: Supplementary file 1 [file molecules-31-00036-s001.zip › molecules-4051723-supplementary.pdf]

Supporting Information

Anthonoic Acids A-C, Sulfated and *N*-(2-hydroxyethyl)-substituted Lipidic amino acids, from the Marine Sponge *Antho ridgwayi* with in vitro cytoprotective activities

Alla G. Guzii,[†] *Ekaterina K. Kudryashova,*[†] *Larisa K. Shubina,*[†] *Tatyana N. Makarieva,*[*,†] *Alexander S. Menshov,*[†] *Roman S. Popov,*[†] *Ekaterina A. Yurchenko,*[*,†] *Evgeny A. Pislyagin,*[†] *Ekaterina A. Chingizova,*[†] *Boris B. Grebnev,*[†] *Vladimir A. Shilov,*[‡] *and Valentin A. Stonik*[†]

[†] G. B. Elyakov Pacific Institute of Bioorganic Chemistry, Far Eastern Branch of the Russian Academy of Sciences, Prospect 100-let Vladivostoku 159, Vladivostok 690022, Russian Federation

[‡] A. V. Zhirmunsky National Scientific Center of Marine Biology, Far Eastern Branch of the Russian Academy of Sciences, 17 Palchevskogo Str., Vladivostok 690041, Russian Federation

Content

Previously reported lipid α-amino acids 3

Figure S1. ^{1}H NMR spectrum of anthonoic acid A (1) in CD_3OD (700 MHz). 5

Figure S2. Partial of the ^{1}H NMR spectrum of anthonoic acid A (1) in CD_3OD (700 MHz).........6

Figure S3. ^{13}C NMR spectrum of anthonoic acid A (1) in CD_3OD (175 MHz). 7

Figure S4. Partial of the ^{13}C NMR spectrum of anthonoic acid A (1) in CD_3OD (175 MHz)........8

Figure S5. ^{1}H-^{1}H COSY spectrum of anthonoic acid A (1) in CD_3OD. 9

Figure S6. HSQC spectrum of anthonoic acid A (1) in CD_3OD. 10

Figure S7. HMBC spectrum of anthonoic acid A (1) in CD_3OD. 11

Figure S8. ^{1}H-^{15}N HMBC spectrum of anthonoic acid A (1) in CD_3OD. 12

Figure S9. HRESIMS spectrum of anthonoic acid A (1). 13

Figure S10. (–)ESIMS/MS spectrum of $[M_{Na} – Na]^{–}$ precursor ion at *m/z* 466 of anthonoic acid A (1). 13

Figure S11. Fragmentation of 1 in (–)HRESIMS/MS. 14

Table S1. MS2 spectra of anthonoic acid A (1) obtained under electrospray ionization in the negative ion detection mode. 14

Figure S12. ^{1}H NMR spectrum of anthonoic acid B (2) in CD_3OD (700 MHz)..15

Figure S13. Partial of the ^{1}H NMR spectrum of anthonoic acid B (2) in CD_3OD (700 MHz). 16

Figure S14. ^{13}C NMR spectrum of anthonoic acid B (2) in CD_3OD (175 MHz)..17

Figure S15. Partial of the ^{13}C NMR spectrum of anthonoic acid B (2) in CD_3OD (175 MHz). ...18

Figure S16. ^{1}H-^{1}H COSY spectrum of anthonoic acid B (2) in CD_3OD..19

Figure S17. HSQC spectrum of anthonoic acid B (2) in CD_3OD. 20

Figure S18. HMBC spectrum of anthonoic acid B (2) in CD_3OD. 21

Figure S19. HRESIMS spectrum of anthonoic acid B (2). 22
Figure S20. (–)ESIMS/MS spectrum of [M_{Na} – Na]$^-$ precursor ion at *m/z* 452 of anthonoic acid B (2). 22
Figure S21. Fragmentation of 2 in (–)HRESIMS/MS. 23
Table S2. MS^2 spectra of anthonoic acid B (2) obtained under electrospray ionization in the negative ion detection mode. 23
Figure S22. ^{1}H NMR spectrum of anthonoic acid C (3) in CD_3OD (700 MHz). 24
Figure S23. Partial of the ^{1}H NMR spectrum of anthonoic acid C (3) in CD_3OD (700 MHz). 25
Figure S24. ^{13}C NMR spectrum of anthonoic acid C (3) in CD_3OD (175 MHz). 26
Figure 25. Partial of the ^{13}C NMR spectrum of anthonoic acid C (3) in CD_3OD (175 MHz). 27
Figure S26. ^{1}H-^{1}H COSY spectrum of anthonoic acid C (3) in CD_3OD. 28
Figure S27. HSQC spectrum of anthonoic acid C (3) in CD_3OD. 29
Figure S28. HMBC spectrum of anthonoic acid C (3) in CD_3OD. 30
Figure S29. HRESIMS spectrum of anthonoic acid C (3). 31
Figure S30. (–)ESIMS/MS spectrum of [M_{Na} – Na]$^-$ precursor ion at *m/z* 480 of anthonoic acid C (3). 31
Figure S31. Fragmentation of 3 in (–)HRESIMS/MS 32
Table S3. MS^2 spectra of anthonoic acid C (3) obtained under electrospray ionization in the negative ion detection mode. 32
Figure S32. ^{1}H NMR spectrum of mixture of anthamino acid A (4) and 1 in CD_3OD (700 MHz). 33
Figure S33. ^{13}C NMR spectrum of mixture of anthamino acid A (4) and 1 in CD_3OD (175 MHz). 34
Figure S34. HRESIMS spectrum of anthamino acid A (4). 35
Figure S35. ^{1}H NMR spectrum of (*S*)-MTPA amide (4b) in CD_3OD (700 MHz). 36
Figure S36. ^{1}H NMR spectrum of (*R*)-MTPA amide (4c) in CD_3OD (700 MHz). 36
Figure S37. ^{1}H NMR spectrum of (*S*)-MTPA ester 6a in CD_3OD (700 MHz). 37
Figure S38. ^{1}H NMR spectrum of (*R*)-MTPA ester 6b in CD_3OD (700 MHz). 37
Figure S39. 1D selective TOCSY spectrum of (*S*)-MTPA ester 6a with selective excitation of H_3-19 (700 MHz, CD_3OD). 38
Figure S40. 1D selective TOCSY spectrum of (*R*)-MTPA ester 6b with selective excitation of H_3-19 (700 MHz, CD_3OD). 38
Figure S41. ^{1}H NMR spectrum of (*S*)-MTPA ester 7a in CD_3OD (700 MHz). 39
Figure S42. ^{1}H NMR spectrum of (*R*)-MTPA ester 7b in CD_3OD (700 MHz). 39
Figure S43. 1D selective TOCSY spectrum of (*S*)-MTPA ester 7a with selective excitation of H_3-19 (700 MHz, CD_3OD). 40
Figure S44. 1D selective TOCSY spectrum of (R)-MTPA ester 7b with selective excitation of H3-19 (700 MHz, CD_3OD). 40
Figure S45. Images of the sample and spicules of the sponge *Antho (Acarnia) ridgwayi* Stone, Lehnert & Hoff, 2019 (order Poecilosclerida, family Microcionidae, the registration number PIBOC O47-142). 41

Previously reported lipid α-amino acids

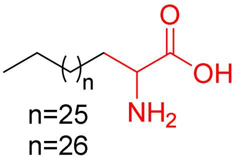

lipidic α-amino acids
from *Protopalythoa variabilis*.[1]

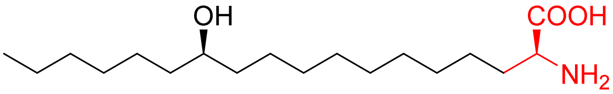

Simplifungin[2]

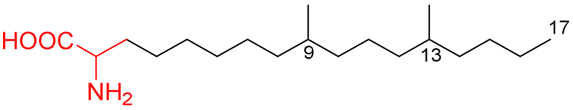

2-Amino-9,13-dimethyl heptadecanoic acid[3]

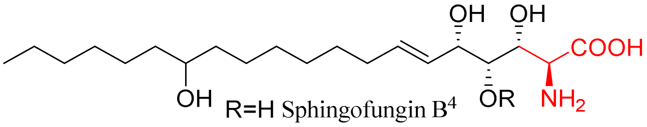

R=H Sphingofungin B[4]
R=Ac Fumifungin[5]

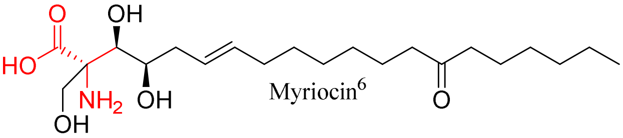

Myriocin[6]

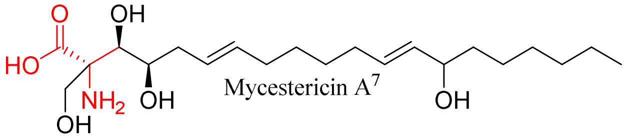

Mycestericin A[7]

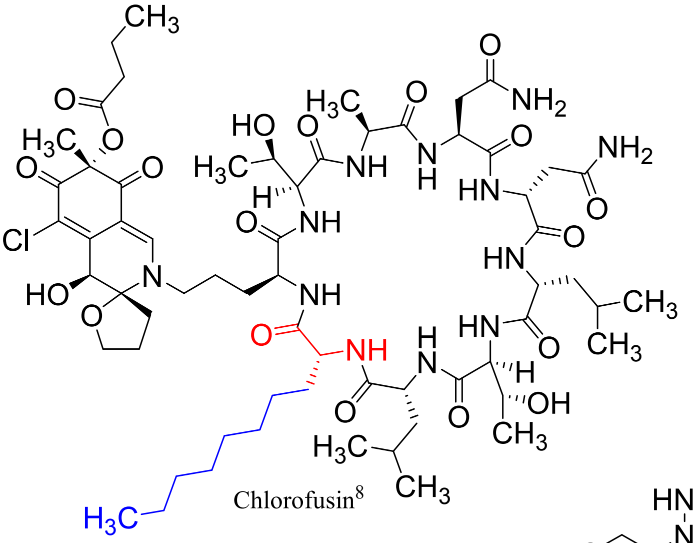

Chlorofusin[8]

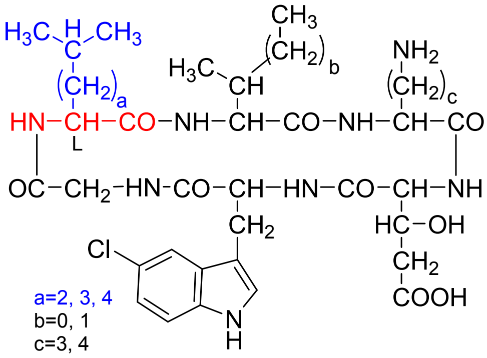

Longicatenamycin[9]

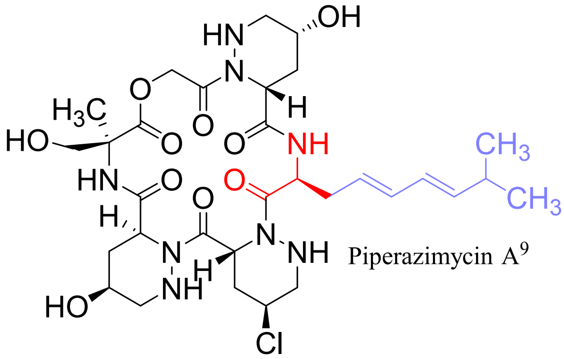

Piperazimycin A[9]

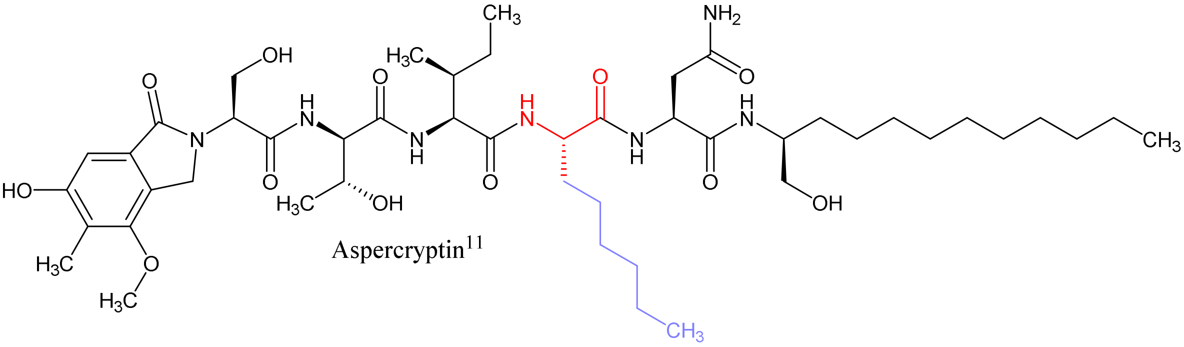

Aspercryptin[11]

REFERENCES

1. Wilke, D. V.; Jimenez, P. C.; Pessoa, C.; De Moraes, M. O.; Araujo, R. M.; Da Silva, W. M. B.; Silveira, E. R.; Pessoa, O. D. L.; Braz-Filho, R.; Lopes, N. P.; Costa-Lotufo, L. V. *J. Braz. Chem. Soc.* **2009**, *20*, 1455−1459.
2. Ishijima, H.; Uchida, R.; Ohtawa, M.; Kondo, A.; Nagai, K.; Shima, K.; Nonaka, K.; Masuma, R.; Iwamoto, S.; Onodera, H.; Nagamitsu, T.; Tomoda, H. *J. Org. Chem.* **2016**, *81*, 7373−7383.
3. Ivanova, V.; Oriol, M.; Montes, M. J.; Garcia, A.; Guinea, J. *Z. Nsturforsch. C. Biosci.* **2001**, *56*, 1−5.
4. VanMiddlesworth, F.; Giacobbe, R. A.; Lopez, M.; Garrity, G.; Bland, J. A.; Bartizal, K.; Fromtling, R. A.; Polishook, J.; Zweerink, M.; Edison, A. M.; Rozdilsky, W.; Wilson, K. E. *J. Antibiot.* **1992**, *45*, 861−867.
5. Mukhopadhyay, T.; Roy, K.; Coutinho, L.; Rupp, R. H.; Ganguli, B. N.; Fehlhaber, H. W. *J. Antibiot.* 1987, *40*, 1050−1052.
6. Kluepfel, D.; Bagli, J.; Baker, H.; Charest, M. P.; Kudelski, A.; Sehgal, S. N.; Vezina, C. *J. Antibiot.* 1972, *25*, 109−115.
7. Sasaki, S.; Ikumoto, T.; Hirose, R.; Kiuchi, M.; Fujita, T.; Hashimoto, R.; Inoue, K.; Chiba, K.; Hoshino, Y.; Okumoto, T. *J. Antibiot*. **1994**, *47*, 420−433.
8. Duncan, S. J.; Gru1schow, S.; Williams, D. H.; McNicholas, C.; Purewal, R.; Hajek, M.; Gerlitz, M.; Martin, S.; Wrigley, S. K.; Moore, M. *J. Am. Chem. Soc.* **2001**, *123*, 554−560.
9. Miller, E. D.; Kauffman, C. A.; Jensen, P. R.; Fenical, W. *J. Org. Chem.* **2007**, *72*, 323−330.
10. Shiba, T.; Mukunoki, Y. *J. Antibot.* **1975**, *28*, 561−566.
11. Henke, M. T.; Soukup, A. A.; Goering, A. W.; McClure, R. A.; Thomson, R. J.; Keller, N. P.; Kelleher, N. L. *ACS Chem. Biol.* **2016**, *11*, 2117−2123.

Figure S1. ^{1}H NMR spectrum of anthonoic acid A (**1**) in CD_3OD (700 MHz).

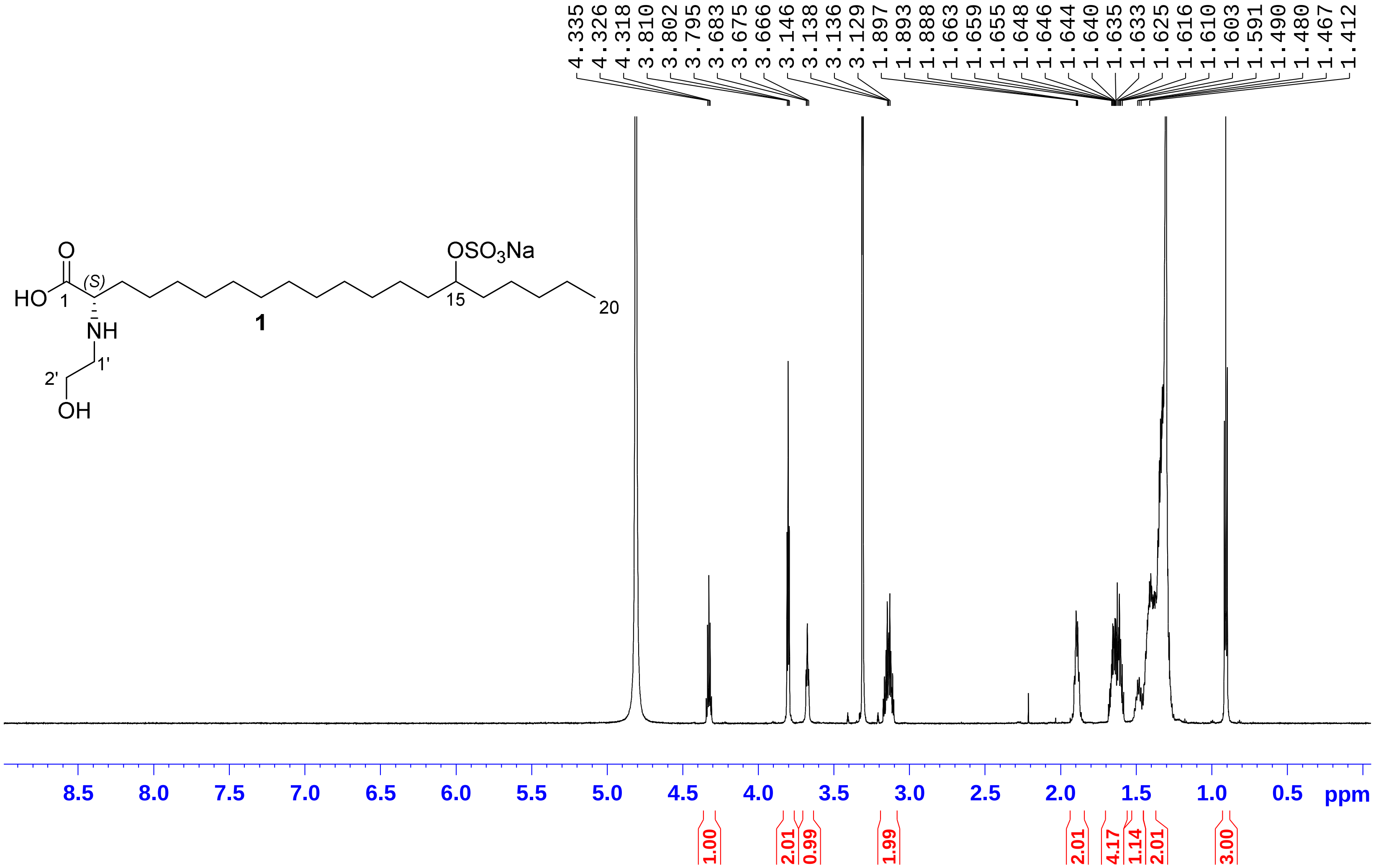

Figure S2. Partial of the ^{1}H NMR spectrum of anthonoic acid A (**1**) in CD_3OD (700 MHz).

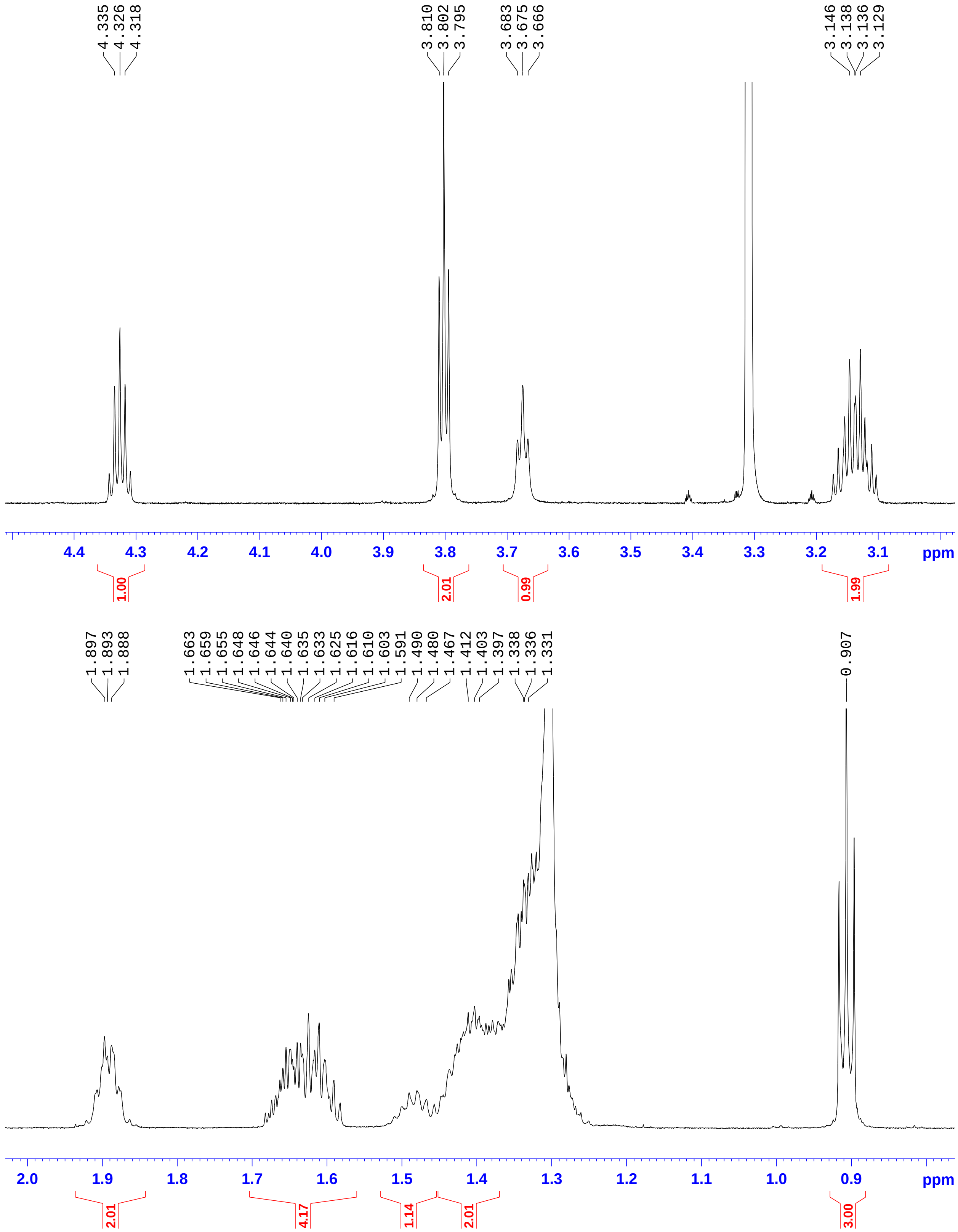

Figure S3. ^{13}C NMR spectrum of anthonoic acid A (**1**) in CD_3OD (175 MHz).

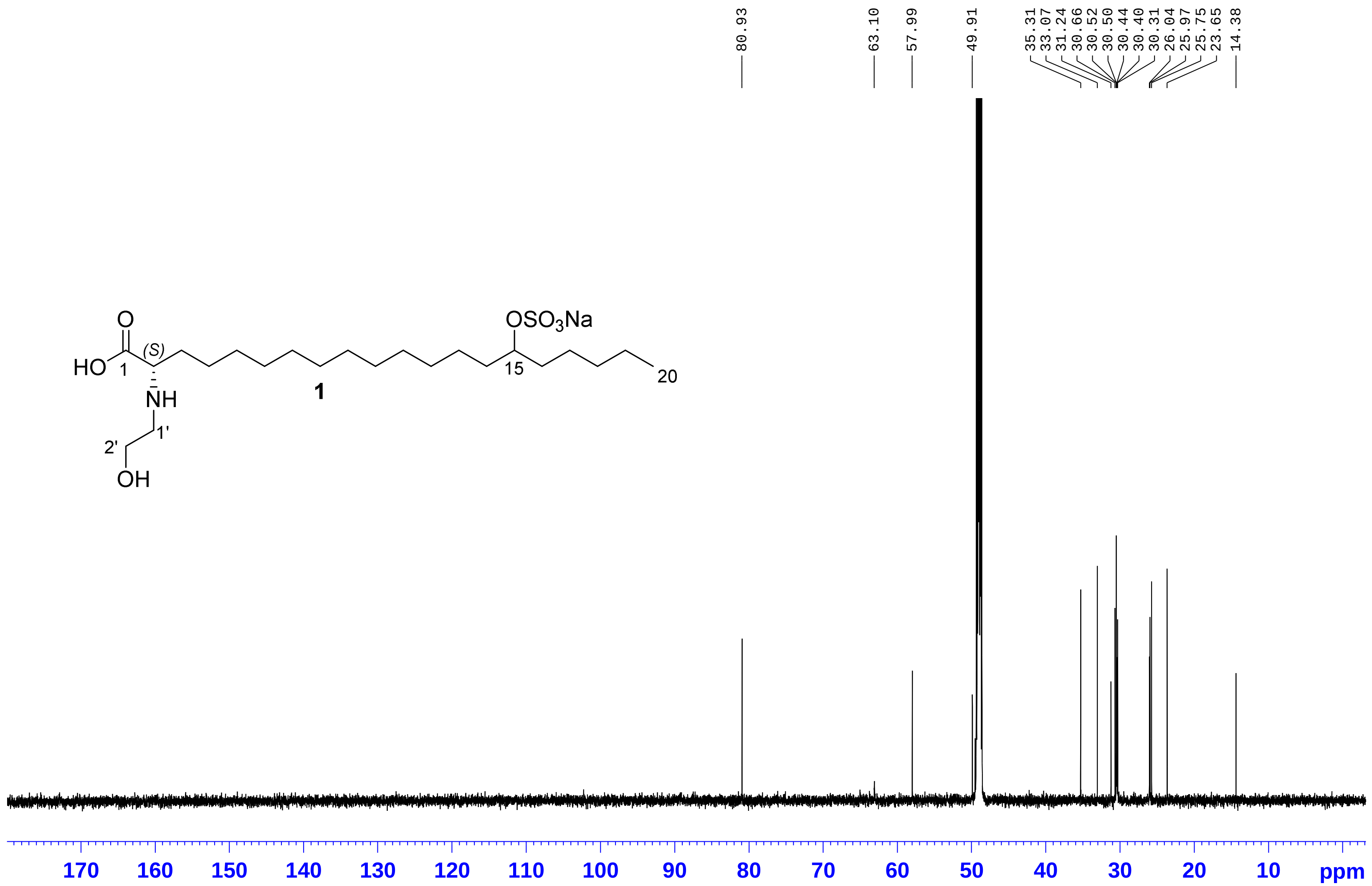

Figure S4. Partial of the ^{13}C NMR spectrum of anthonoic acid A (**1**) in CD_3OD (175 MHz).

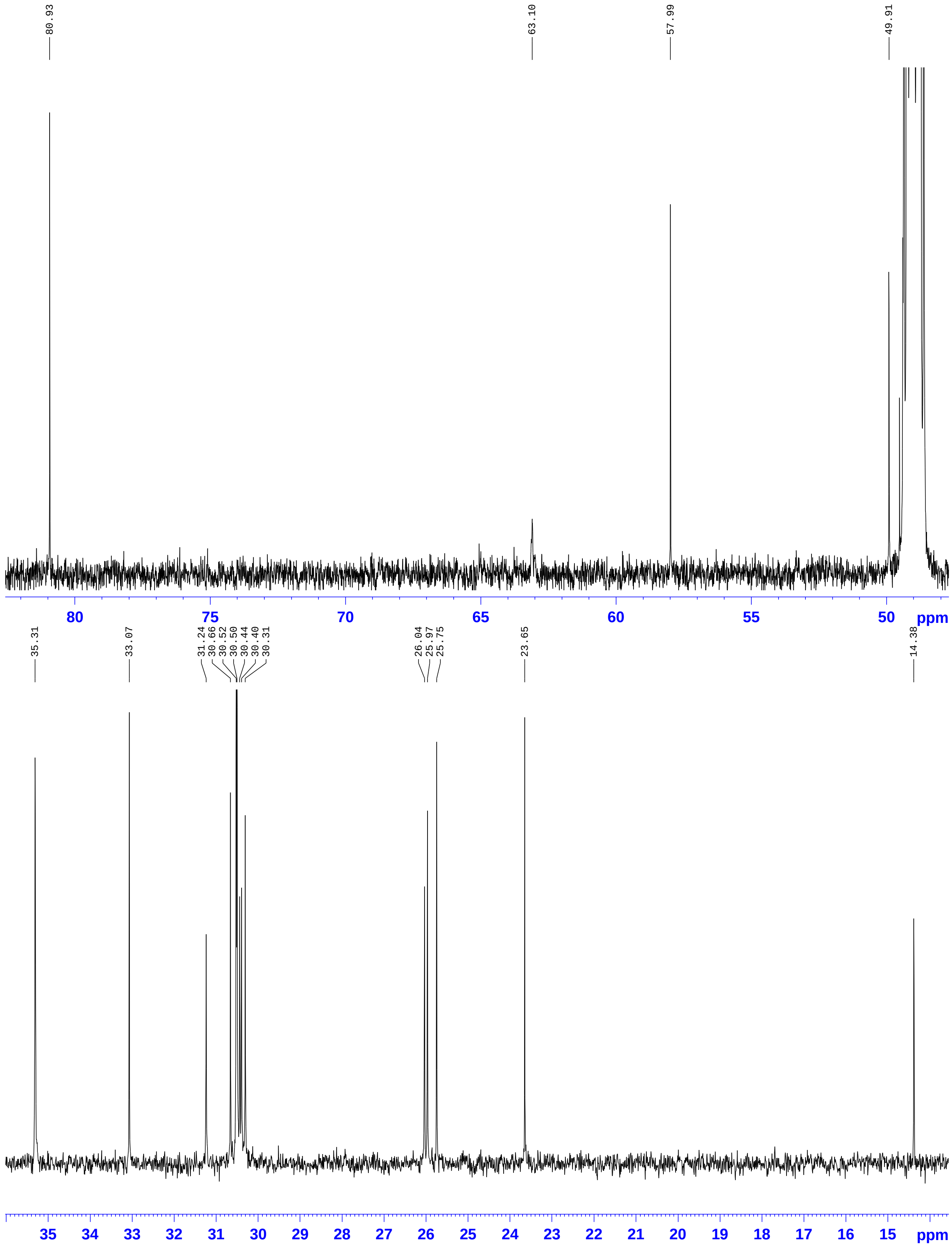

Figure S5. ^{1}H-^{1}H COSY spectrum of anthonoic acid A (**1**) in CD_3OD.

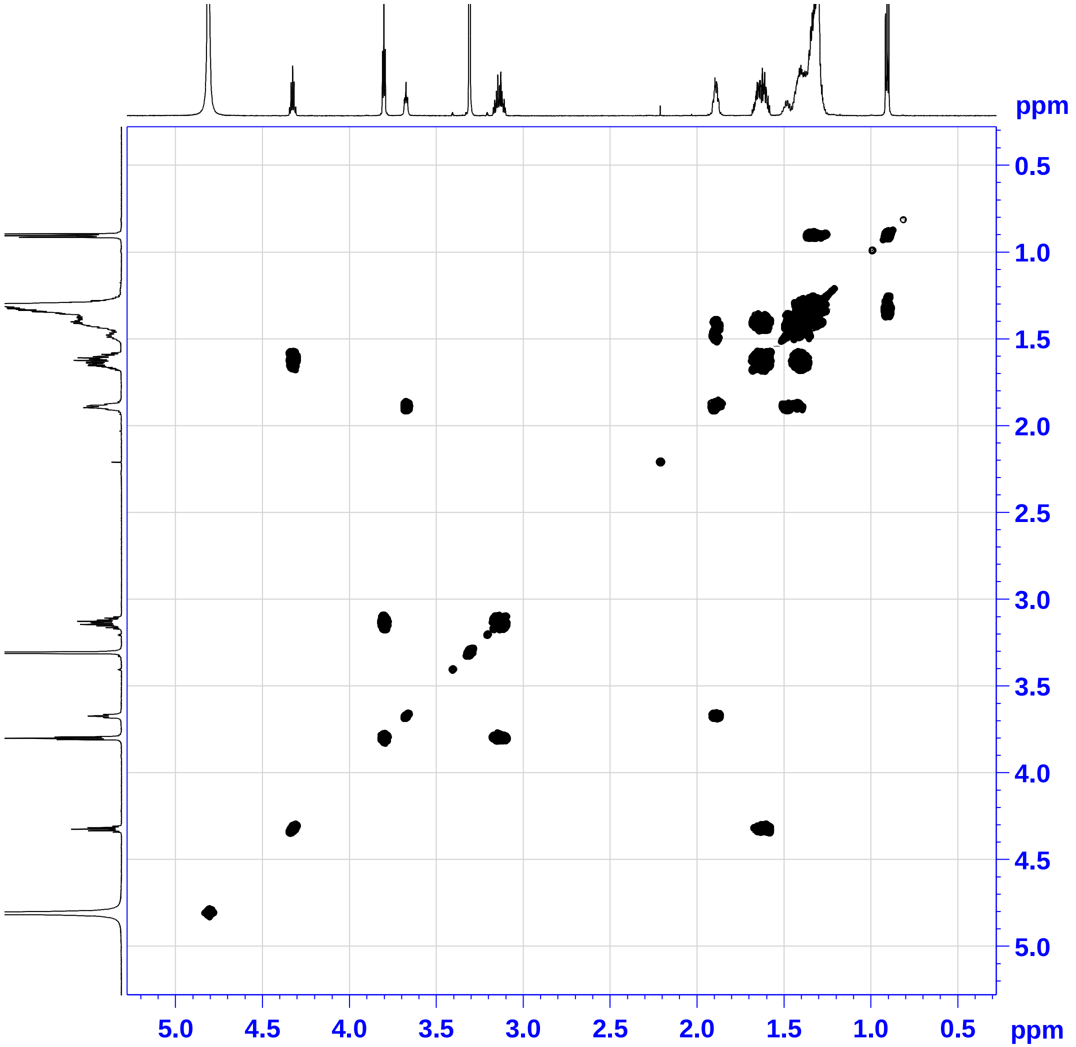

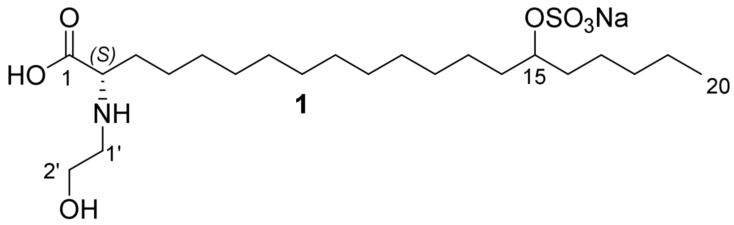

1

Figure S6. HSQC spectrum of anthonoic acid A (**1**) in CD_3OD.

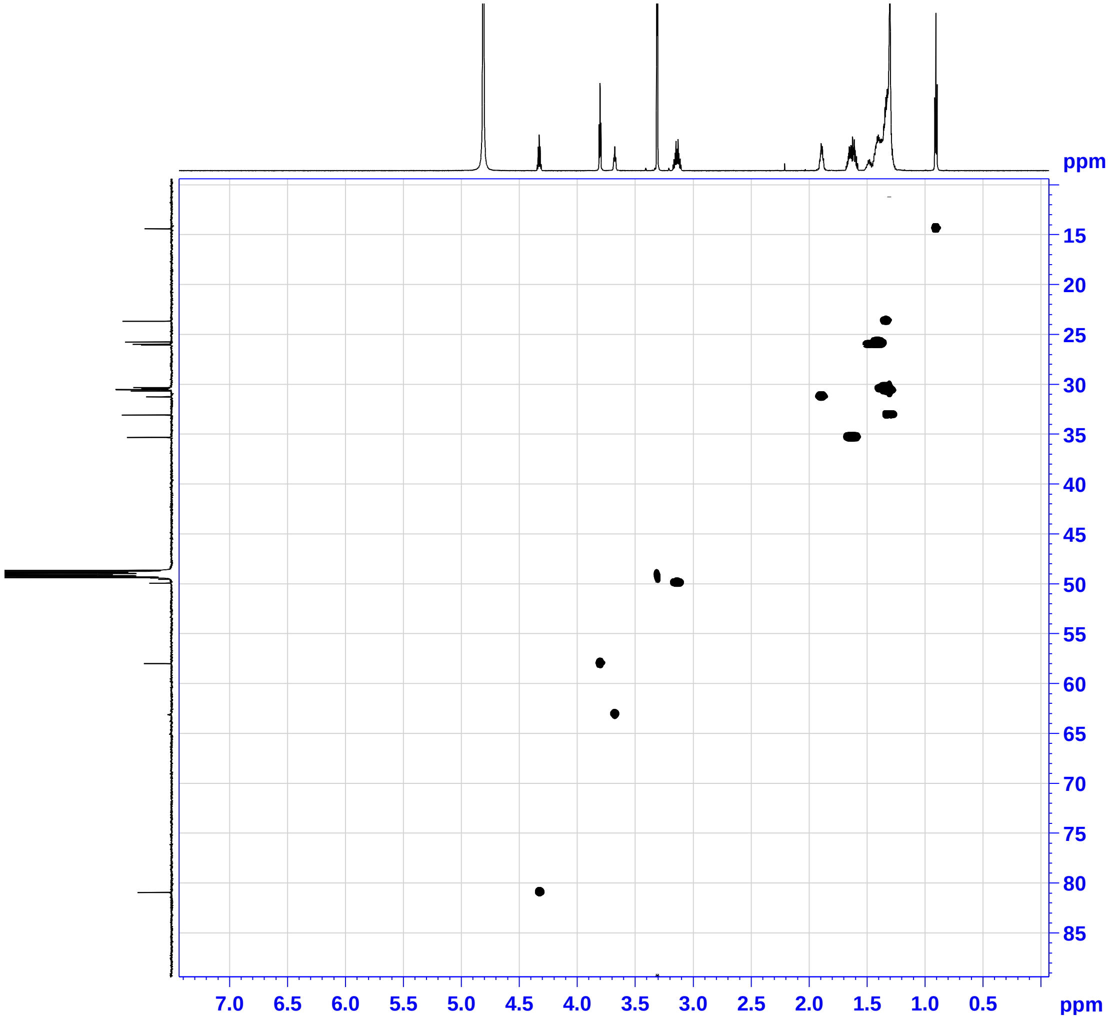

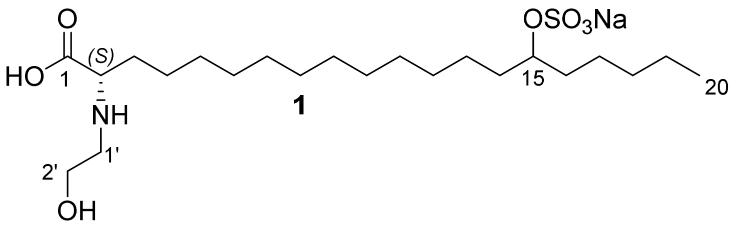

Figure S7. HMBC spectrum of anthonoic acid A (**1**) in CD_3OD.

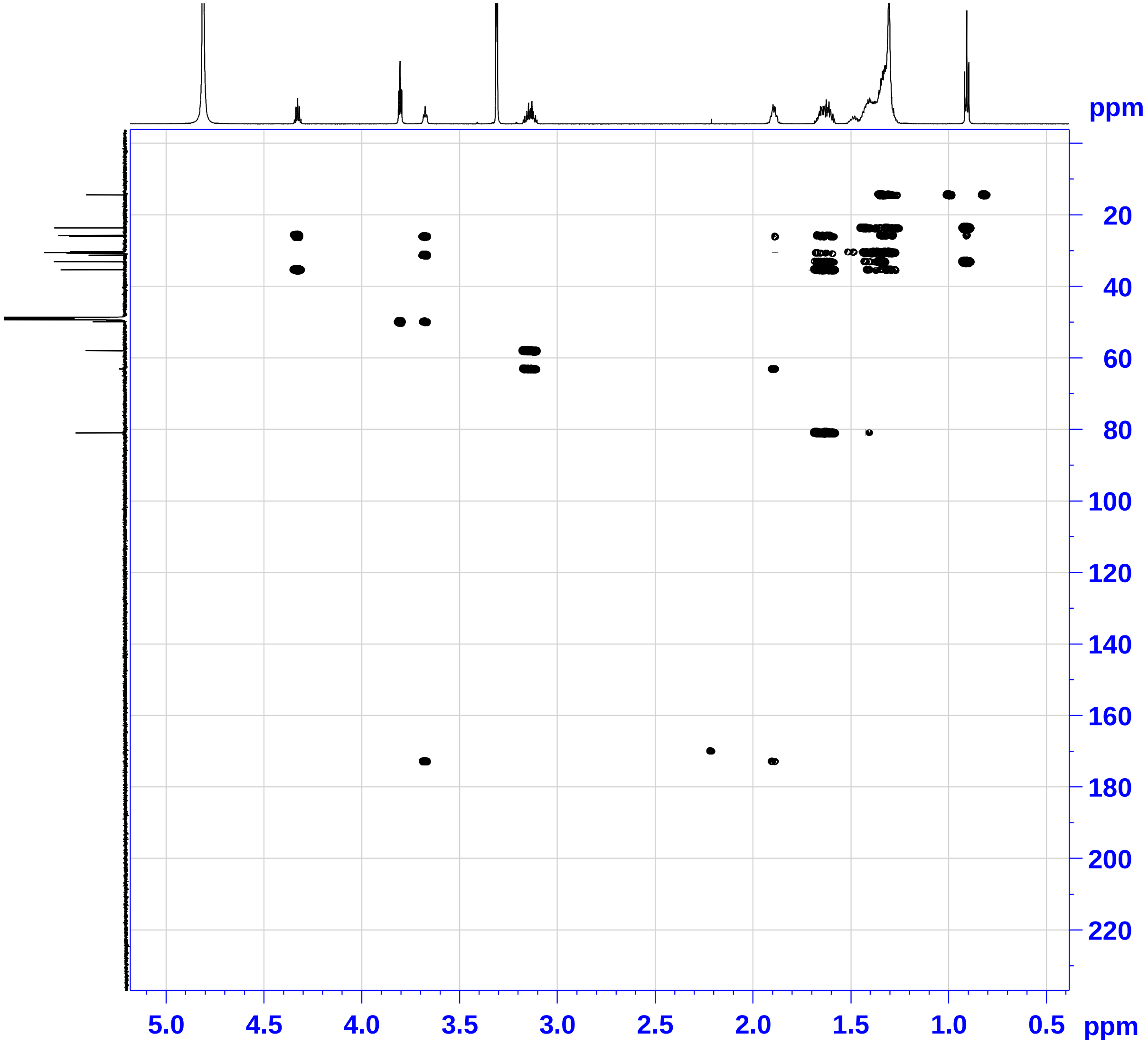

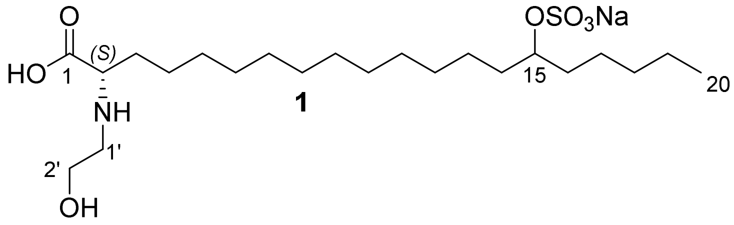

1

Figure S8. ^{1}H-^{15}N HMBC spectrum of anthonoic acid A (**1**) in CD_3OD.

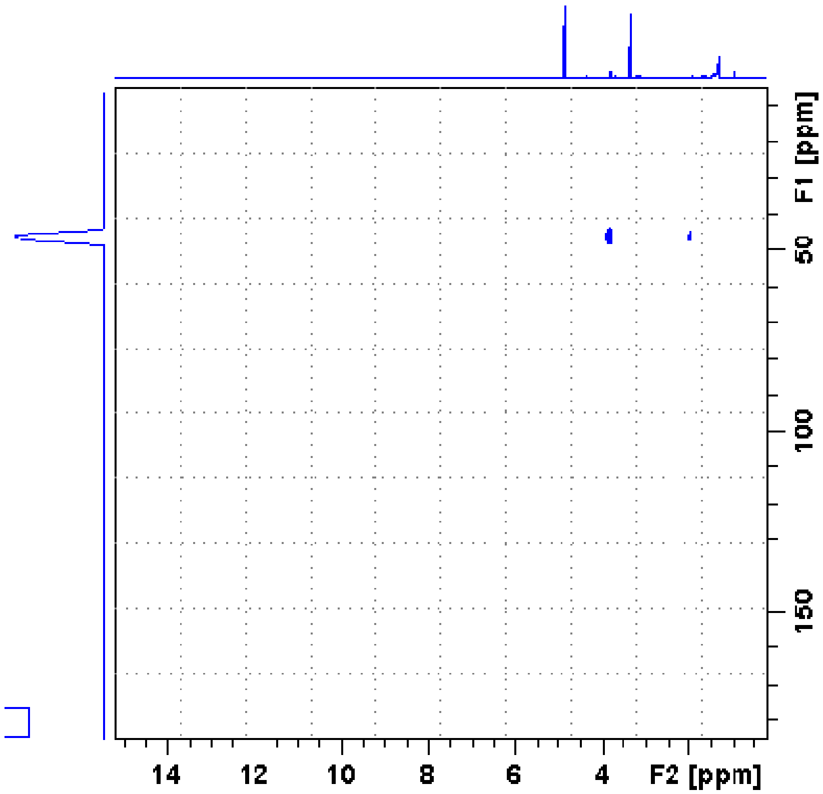

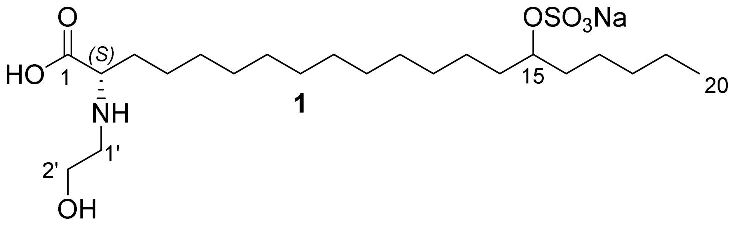

Figure S9. HRESIMS spectrum of anthonoic acid A (**1**).

Negative ion mode

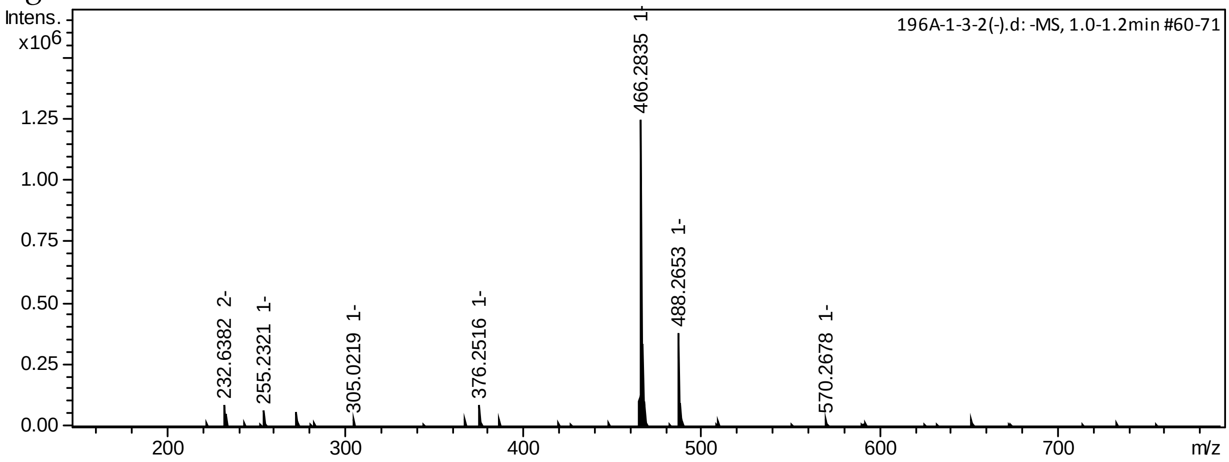

Positive ion mode

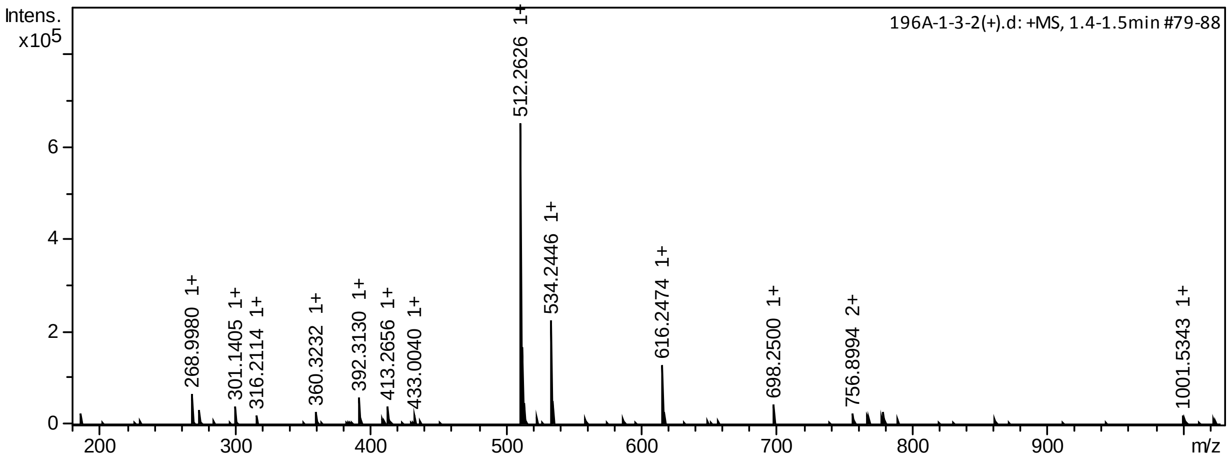

Figure S10. (–)ESIMS/MS spectrum of $[M_{Na} – Na]^{-}$ precursor ion at *m/z* 466 of anthonoic acid A (**1**).

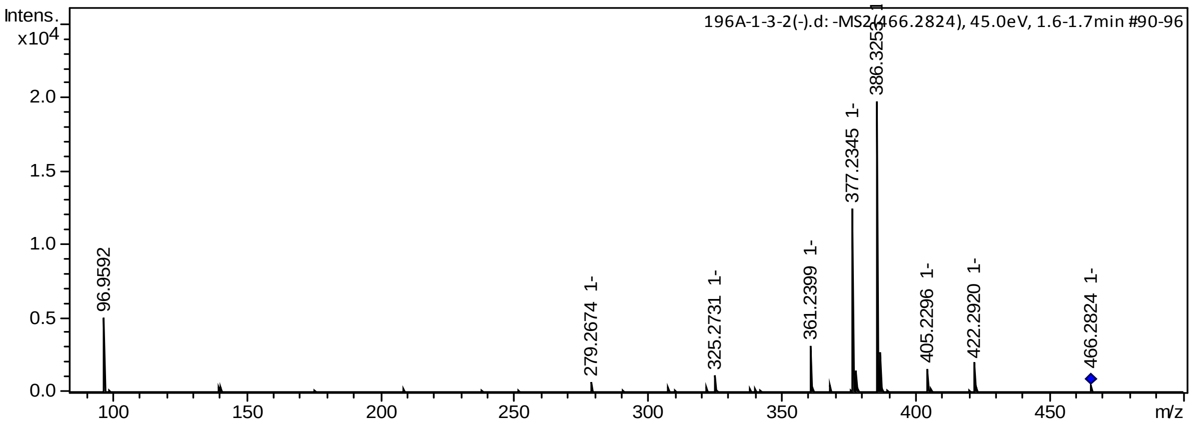

Figure S11. Fragmentation of **1** in (–)HRESIMS/MS.

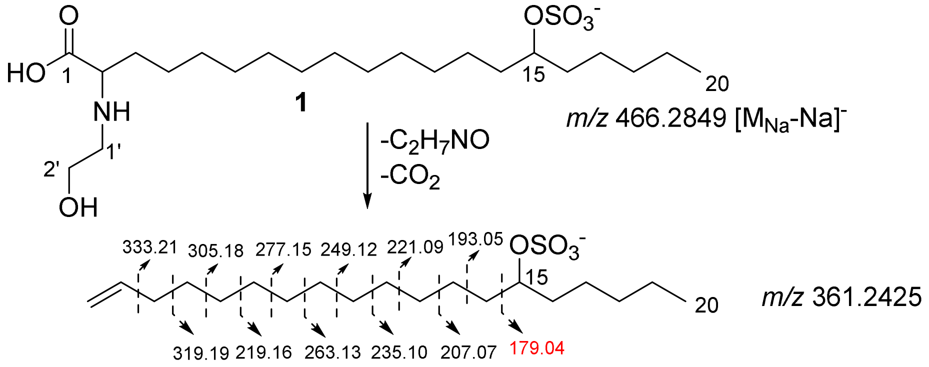

Table S1. MS2 spectra of anthonoic acid A (**1**) obtained under electrospray ionization in the negative ion detection mode.

Fragment ions	Calculated *m/z*	Measured *m/z* (rel. intensity, %)
$[C_{22}H_{44}NO_7S]^-$	466.2844	466.2849 (9.1)
$[C_{21}H_{44}NO_5S]^-$	422.2946	422.2951 (15.4)
$[C_{20}H_{37}O_6S]^-$	405.2316	405.2322 (16.0)
$[C_{22}H_{44}NO_4]^-$	386.3276	386.3284 (100)
$[C_{19}H_{37}O_5S]^-$	377.2367	377.2376 (66.8)
$[C_{19}H_{37}O_4S]^-$	361.2418	361.2425 (17.5)
$[C_{18}H_{35}O_4S]^-$	347.2262	347.2256 (0.1)
$[C_{17}H_{33}O_4S]^-$	333.2105	333.2107 (0.1)
$[C_{16}H_{31}O_4S]^-$	319.1949	319.1951 (0.4)
$[C_{15}H_{29}O_4S]^-$	305.1792	305.1793 (0.5)
$[C_{14}H_{27}O_4S]^-$	291.1636	291.1638 (1.0)
$[C_{13}H_{25}O_4S]^-$	277.1479	277.1479 (0.5)
$[C_{12}H_{23}O_4S]^-$	263.1323	263.1323 (0.4)
$[C_{11}H_{21}O_4S]^-$	249.1166	249.1167 (0.4)
$[C_{10}H_{19}O_4S]^-$	235.1010	235.1011 (0.5)
$[C_9H_{17}O_4S]^-$	221.0853	221.0856 (0.1)
$[C_8H_{15}O_4S]^-$	207.0697	207.0700 (0.1)
$[C_7H_{13}O_4S]^-$	193.0540	193.0544 (0.02)
$[C_6H_{11}O_4S]^-$	179.0384	179.0389 (0.2)

Figure S12. ^{1}H NMR spectrum of anthonoic acid B (**2**) in CD_3OD (700 MHz).

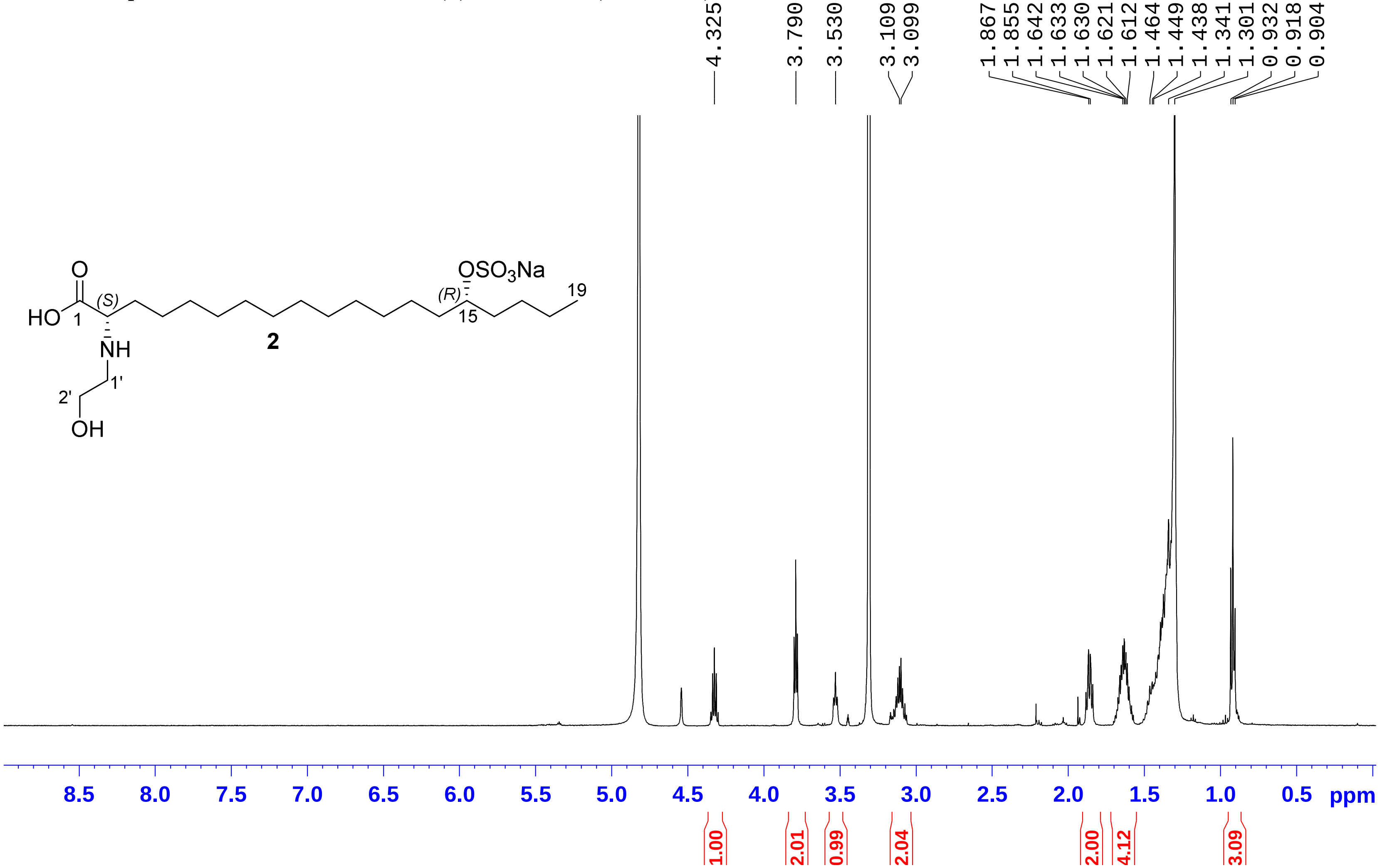

Figure S13. Partial of the ^{1}H NMR spectrum of anthonoic acid B (**2**) in CD_3OD (700 MHz).

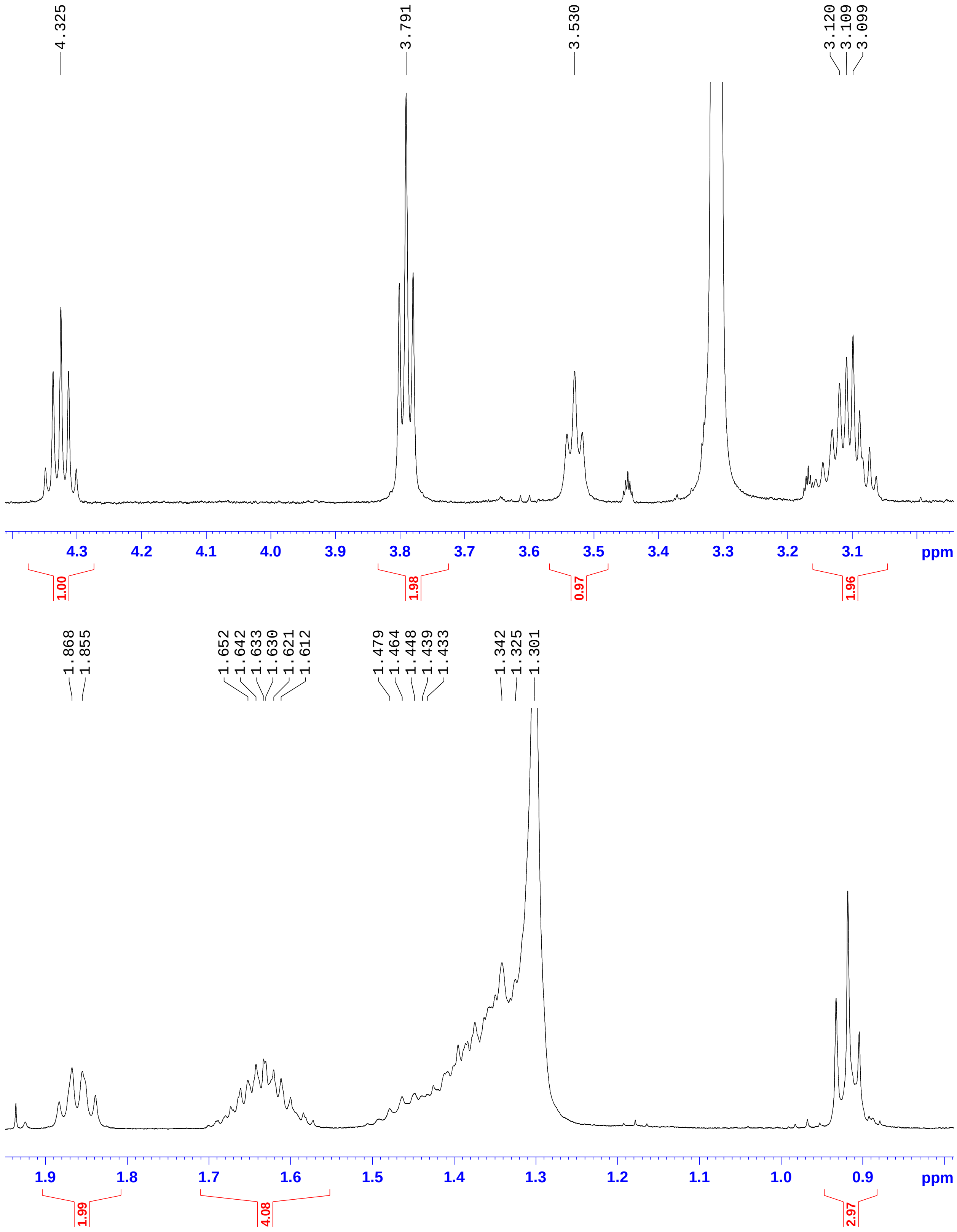

Figure S14. ^{13}C NMR spectrum of anthonoic acid B (**2**) in CD_3OD (175 MHz).

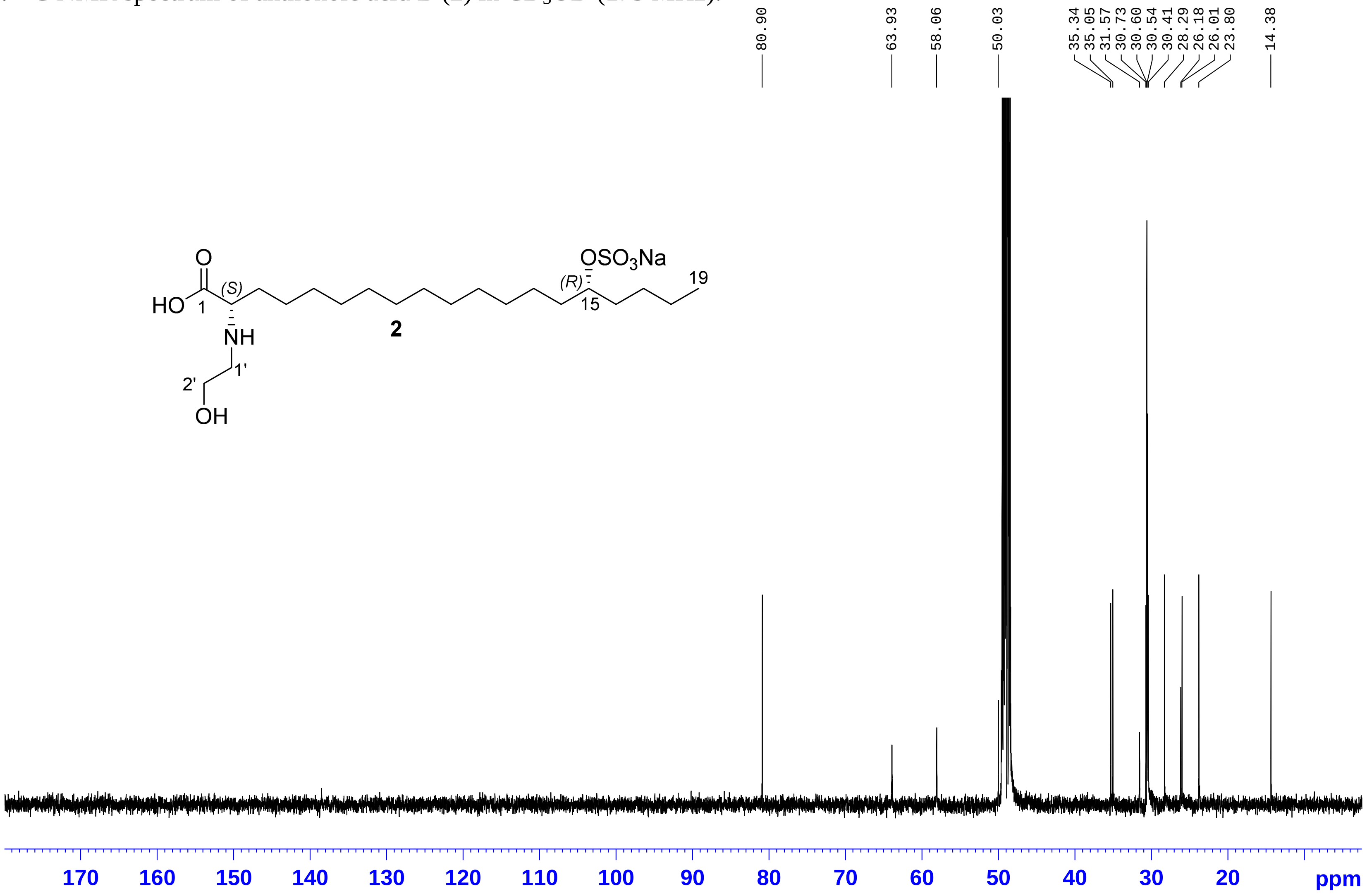

Figure S15. Partial of the ^{13}C NMR spectrum of anthonoic acid B (**2**) in CD_3OD (175 MHz).

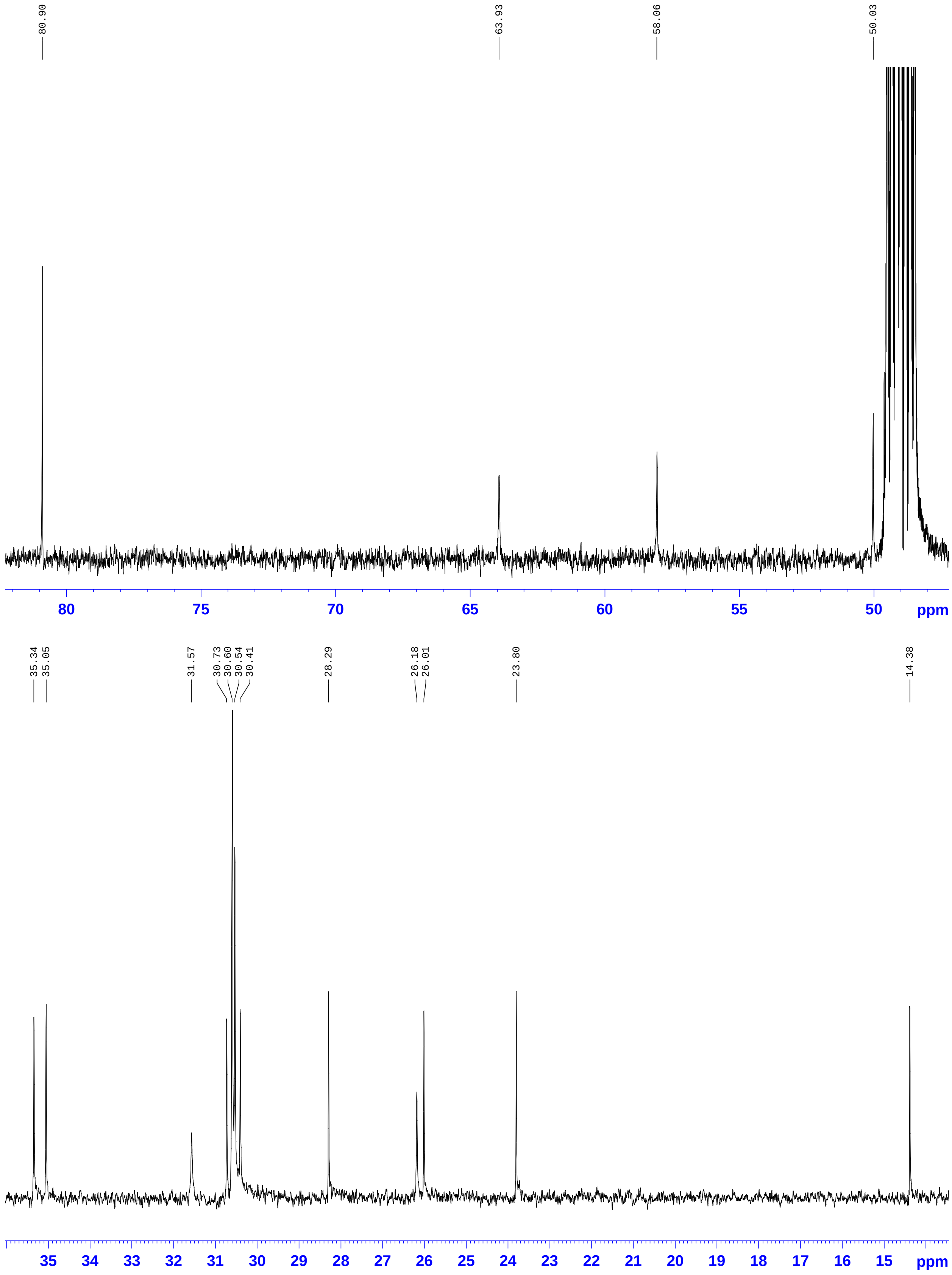

Figure S16. ^1H-^1H COSY spectrum of anthonoic acid B (**2**) in CD_3OD.

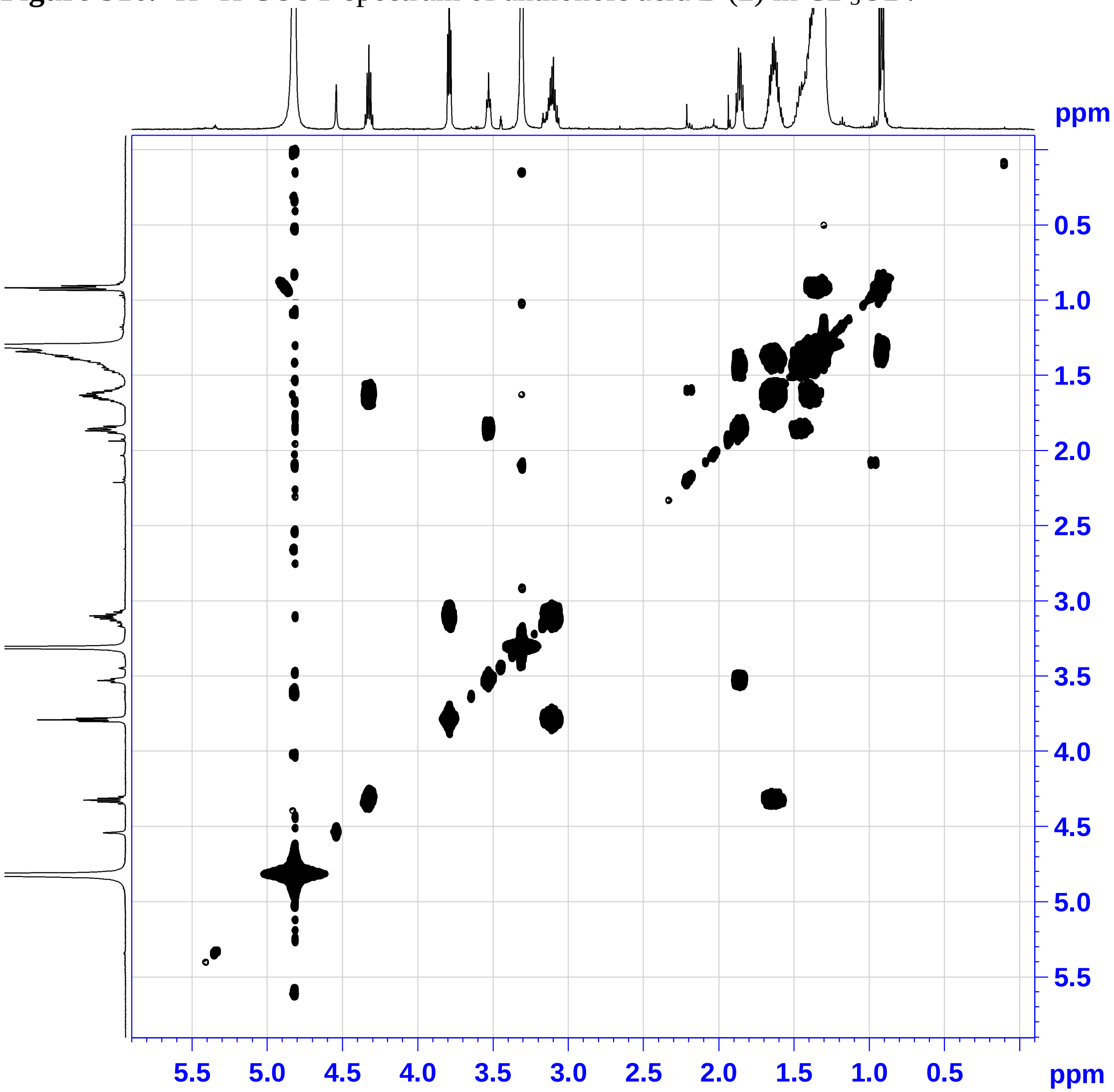

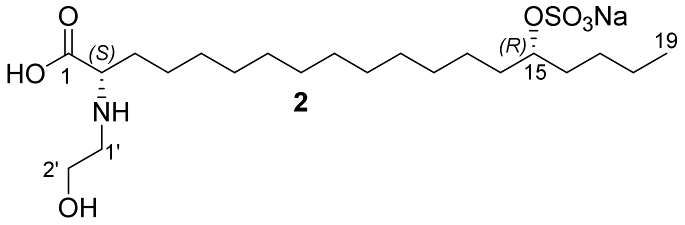

Figure S17. HSQC spectrum of anthonoic acid B (**2**) in CD_3OD.

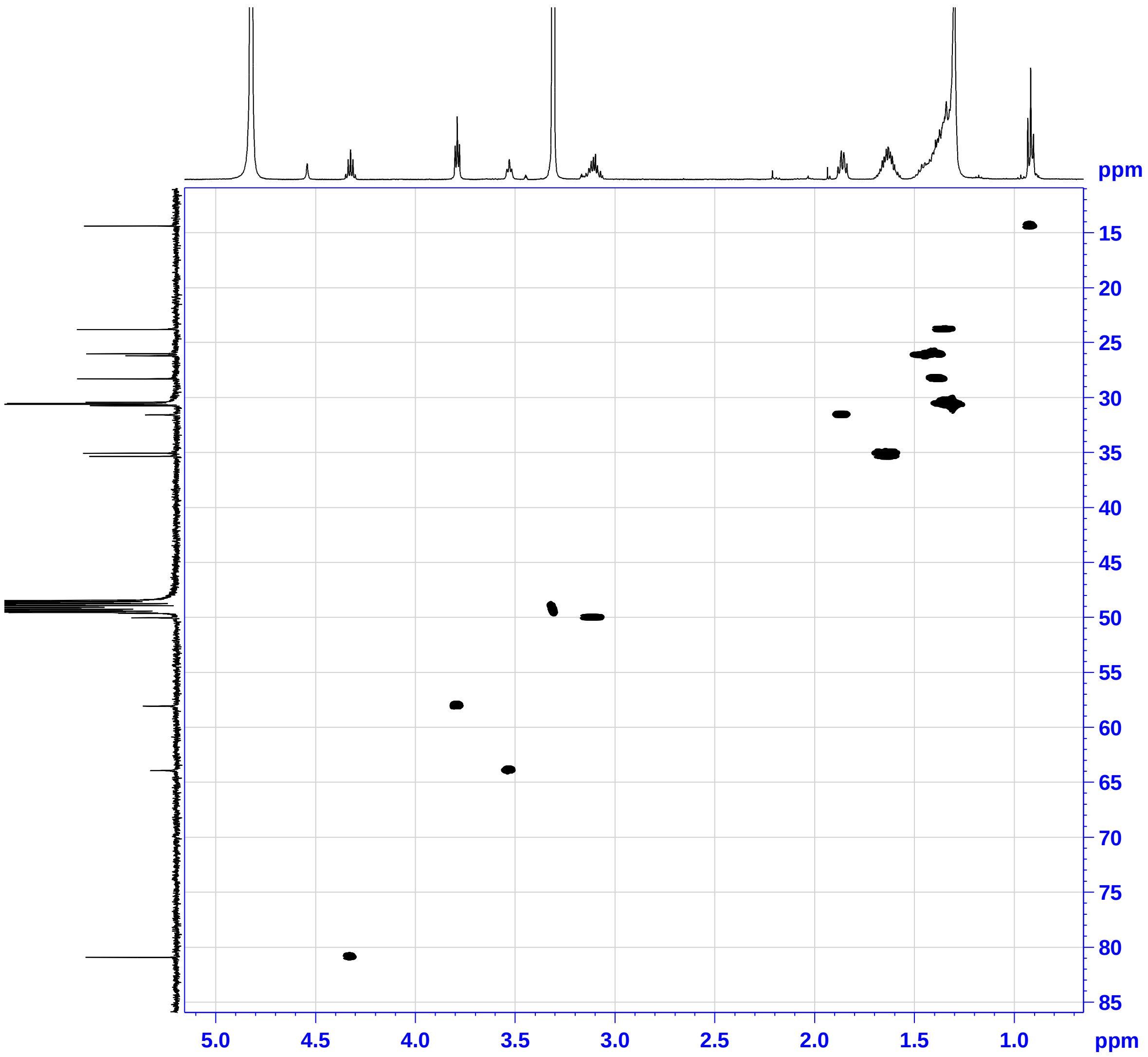

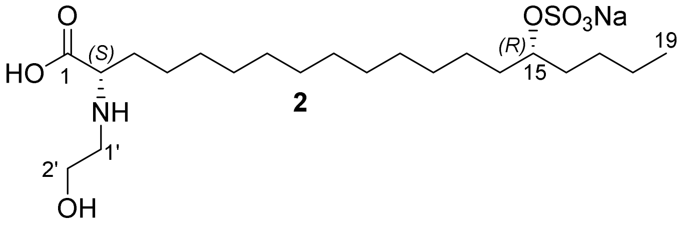

2

Figure S18. HMBC spectrum of anthonoic acid B (**2**) in CD_3OD.

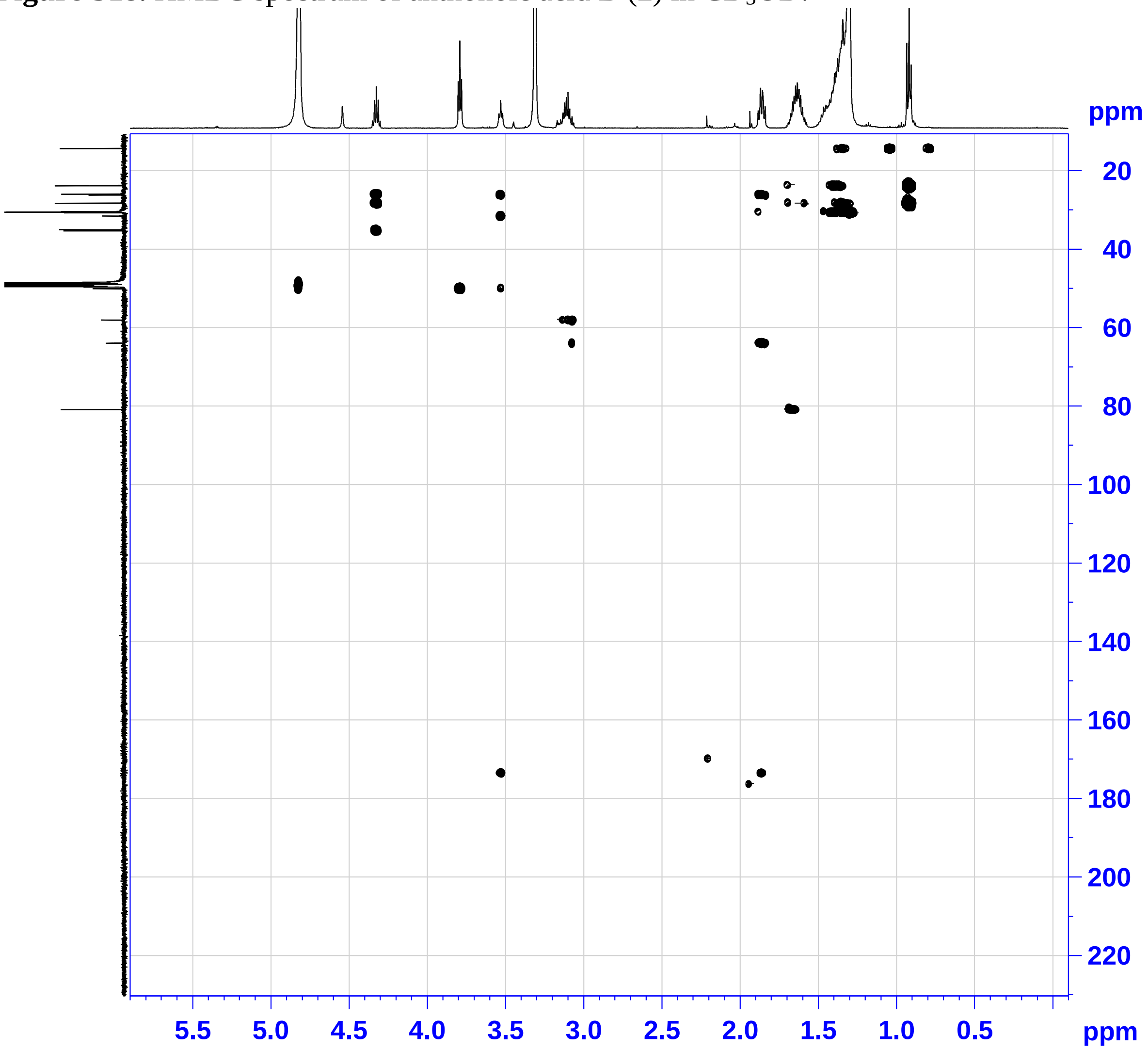

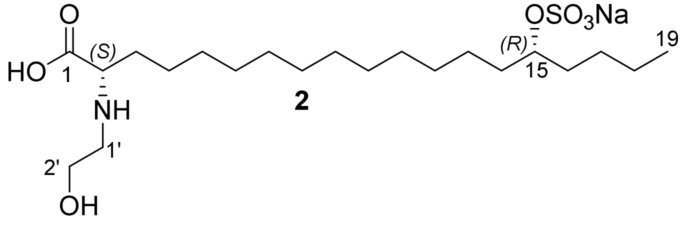

2

Figure S19. HRESIMS spectrum of anthonoic acid B (**2**).

Negative ion mode

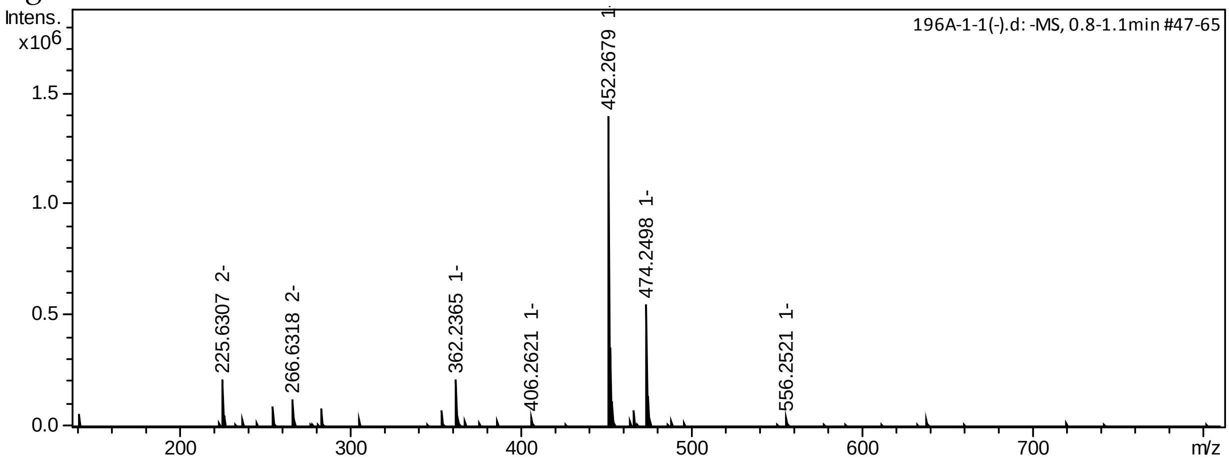

Positive ion mode

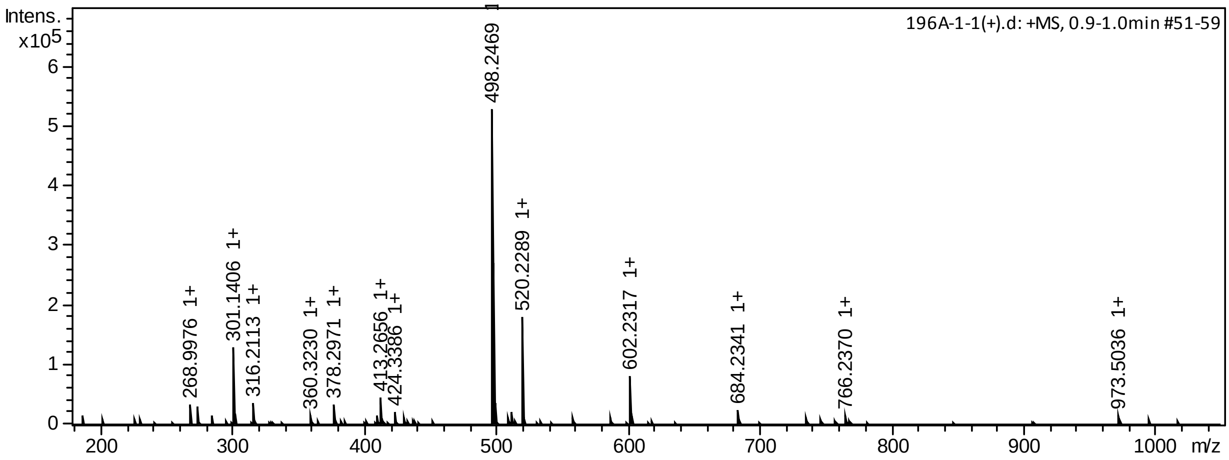

Figure S20. (–)ESIMS/MS spectrum of $[M_{Na} – Na]^–$ precursor ion at *m/z* 452 of anthonoic acid B (**2**).

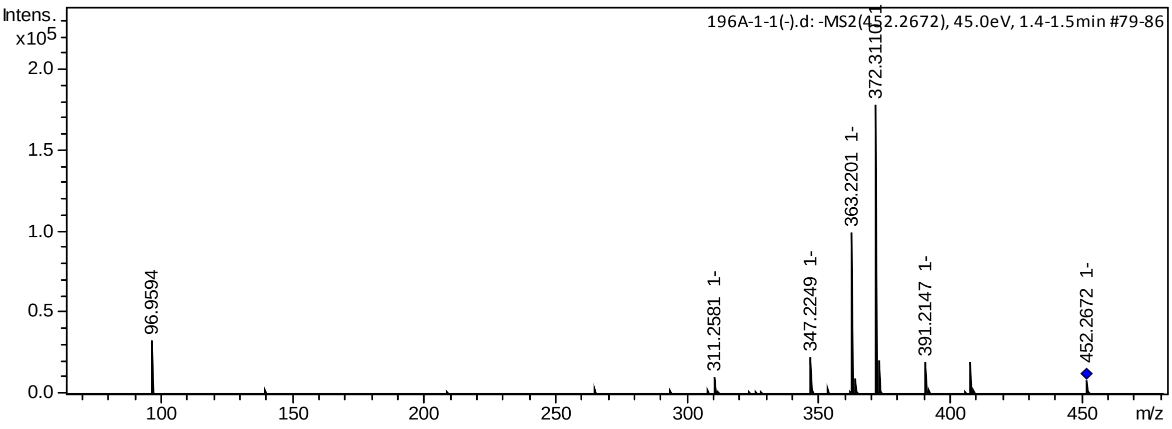

Figure S21. Fragmentation of **2** in (–)HRESIMS/MS.

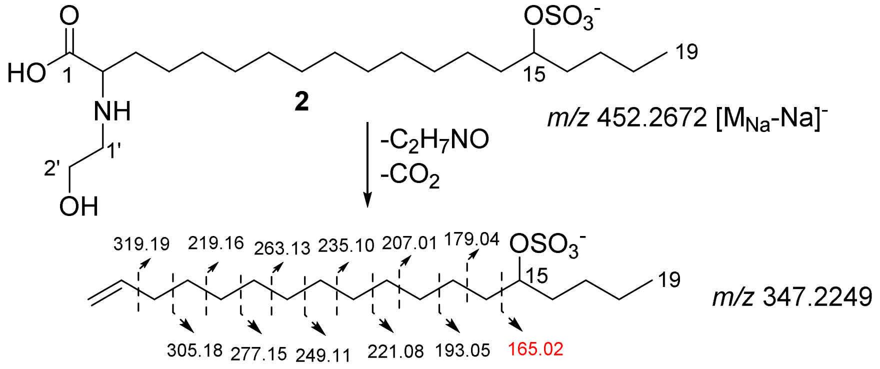

Table S2. MS[2] spectra of anthonoic acid B (**2**) obtained under electrospray ionization in the negative ion detection mode.

Fragment ions	Calculated *m/z*	Measured *m/z* (rel. intensity, %)
$[C_{21}H_{42}NO_7S]^-$	452.2687	452.2672 (4.9)
$[C_{20}H_{42}NO_5S]^-$	408.2789	408.2776 (11.2)
$[C_{19}H_{35}O_6S]^-$	391.2160	391.2147 (11.1)
$[C_{21}H_{42}NO_4]^-$	372.3119	372.3110 (100)
$[C_{18}H_{35}O_5S]^-$	363.2211	363.2201 (55.7)
$[C_{18}H_{35}O_4S]^-$	347.2262	347.2249 (13.0)
$[C_{17}H_{33}O_4S]^-$	333.2105	333.2092 (0.06)
$[C_{16}H_{31}O_4S]^-$	319.1949	319.1931 (0.1)
$[C_{15}H_{29}O_4S]^-$	305.1792	305.1778 (0.2)
$[C_{14}H_{27}O_4S]^-$	291.1636	291.1616 (0.3)
$[C_{13}H_{25}O_4S]^-$	277.1479	277.1466 (0.5)
$[C_{12}H_{23}O_4S]^-$	263.1323	263.1307 (0.2)
$[C_{11}H_{21}O_4S]^-$	249.1166	249.1148 (0.1)
$[C_{10}H_{19}O_4S]^-$	235.1010	235.1003 (0.1)
$[C_9H_{17}O_4S]^-$	221.0853	221.0844 (0.2)
$[C_8H_{15}O_4S]^-$	207.0697	207.0095 (0.04)
$[C_7H_{13}O_4S]^-$	193.0540	193.0520 (0.02)
$[C_6H_{11}O_4S]^-$	179.0384	179.0388 (0.02)
$[C_5H_9O_4S]^-$	165.0227	165.0233 (0.03)

Figure S22. ^{1}H NMR spectrum of anthonoic acid C (**3**) in CD_3OD (700 MHz).

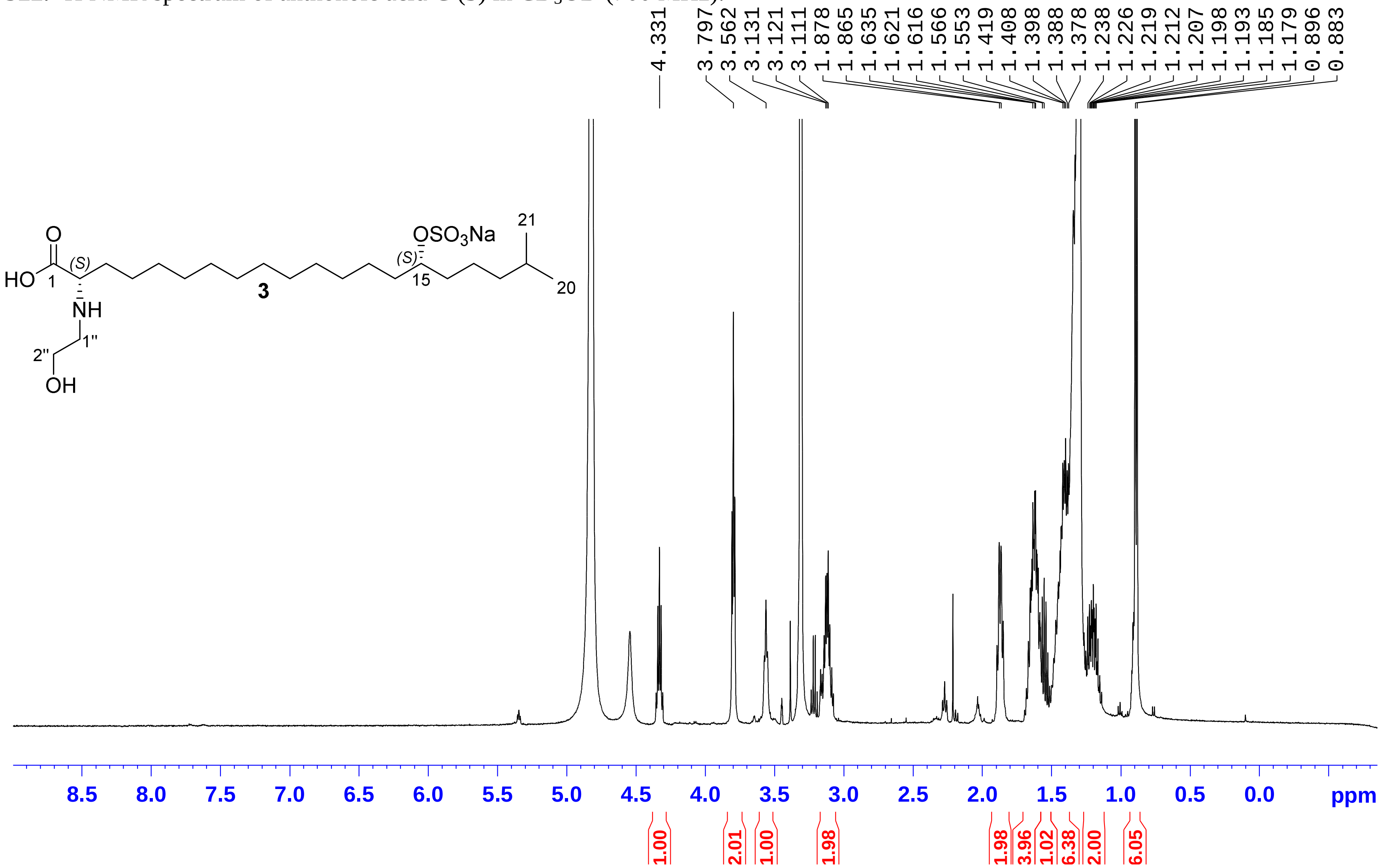

Figure S23. Partial of the ^{1}H NMR spectrum of anthonoic acid C (**3**) in CD_3OD (700 MHz).

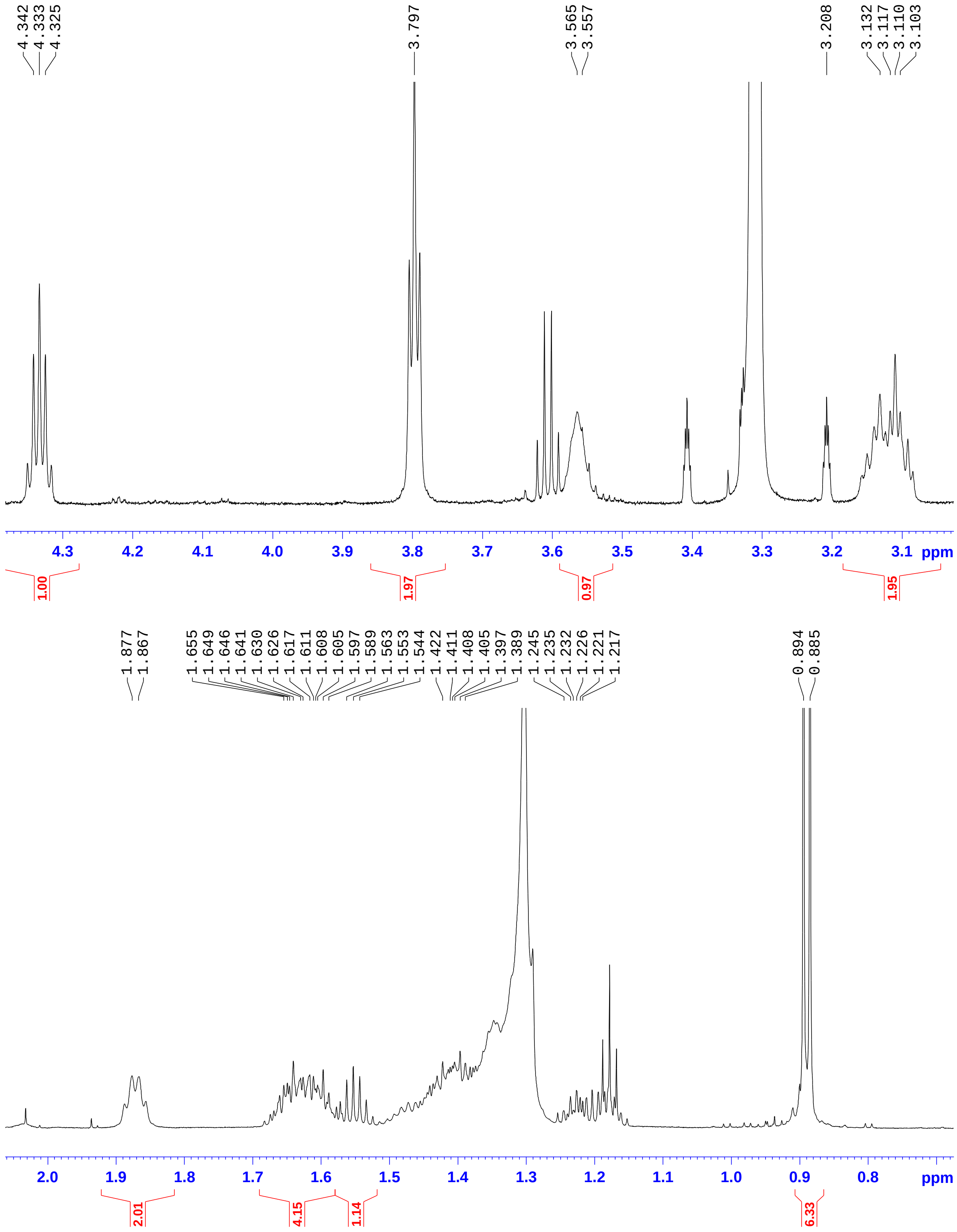

Figure S24. ^{13}C NMR spectrum of anthonoic acid C (**3**) in CD_3OD (175 MHz).

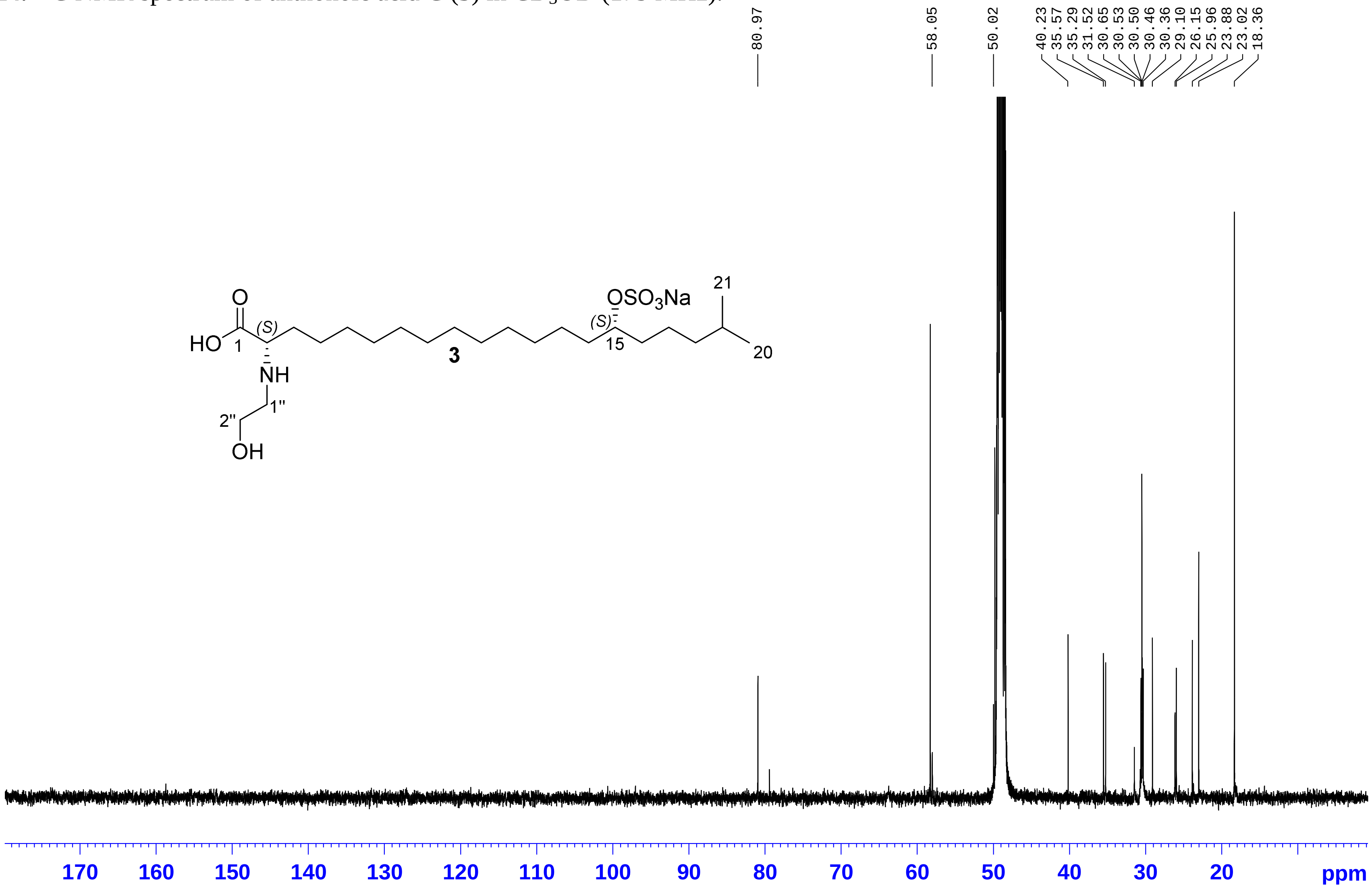

Figure 25. Partial of the ^{13}C NMR spectrum of anthonoic acid C (**3**) in CD_3OD (175 MHz).

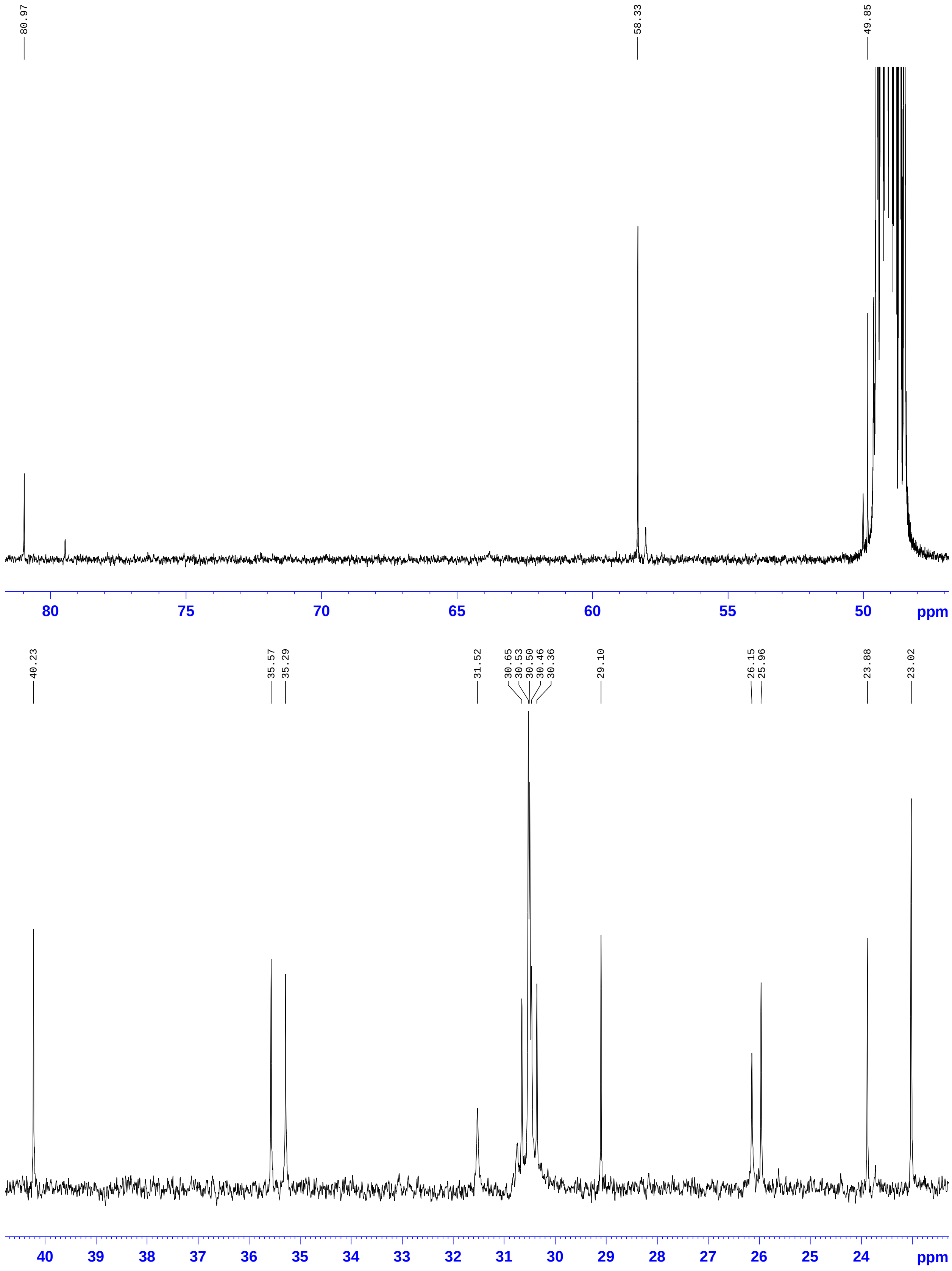

Figure S26. ^{1}H-^{1}H COSY spectrum of anthonoic acid C (**3**) in CD_3OD.

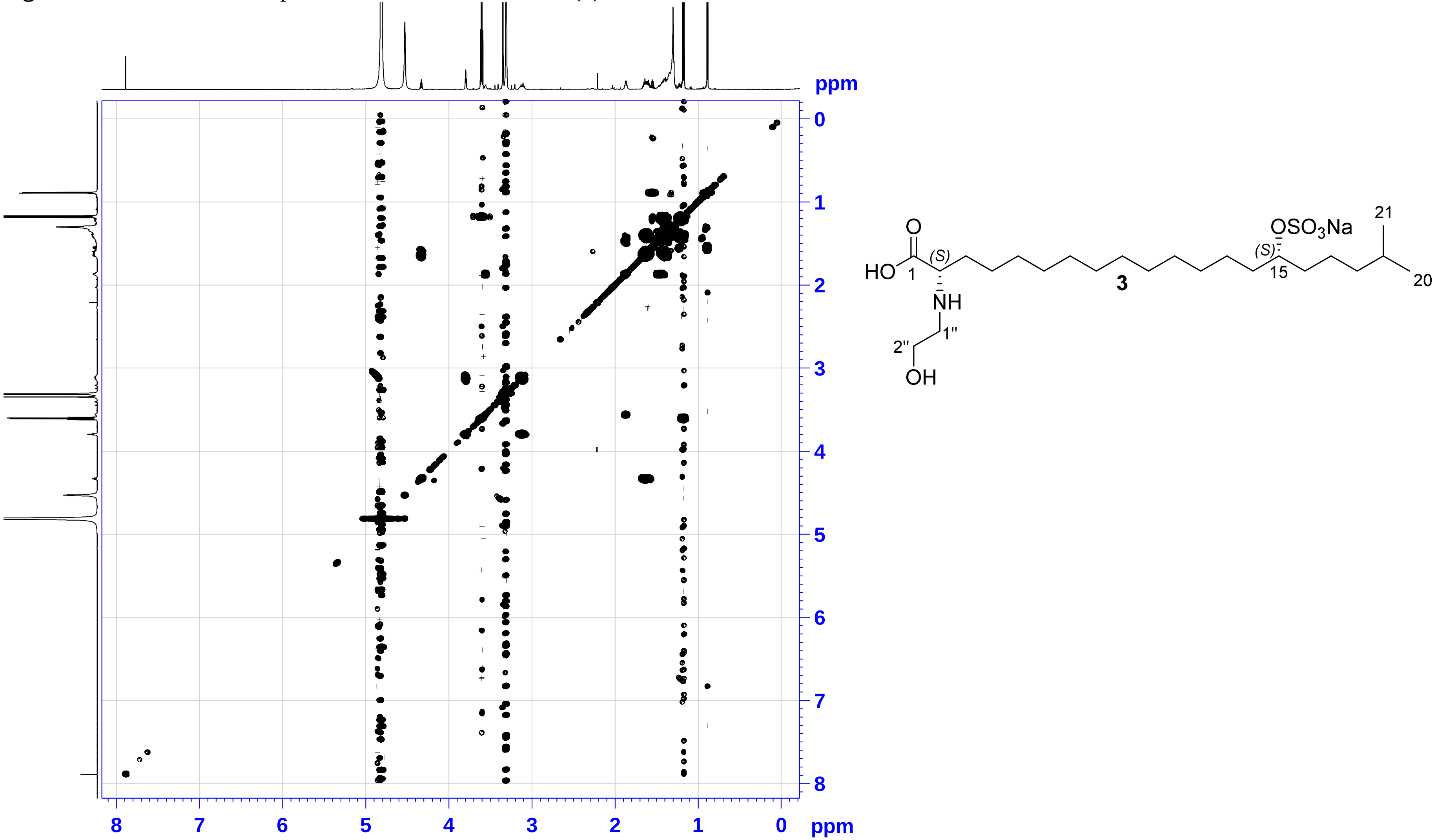

Figure S27. HSQC spectrum of anthonoic acid C (**3**) in CD_3OD.

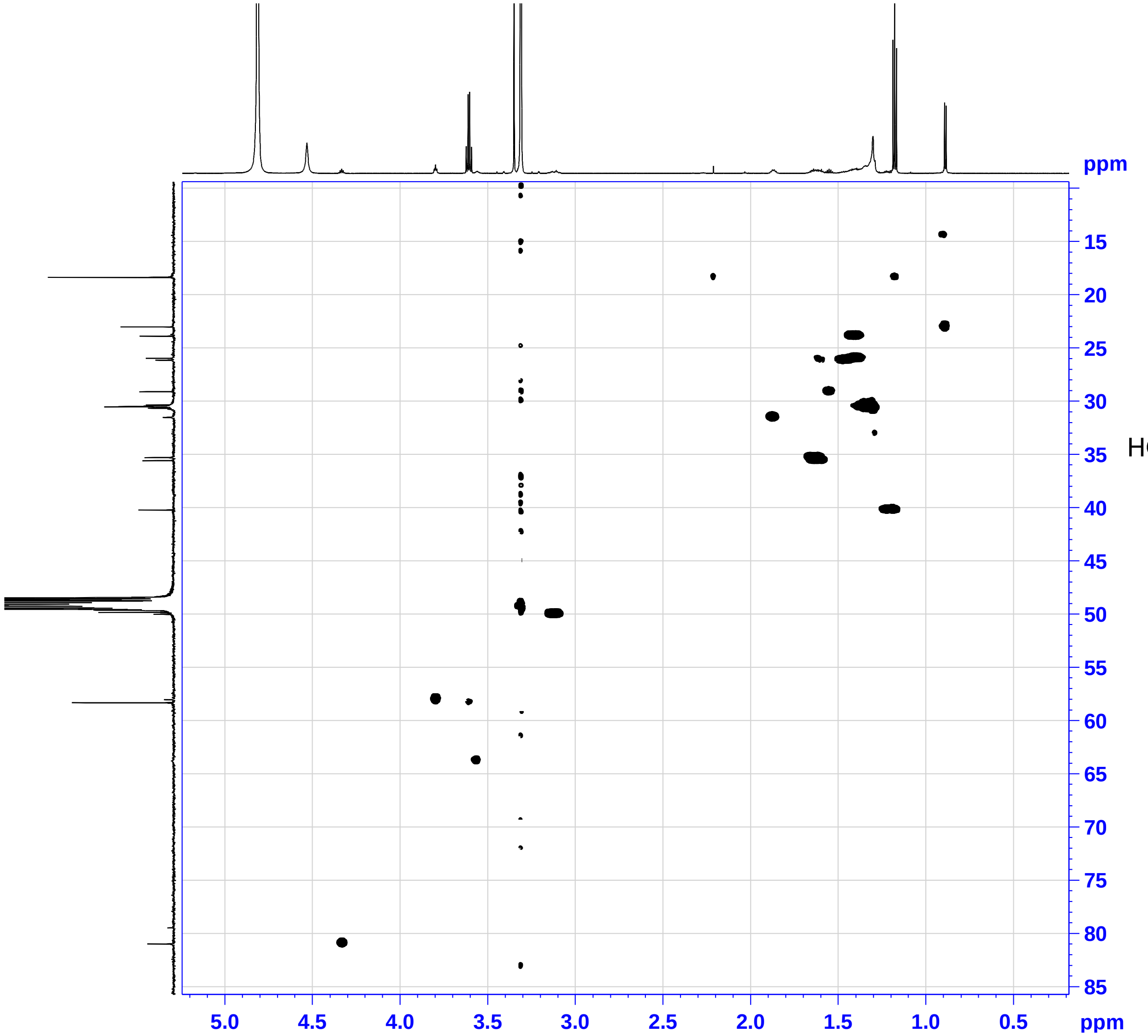

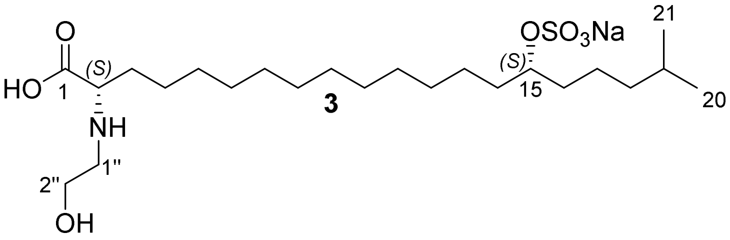

Figure S28. HMBC spectrum of anthonoic acid C (**3**) in CD_3OD.

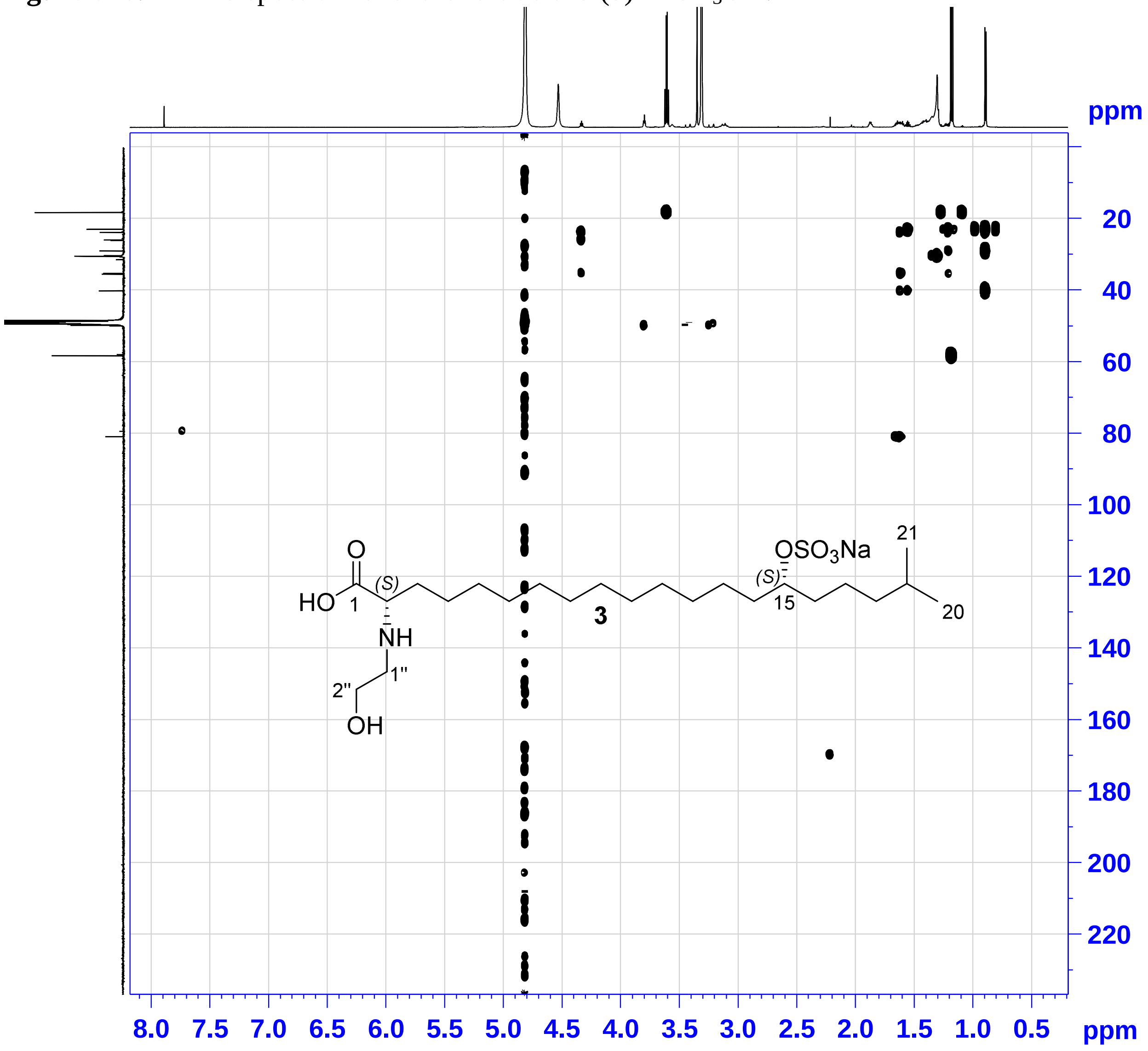

Figure S29. HRESIMS spectrum of anthonoic acid C (**3**).

Negative ion mode

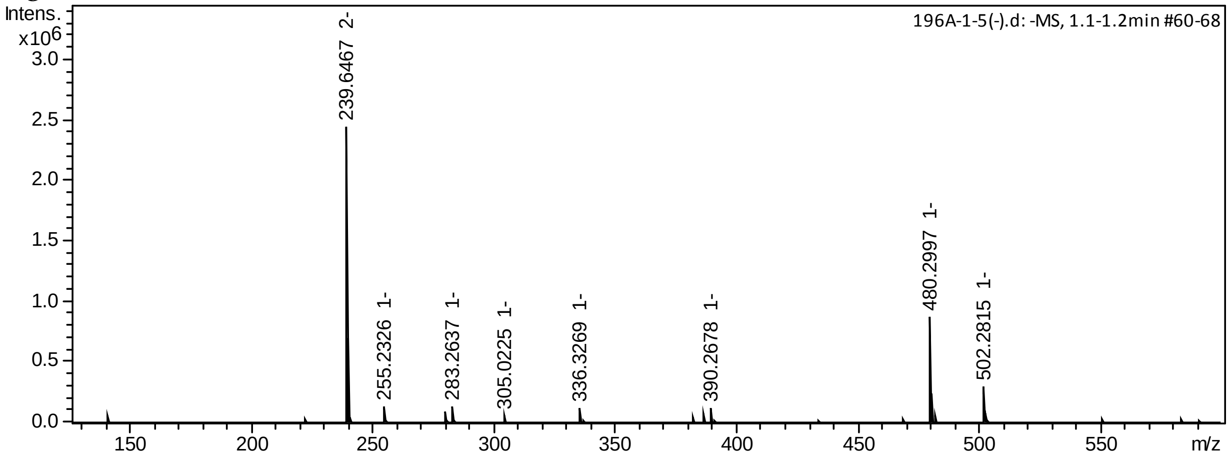

Positive ion mode

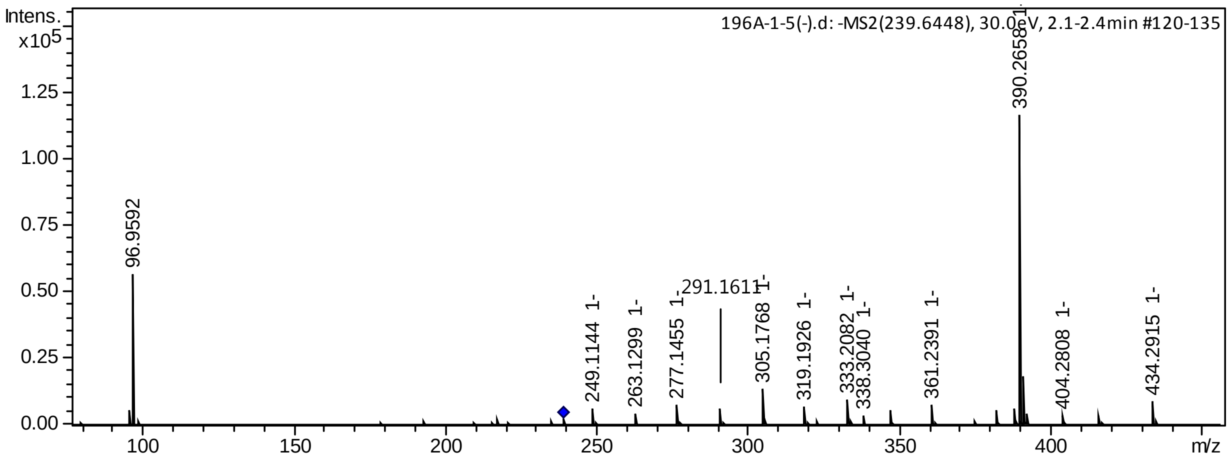

Figure S30. (–)ESIMS/MS spectrum of [M_{Na} – Na]$^{-}$ precursor ion at *m/z* 480 of anthonoic acid C (**3**).

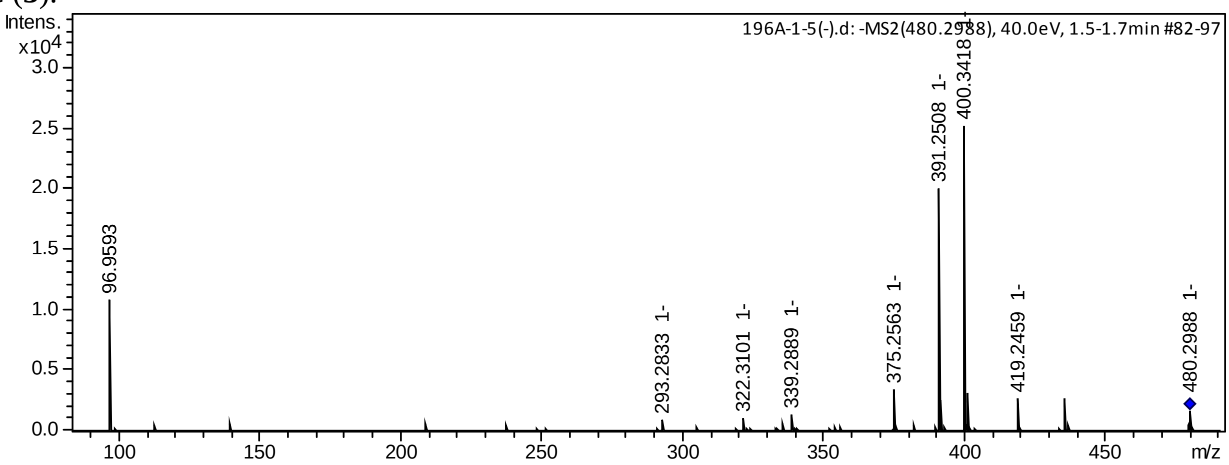

Figure S31. Fragmentation of **3** in (–)HRESIMS/MS

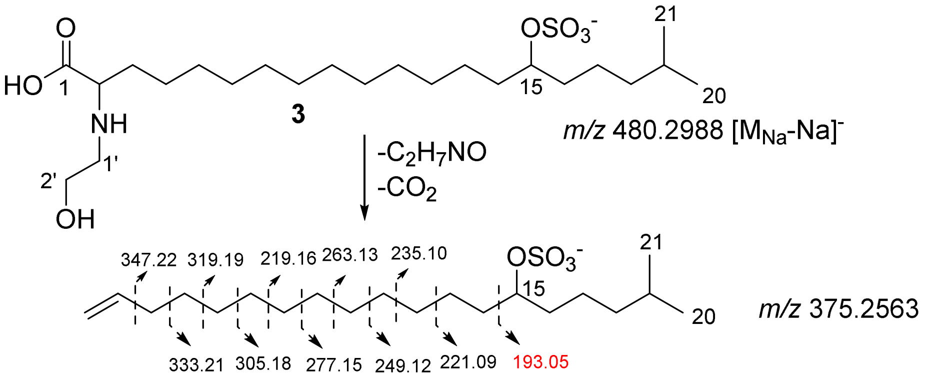

Table S3. MS^2 spectra of anthonoic acid C (**3**) obtained under electrospray ionization in the negative ion detection mode.

Fragment ions	Calculated *m/z*	Measured *m/z* (rel. intensity, %)
$[C_{23}H_{46}NO_7S]^-$	480.3000	480.2988 (6.8)
$[C_{22}H_{46}NO_5S]^-$	436.2992	436.3085 (10.8)
$[C_{21}H_{39}O_6S]^-$	419.2473	419.2459 (10.8)
$[C_{23}H_{46}NO_4]^-$	400.3432	400.3418 (100)
$[C_{20}H_{39}O_5S]^-$	391.2524	391.2508 (79.2)
$[C_{20}H_{39}O_4S]^-$	375.2575	375.2563 (14.1)
$[C_{19}H_{37}O_4S]^-$	361.2418	361.2433 (0.1)
$[C_{18}H_{35}O_4S]^-$	347.2262	347.2238 (0.2)
$[C_{17}H_{33}O_4S]^-$	333.2105	333.2095 (0.6)
$[C_{16}H_{31}O_4S]^-$	319.1949	319.1930 (0.9)
$[C_{15}H_{29}O_4S]^-$	305.1792	305.1779 (1.4)
$[C_{14}H_{27}O_4S]^-$	291.1636	291.1625 (0.7)
$[C_{13}H_{25}O_4S]^-$	277.1479	277.1460 (0.6)
$[C_{12}H_{23}O_4S]^-$	263.1323	263.1311(0.5)
$[C_{11}H_{21}O_4S]^-$	249.1166	249.1152 (0.7)
$[C_{10}H_{19}O_4S]^-$	235.1010	235.0990 (0.1)
$[C_9H_{17}O_4S]^-$	221.0853	221.0838 (0.06)
$[C_7H_{13}O_4S]^-$	193.0540	193.0520 (0.1)

Figure S32. ^{1}H NMR spectrum of mixture of anthamino acid A (**4**) and **1** in CD_3OD (700 MHz).

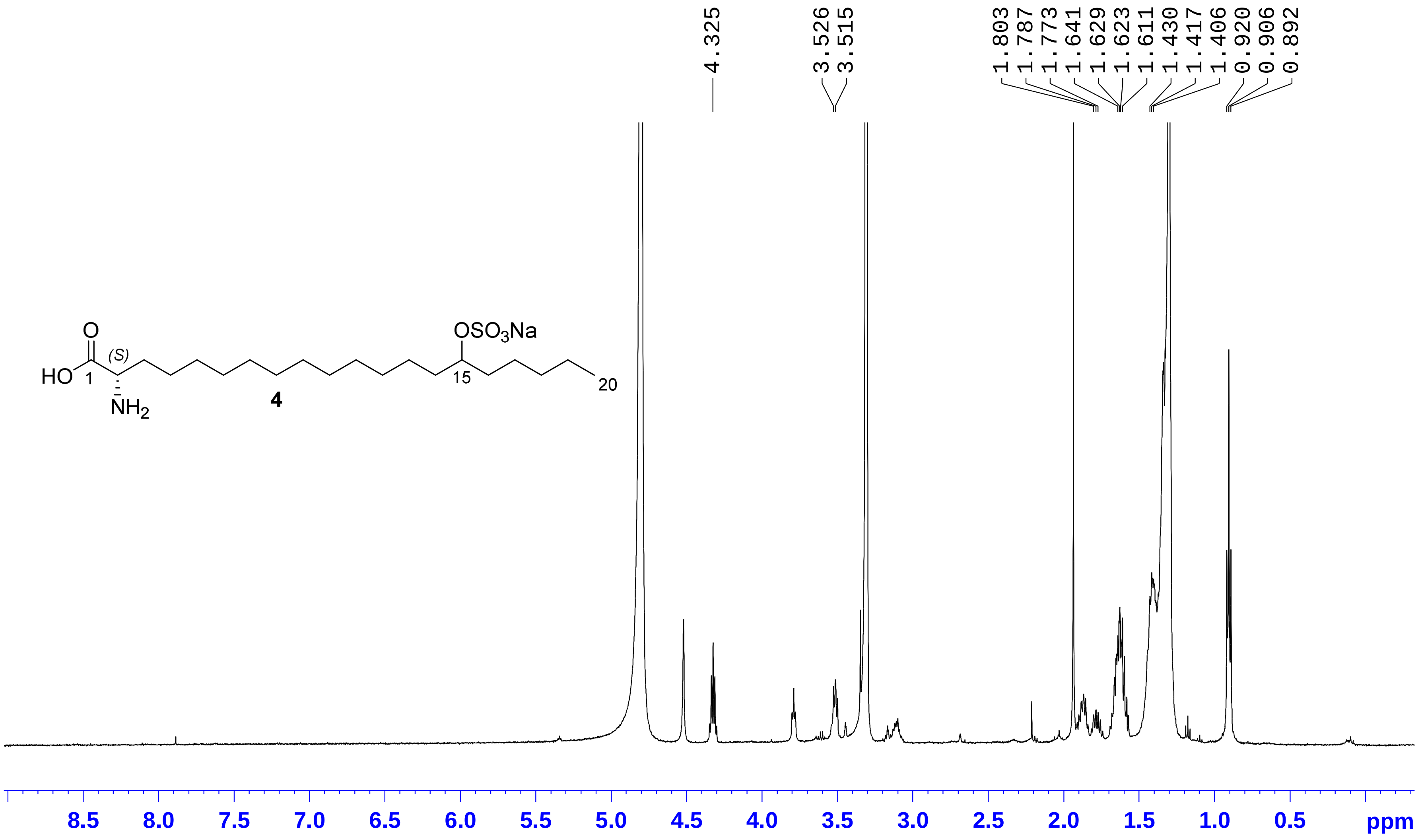

Figure S33. ^{13}C NMR spectrum of mixture of anthamino acid A (**4**) and **1** in CD_3OD (175 MHz).

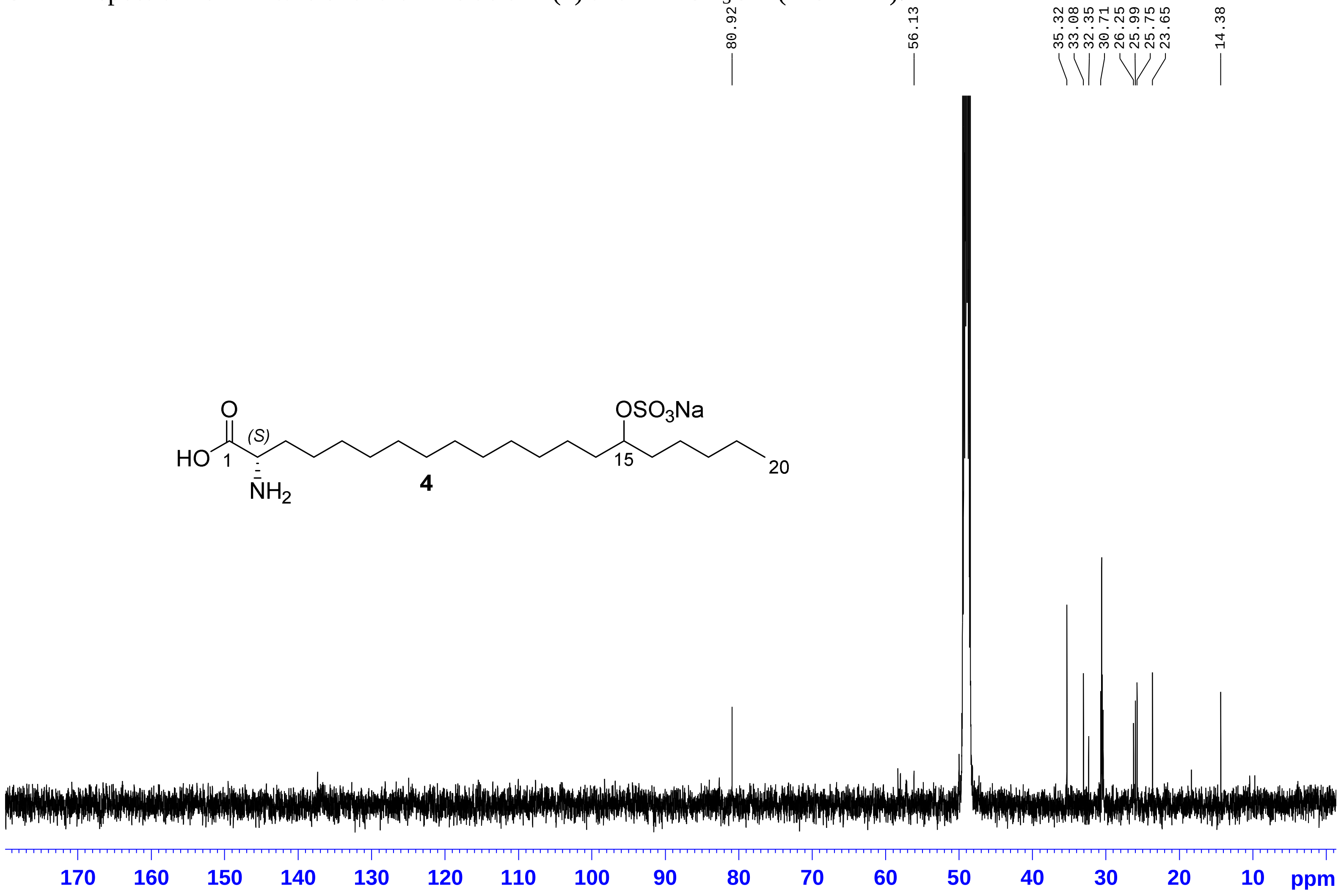

Figure S34. HRESIMS spectrum of anthamino acid A (**4**).

Negative ion mode

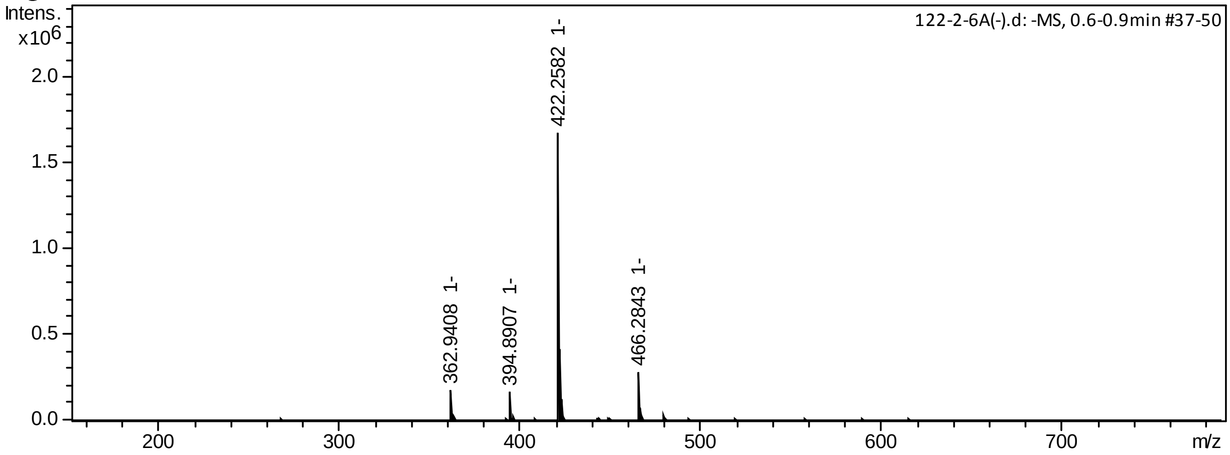

Figure S35. ¹H NMR spectrum of (*S*)-MTPA amide (**4b**) in CD_3OD (700 MHz).

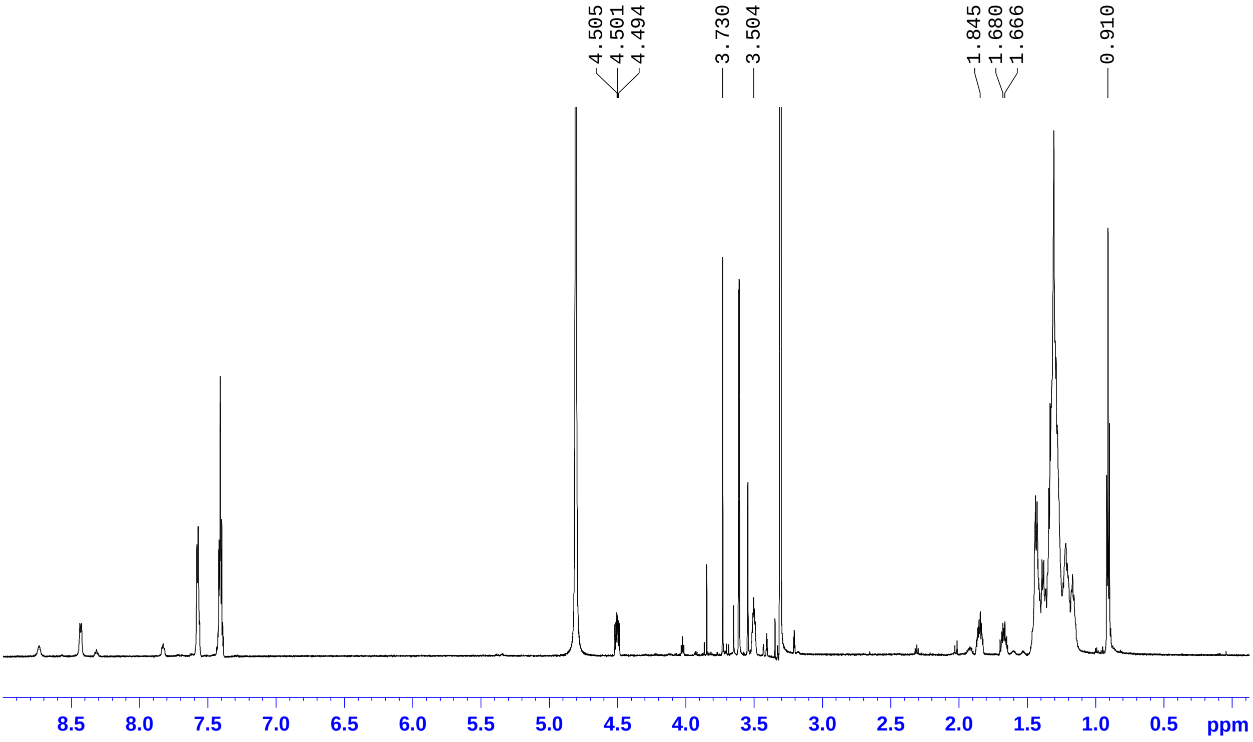

Figure S36. ¹H NMR spectrum of (*R*)-MTPA amide (**4c**) in CD_3OD (700 MHz).

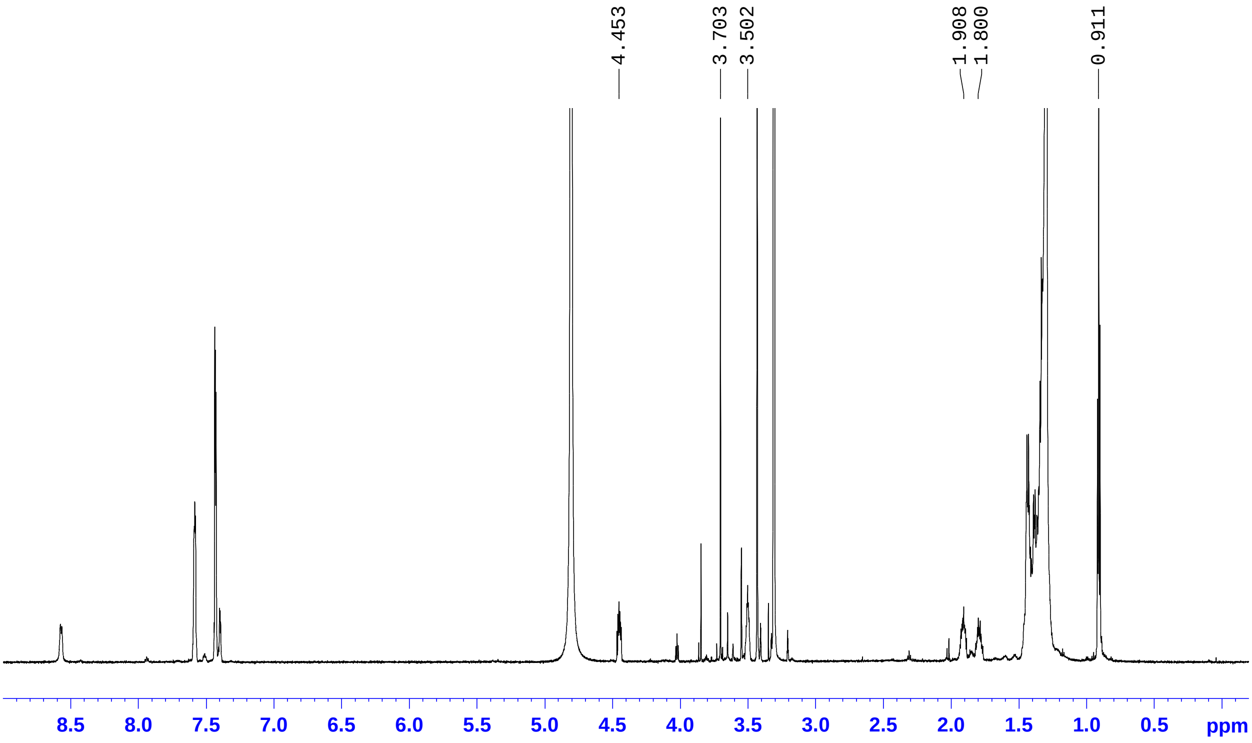

Figure S37. ^{1}H NMR spectrum of (*S*)-MTPA ester **6a** in CD_3OD (700 MHz).

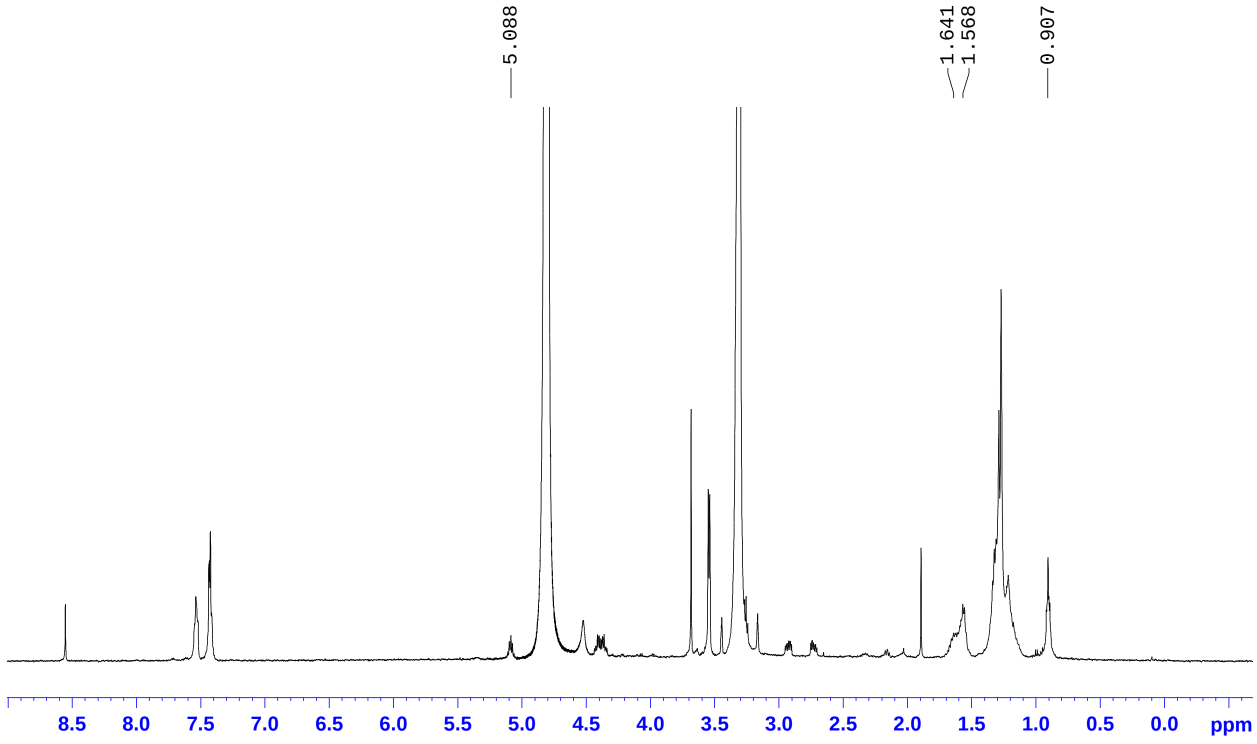

Figure S38. ^{1}H NMR spectrum of (*R*)-MTPA ester **6b** in CD_3OD (700 MHz).

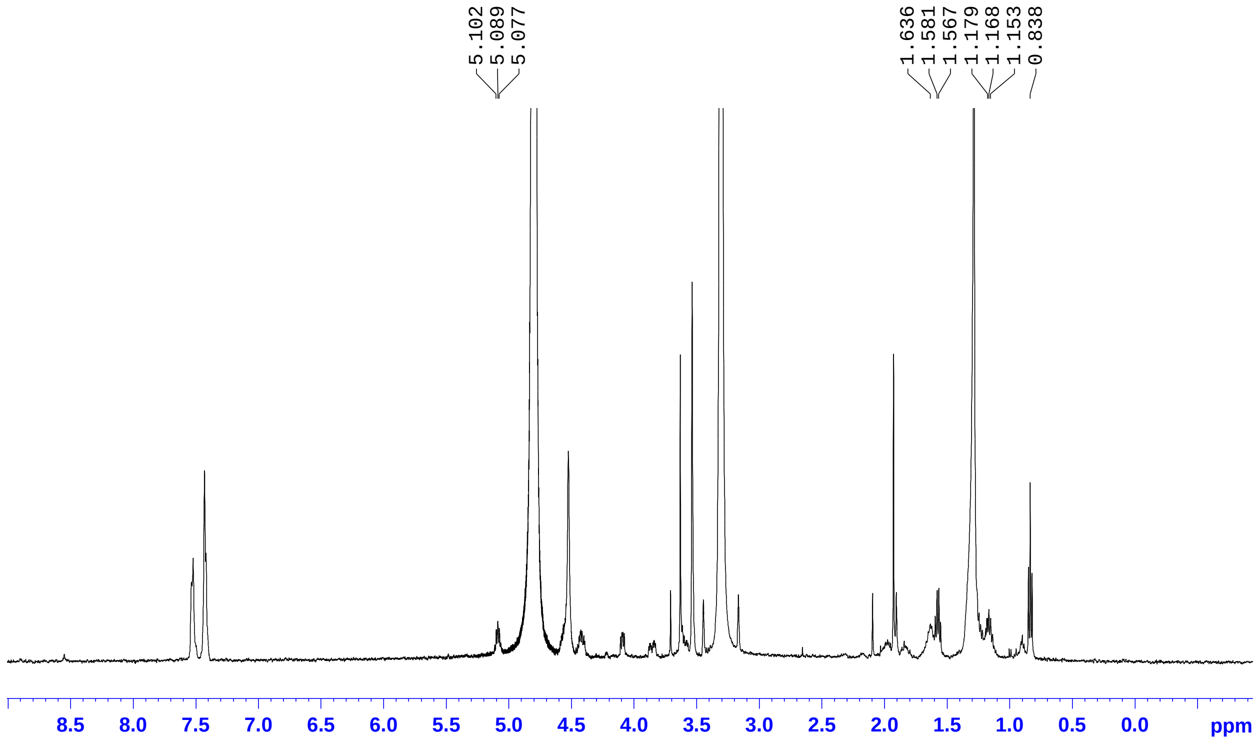

Figure S39. 1D selective TOCSY spectrum of (*S*)-MTPA ester **6a** with selective excitation of H_3-19 (700 MHz, CD_3OD).

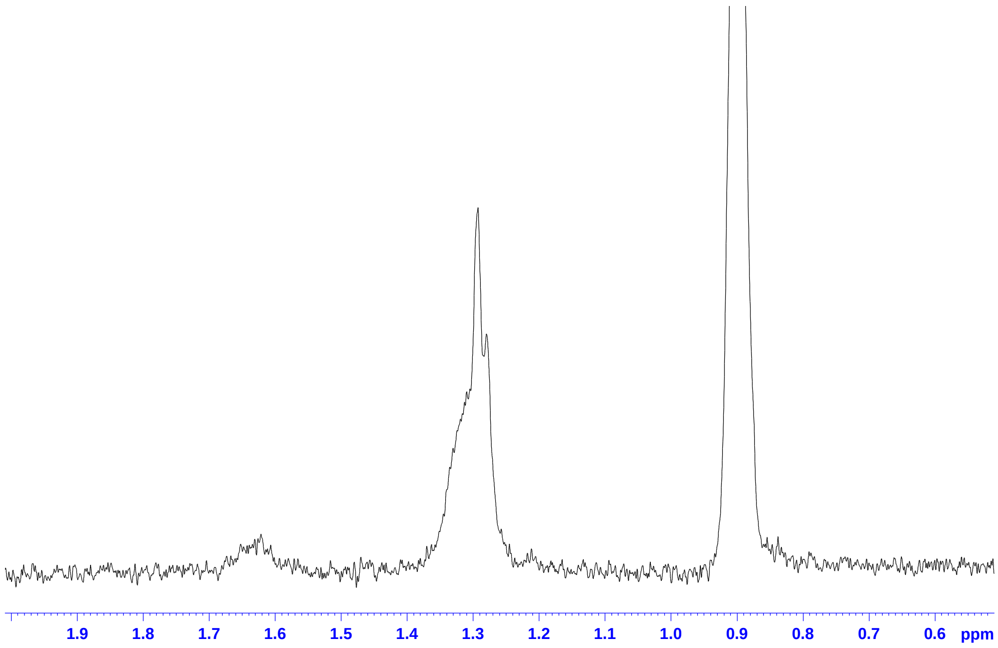

Figure S40. 1D selective TOCSY spectrum of (*R*)-MTPA ester **6b** with selective excitation of H_3-19 (700 MHz, CD_3OD).

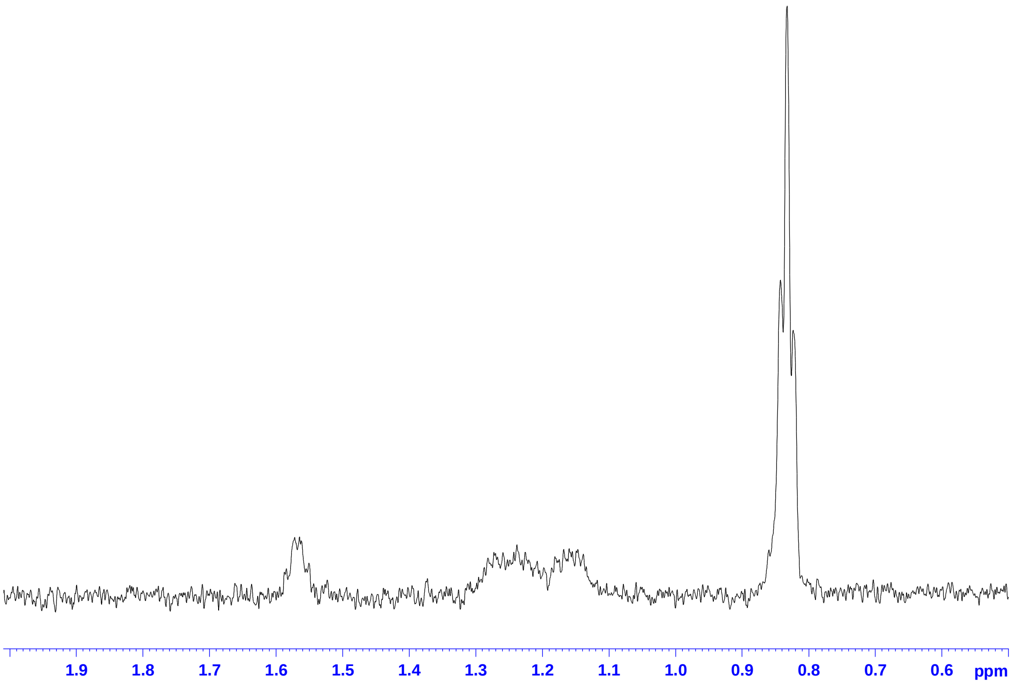

Figure S41. ^{1}H NMR spectrum of (*S*)-MTPA ester **7a** in CD_3OD (700 MHz).

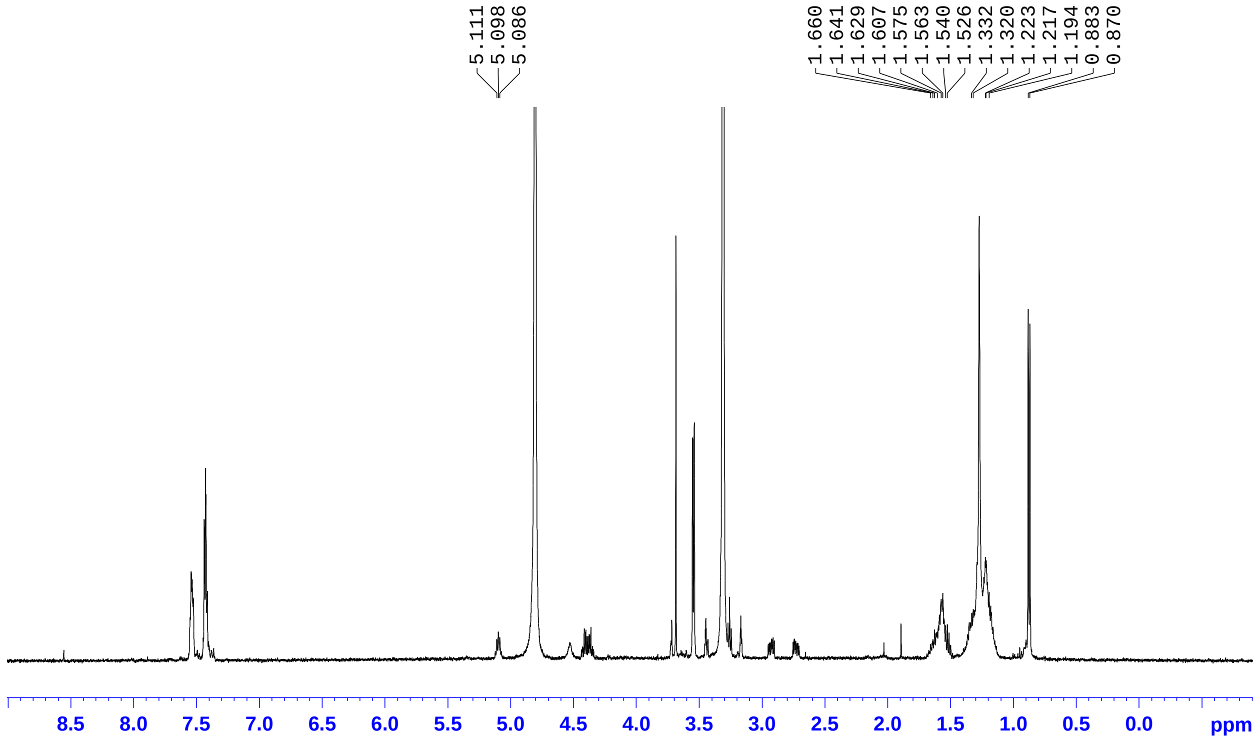

Figure S42. ^{1}H NMR spectrum of (*R*)-MTPA ester **7b** in CD_3OD (700 MHz).

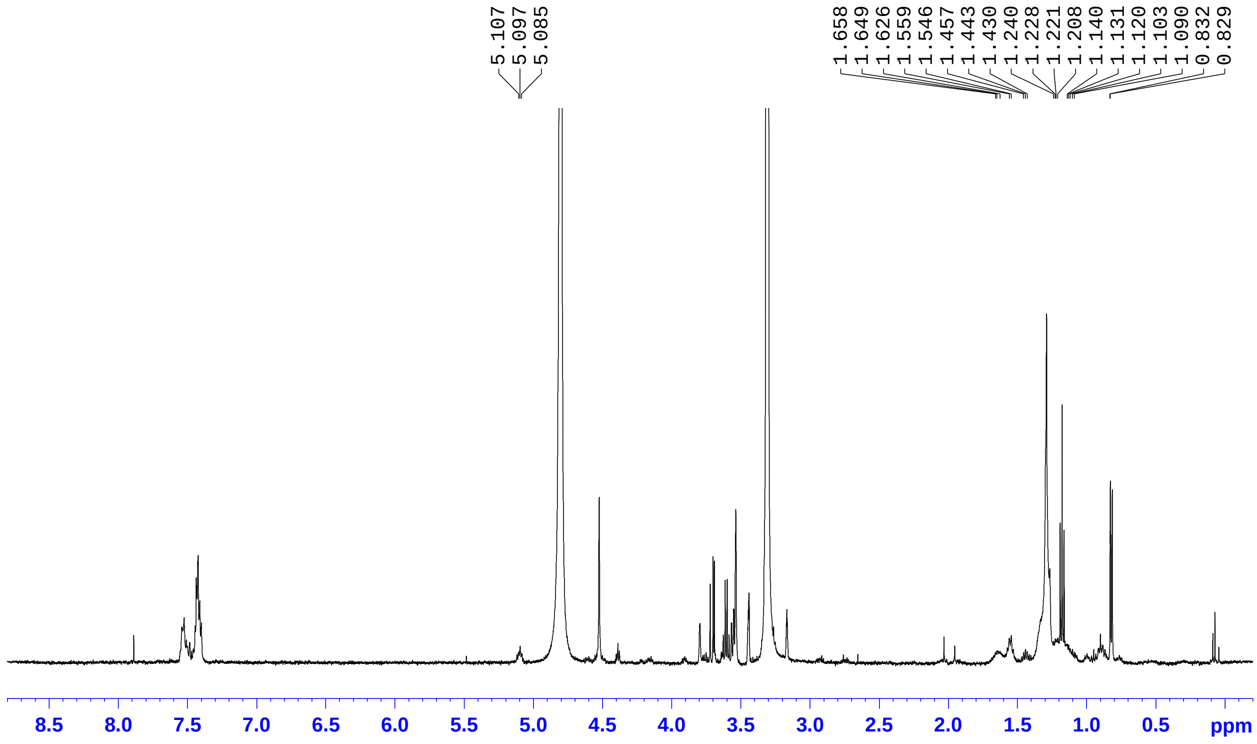

Figure S43. 1D selective TOCSY spectrum of (*S*)-MTPA ester **7a** with selective excitation of H_3-19 (700 MHz, CD_3OD).

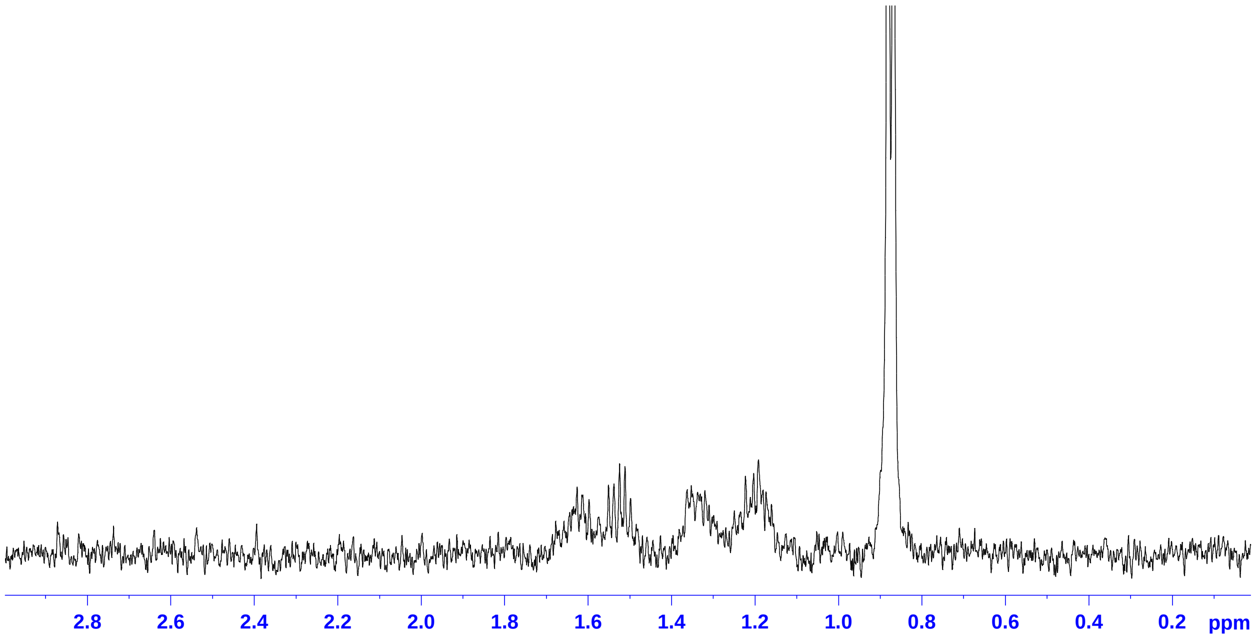

Figure S44. 1D selective TOCSY spectrum of (R)-MTPA ester **7b** with selective excitation of H3-19 (700 MHz, CD_3OD).

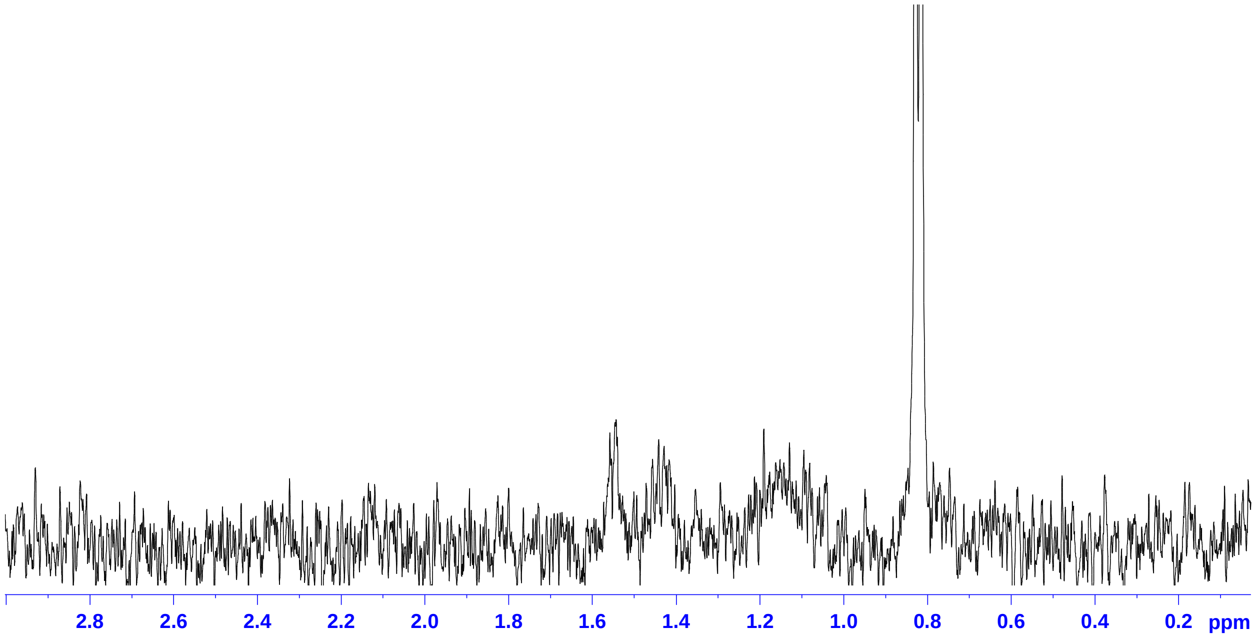

Figure S45. Images of the sample and spicules of the sponge *Antho (Acarnia) ridgwayi* Stone, Lehnert & Hoff, 2019 (order Poecilosclerida, family Microcionidae, the registration number PIBOC O47-142).

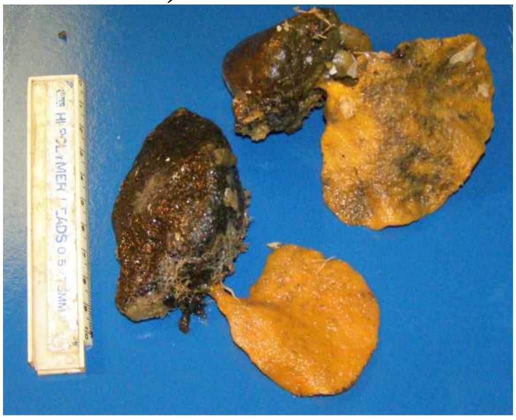

O47-142 *Antho (Acarnia) ridgwayi*

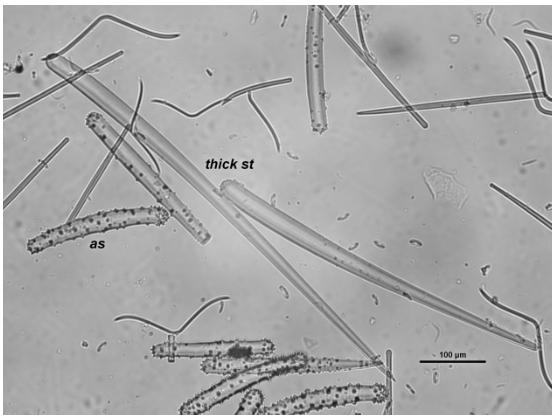

Thick styles with microspined heads (***thick st***). Acanthostrongyles (***as***)

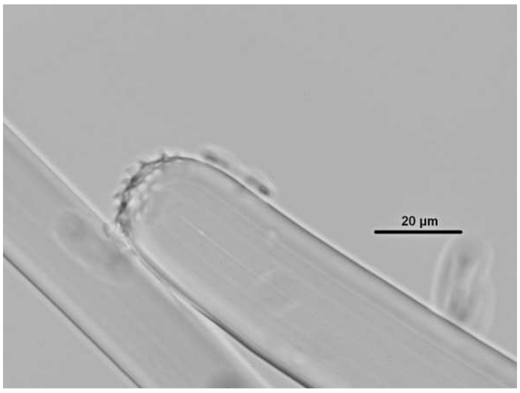

Microspined head of thick style

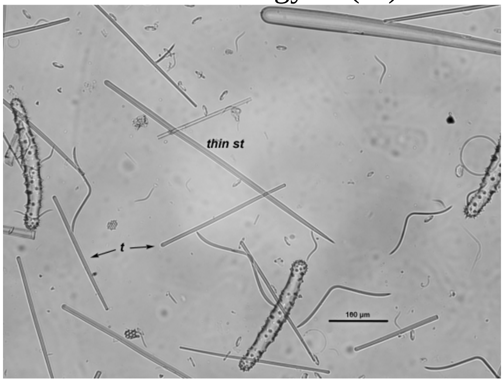

Thin style with microspined head (***thin st***). Tylotes with microspined heads (***t***)

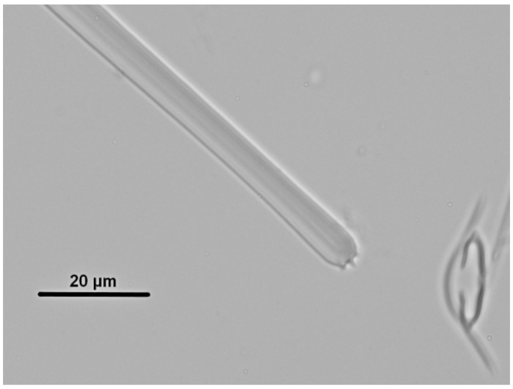

Microspined head of thin style

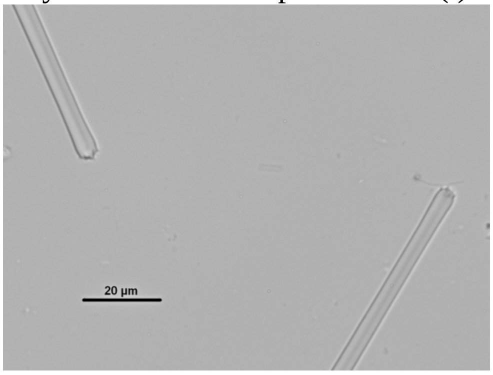

Microspined heads of tylotes

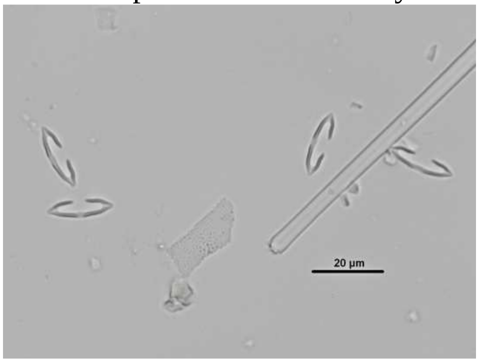

Palmate isochelae

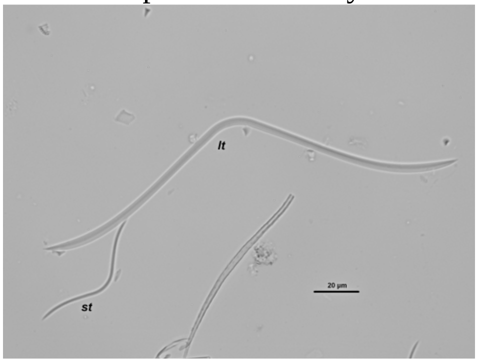

Small toxa (***st***) and large toxa (***lt***)
